# Genome sequencing of deep-sea hydrothermal vent snails reveals adaptions to extreme environments

**DOI:** 10.1093/gigascience/giaa139

**Published:** 2020-12-15

**Authors:** Xiang Zeng, Yaolei Zhang, Lingfeng Meng, Guangyi Fan, Jie Bai, Jianwei Chen, Yue Song, Inge Seim, Congyan Wang, Zenghua Shao, Nanxi Liu, Haorong Lu, Xiaoteng Fu, Liping Wang, Xin Liu, Shanshan Liu, Zongze Shao

**Affiliations:** Key Laboratory of Marine Biogenetic Resources, Third Institute of Oceanography, Ministry of Natural Resources, Daxue Road 178, Xiamen 361005, China; BGI-Qingdao, BGI-Shenzhen, Qingdao 266555, China; BGI-Shenzhen, Shenzhen 518083, China; Department of Biotechnology and Biomedicine, Technical University of Denmark, Anker Engelunds Vej 1, Lyngby 2800, Denmark; BGI-Qingdao, BGI-Shenzhen, Qingdao 266555, China; BGI-Qingdao, BGI-Shenzhen, Qingdao 266555, China; BGI-Shenzhen, Shenzhen 518083, China; State Key Laboratory of Agricultural Genomics, BGI-Shenzhen, Shenzhen 518083, China; BGI-Shenzhen, Shenzhen 518083, China; BGI-Qingdao, BGI-Shenzhen, Qingdao 266555, China; BGI-Qingdao, BGI-Shenzhen, Qingdao 266555, China; Integrative Biology Laboratory, College of Life Sciences, Nanjing Normal University, Wenyuan Road 1,Nanjing 210046, China; Comparative and Endocrine Biology Laboratory, Translational Research Institute-Institute of Health and Biomedical Innovation, School of Biomedical Sciences, Queensland University of Technology, Woolloongabba 4102, Australia; BGI-Qingdao, BGI-Shenzhen, Qingdao 266555, China; BGI-Qingdao, BGI-Shenzhen, Qingdao 266555, China; BGI-Shenzhen, Shenzhen 518083, China; BGI-Shenzhen, Shenzhen 518083, China; Key Laboratory of Marine Biogenetic Resources, Third Institute of Oceanography, Ministry of Natural Resources, Daxue Road 178, Xiamen 361005, China; Key Laboratory of Marine Biogenetic Resources, Third Institute of Oceanography, Ministry of Natural Resources, Daxue Road 178, Xiamen 361005, China; BGI-Qingdao, BGI-Shenzhen, Qingdao 266555, China; BGI-Shenzhen, Shenzhen 518083, China; China National GeneBank, BGI-Shenzhen, Shenzhen 518120, China; BGI-Qingdao, BGI-Shenzhen, Qingdao 266555, China; Key Laboratory of Marine Biogenetic Resources, Third Institute of Oceanography, Ministry of Natural Resources, Daxue Road 178, Xiamen 361005, China

**Keywords:** deep-sea snails, genome assembly, comparative genomics, biomineralization

## Abstract

**Background:**

The scaly-foot snail (*Chrysomallon squamiferum*) is highly adapted to deep-sea hydrothermal vents and has drawn much interest since its discovery. However, the limited information on its genome has impeded further related research and understanding of its adaptation to deep-sea hydrothermal vents.

**Findings:**

Here, we report the whole-genome sequencing and assembly of the scaly-foot snail and another snail (*Gigantopelta aegis*), which inhabits similar environments. Using Oxford Nanopore Technology, 10X Genomics, and Hi-C technologies, we obtained a chromosome-level genome of *C. squamiferum* with an N50 size of 20.71 Mb. By constructing a phylogenetic tree, we found that these 2 deep-sea snails evolved independently of other snails. Their divergence from each other occurred ∼66.3 million years ago. Comparative genomic analysis showed that different snails have diverse genome sizes and repeat contents. Deep-sea snails have more DNA transposons and long terminal repeats but fewer long interspersed nuclear elements than other snails. Gene family analysis revealed that deep-sea snails experienced stronger selective pressures than freshwater snails, and gene families related to the nervous system, immune system, metabolism, DNA stability, antioxidation, and biomineralization were significantly expanded in scaly-foot snails. We also found 251 H-2 Class II histocompatibility antigen, A-U α chain-like (*H2-Aal*) genes, which exist uniquely in the *Gigantopelta aegis* genome. This finding is important for investigating the evolution of major histocompatibility complex (MHC) genes.

**Conclusion:**

Our study provides new insights into deep-sea snail genomes and valuable resources for further studies.

## Background

The discovery of deep-sea hydrothermal vents in the late 1970s expanded our knowledge of the extent of life on Earth [1]. Deep-sea macrobenthos, which are animals that inhabit deep-sea hydrothermal vents, face high hydrostatic pressure, variable temperatures and pH, and high levels of hydrogen sulphide, methane, and heavy metals [2]. To date, the literature contains a limited number of studies on the genetics of macrobenthos. A recent report on the genome of deep-sea hydrothermal vent/cold seep mussels (*Bathymodiolus platifrons*) showed that, while most of the genes present in a related shallow-water mussel (*Modiolus philippinarum*) have been retained, many gene families have expanded in the *B. platifrons* genome. These families include those that are associated with stabilizing protein structures, removing toxic substances from cells, and the immune response to symbionts [3].

Gastropods represent the largest class of the phylum Mollusca, with different estimates of diversity varying from 80,000 to 150,000 species [4]. More than 218 gastropod (i.e., snail and slug) species have been described from chemosynthetic ecosystems (i.e., solely rely on endosymbiotic bacteria for sustenance), of which >138 are believed to be endemic to these ecosystems [5]. Gastropods are an important component of the fauna in hydrothermal vents in terms of abundance and biomass [6]. Owing to the lack of samples and fossil evidence, studies on the evolution and adaptation of deep sea chemosynthetic gastropods are very limited. The scaly-foot snail, *Chrysomallon squamiferum*, is only found in hydrothermal vents at a depth of ∼3,000 m in the Indian Ocean. There are 2 types of varieties: black (due to greigite, which is an iron sulphide mineral that covers its exterior) scaly-foot individuals from the Kairei field on the central Indian ridge and Longqi field on the Southwest Indian ridge, and white scaly-foot individuals from the Solitaire field on the Central Indian Ridge [7] and Wocan field on the Carlsberg Ridge of the northwest Indian ocean (this study). In particular, *C. squamiferum* has been included in the International Union for Conservation of Nature (IUCN) Red List of Endangered Species on 18 July 2019 [8]. Furthermore, the recently reported whole genome of the black scaly-foot snail highlighted its evolved defence mechanisms of biomineralized armour [9]. *Gigantopelta* is a major megafaunal gastropod genus found in some hydrothermal fields. The genus includes 2 species, *Gigantopelta chessoia* from East Scotia Ridge and *Gigantopelta aegis* from the Southwest Indian Ridge [6]. Both *Chrysomallon* and *Gigantopelta* are members of the family Peltospiridae. They live in high-density aggregations and share several features, such as a large body size (up to >45 mm, compared to typical sizes in other taxa of 10–15 mm, a 10–50 fold increase in body volume) and an enlarged oesophageal gland [10].

**Figure 1:**
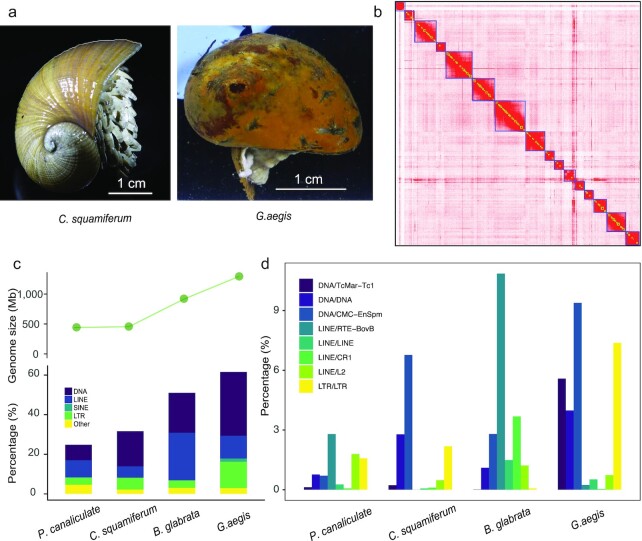
Genome characteristics of *C. squamiferum* and *G. aegis*. **a**, Photos of 2 species. Left: *C. squamiferum*; right: *G. aegis*. Scale bar = 1 cm. **b**, Heat map of chromatin interaction relationships at a 125-kb resolution of 16 chromosomes. **c**, Genome sizes and transposable elements in *C. squamiferum, G. aegis*, and 2 representative freshwater snail genomes. **d**, Distribution of repeat subtypes of 4 species.

In this study, we sequenced and assembled genomes of the white scaly-foot snail *Chrysomallon squamiferum* (NCBI:txid216257; marinespecies.org:taxname:736932) (Fig. 1a), which differ from the published genomes of the black varieties, from the Wocan field on the Carlsberg Ridge of the northwest Indian ocean and *Gigantopelta aegis* (NCBI:txid1735272; marinespecies.org:taxname:853164) (Fig. 1a) from the Longqi field on Southwest Indian Ridge. We gained insights into the evolution, gene family expansions, and adaptations of these extremophile gastropods.

## Data Description

### Genome assembly and annotation

The *C. squamiferum* genome was sequenced using a combination of sequencing libraries—10X Genomics, Oxford Nanopore Technologies (ONT), and Hi-C—to generate ∼369.03 Gb of raw data (Supplementary Table S1). Owing to the limited sample material, *G. aegis* was sequenced from whole-genome shotgun libraries (with 350 bp to 10 kb inserts on the BGISEQ-500) (BGISEQ-500, RRID:SCR_017979) to generate 910.08 Gb of raw data (Supplementary Table S2). The genome of *C. squamiferum* was assembled with long ONT reads by using Canu v1.7 (Canu, RRID:SCR_015880) [11] and WTDBG (WTDBG, RRID:SCR_017225) [12]. After polishing the genome with 10X Genomics sequencing data, a 454.58-Mb assembly (a little smaller than the estimated genome size: 495 Mb, Supplementary Fig. S1) with 6,449 contigs and an N50 of 541.32 kb was generated (Supplementary Table S3). Next, Hi-C data were used to anchor the assembly, yielding a 16-chromosome assembly (Fig. 1b). This effort increased the N50 size to ∼20.71 Mb (Table 1). The 16 chromosomes cover ∼80% of the whole genome, and the average length, maximal length, and minimal length of the 16 chromosomes were 22.67, 46.78, and 10.64 Mb, respectively (Supplementary Table S4). A BUSCO (BUSCO, RRID:SCR_015008) completeness score of 94.80% for this genome suggested that it was of good quality (Supplementary Table S5). A ∼1.29-Gb (a little smaller than the estimated genome size: 1.50 Gb, Supplementary Fig. S1) genome assembly of *G. aegis* with a scaffold N50 of 120.96 kb (Supplementary Table S6) and a BUSCO completeness score of 92.40% (Supplementary Table S7) was obtained using Platanus (Platanus, RRID:SCR_015531) [13]. After masking repeat elements, we used homologous and *de novo* prediction methods to construct gene models for the 2 genomes, obtaining 28,781 *C. squamiferum* genes and 25,601 *G. aegis* genes (Supplementary Tables S8 and S9). The gene sets were functionally annotated using KEGG (KEGG, RRID:SCR_012773), Swiss-Prot (UniProt, RRID:SCR_002380), InterPro (InterPro, RRID:SCR_006695), and TrEMBL (TrEMBL, RRID:SCR_002380) (Supplementary Tables S10 and S11).

**Table 1:** Genome assembly and annotation of *Chrysomallon squamiferum* and *Gigantopelta aegis*

Species	*Chrysomallon squamiferum*	*Gigantopelta aegis*
Genome size	455.36 Mb	1.29 Gb
Scaffold N50	20.71Mb	120.96 kb
Contig N50	541.32 kb	6.96 kb
No. of genes	28,781	25,601
Repeat content, %	30.56	64.17
GC content, %	34.48	37.45
Complete BUSCO, %	94.80	92.40

### Genome sizes and repeat contents

The genome assembly sizes of *C. squamiferum* (∼455.36 Mb) and *G. aegis* (∼1.29 Gb) differed from those of freshwater snails (∼916 Mb for *Biomphalaria glabrata* [14] and ∼440 Mb for*Pomacea canaliculata* [15]), which suggests that there is significant genome size diversity within snails (Fig. 1c). In the absence of ploidy effects [16, 17], differences in genome size often stem from the accumulation of various repetitive elements. A comparison of the repeat elements (Fig. 1c and Supplementary Table S12) supported this trend. The genomes of *C. squamiferum* and *P. canaliculata* (smaller genome sizes) contained fewer repeats than *B. glabrata* and *G. aegis*, whereas *G. aegis* had more repeats than *B. glabrata* (Fig. 1d). This finding suggests that snail genome sizes correlate with repeat content. Despite the similar genome sizes of *C. squamiferum* and *P. canaliculata*, their genome landscapes were distinct. For example, ∼10.17% of the *C. squamiferum* genome consisted of tandem repeats compared to ∼2.89% in *P. canaliculata* (Supplementary Table S12). DNA transposons and long terminal repeats (LTRs) comprise ∼17.73% and ∼5.99% of the *C. squamiferum* genome, respectively, but only ∼6.84% and ∼3.53% in *P. canaliculata*. Long interspersed nuclear elements (LINEs) made up ∼8.63% of the *P. canaliculata* genome compared to ∼5.65% in *C. squamiferum*. Similarly, although the larger *G. aegis* and *B. glabrata* genomes have similar proportions of tandem repeats, *G. aegis* had a higher percentage of DNA transposons (∼32.15% vs ∼20.20%) and LTRs (∼13.32% vs ∼3.75%). LINEs made up ∼23.93% of the *B. glabrata* genome compared to ∼11.51% in *G. aegis*. Taken together, these data suggest that deep-sea hydrothermal vent snail genomes have more DNA transposons and LTRs and fewer LINEs than their freshwater counterparts. In particular, DNA/CMC-EnSpm, DNA/TcMar−Tc1, and DNA/DNA were the main factors that caused the differences in DNA transposon content in the 4 snail genomes (Fig. 1d). We found that LINE/L2, LINE/RTE-BovB, LINE/LINE, and LINE/CR1 were much higher in fresh-water snail genomes than in deep-sea snails. Although most of the precise functions of these repeats have not been studied in depth, repeats have been thought to have a regulatory function in related genes that play an important role in the life cycle and can introduce great genome flexibility [18]. Also, in the mammalian genome, transposons have been described as redundant enhancers that regulate their target genes, which are more highly expressed or expressed in a specific tissue, indicating the importance of transposons [19]. Thus, we might infer that the expansion of DNA transposons and LTRs, as well as the absence of some LINEs, may be closely associated with important genes that help these deep-sea snails adapt to extreme environments.

### Construction of phylogenetic relationships for deep-sea snails

**Figure 2:**
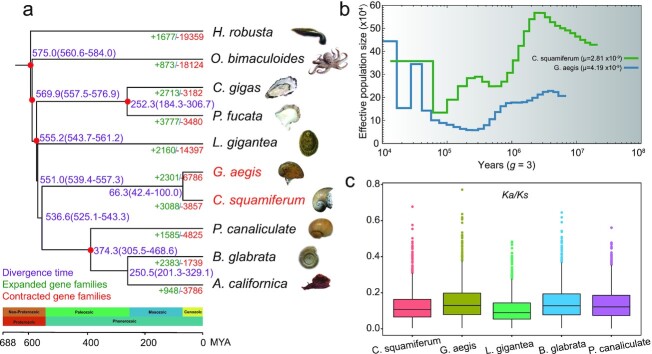
Phylogenetic tree, estimated *N_e_*, and evolution of single-copy orthologous genes of deep-sea snails. **a**, Phylogenetic tree of 10 representative molluscs. Expanded and contracted gene families were identified using CAFE. Divergence time was estimated using MCMCtree. Species names in red represent 2 deep-sea snails. Red dots represent calibration time from TimeTree database. Purple ranges in parentheses denote 95% CI(confidence interval).The timescale also refers to the TimeTree database. **b**, Estimated demographic histories of 2 deep-sea snails. The generation time set to “3” refers to the land snail [20]. The μ values are calculated in Supplementary Table S15. **c**, Box plot of Ka/Ks values for 5 species.

To determine the phylogenetic relationships between deep-sea snails and other molluscs, we compared their genomes with those from 2 shallow-water bivalves (*Pinctada fucata* and *Crassostrea gigas*) and 4 shallow-water gastropods, including 2 freshwater snails (*B. glabrata* and *P. canaliculata*), 1 limpet (*B. glabrata*), and 1 sea slug (*Aplysia californica*). The genomes of the California two-spot octopus (*Octopus bimaculoides*) and the freshwater leech (*Helobdella robusta*) were used as the outgroup (Fig. 2a). We identified 26,668 gene families in the 10 species examined (Supplementary Table S13). Phylogenetic trees were constructed from 406 shared single-copy orthologs. Both maximum likelihood (ML) and Bayesian methods revealed the same topology (Fig. 2a and Supplementary Fig. S2), which is consistent with a recent study [15]. In the tree, bivalves and gastropods are clearly separated and the 2 deep-sea snails are located on the same branch and are independent of other snails (although their genome sizes are quite different). We estimated that *C. squamiferum* and *G. aegis* diverged from a common ancestor ∼66.3 million years ago (MYA).

### Demographic histories of the deep-sea snails

Based on these 2 assembled genomes, we estimated their historical effective population size (*N_e_*) using whole-genome genetic variation. We identified ∼3.51 and ∼3.19 million heterozygous single-nucleotide polymorphisms (SNPs) with nucleotide diversities of 0.0077 and 0.0025 for *C. squamiferum* and *G. aegis*, respectively. We estimated changes in *N_e_* using the pairwise sequential Markovian coalescent (PSMC, RRID:SCR_017229) method, which can infer demography from ∼20,000 to 1 MYA [21]. The effective population sizes of *C. squamiferum* and *G. aegis*—species derived from different geographical locations in the Indian Ocean—are distinct (Fig. 2b). In the demographic history of *G. aegis N_e_* decreased until ∼250,000 years ago, followed by an *N_e_* increase, from ∼50,000 to 450,000 individuals, 20,000 years ago. Several cycles of increasing and decreasing *N_e_* have been observed for *C. squamiferum*, with the effective population size recovering and stabilizing at 35,000 individuals ∼70,000 years ago. Thus, although deep-sea habitats are inhabited, deep-sea snail populations are sensitive to habitat disturbances. It was reported that vent organisms are exquisitely sensitive to nuances in fluid flux, such as chemical compositions, temperature, geological setting, and biological interactions [22]. Our results revealed that the demographic histories of these 2 snails differed because their habitat conditions are markedly different.

### Evolution of single-copy orthologous genes

To explore the evolutionary rate of single-copy orthologous genes, we calculated the synonymous substitution rate (Ka) and nonsynonymous substitution rate (Ks) values of 1,324 single-copy orthologous genes shared by the 2 deep-sea snails, 1 shallow-water limpet (*Lottia gigantea*), and 2 freshwater snails (*B. glabrata* and *P. canaliculata*) using Codeml in the PAML package (PAML, RRID:SCR_014932) [23] (Fig. 2c, Supplementary Fig. S3 and Table S15). We found that the Ka values of the 2 deep-sea snails (average: 0.37 and 0.41) were higher (Mann-Whitney test, *P* < 0.001) than that of the shallow-water limpet (0.35) but similar to those of 2 freshwater snails (0.39 and 0.41), which suggests that the genes of deep-sea and freshwater snails both evolved faster after their divergence from the shallow-water limpet. The Ks values of the deep-sea (3.34 and 3.09) and freshwater (3.19 and 3.24) snails were also similar to and lower (Mann-Whitney test, *P* < 0.001) than those of the shallow-water limpet (3.72). Additionally, the Ka/Ks values of the deep-sea snails (average: 0.13 and 0.15) were ∼20% and ∼40% higher (Mann-Whitney test, *P*< 0.001) than those of the shallow-water limpet (0.11). From these findings, we could infer that deep-sea snails have experienced stronger selective pressures than the shallow- and freshwater species discussed here, possibly to allow adaptation to life in hydrothermal vents.

### Expanded gene families in deep-sea snail genomes

#### Nervous system

**Figure 3:**
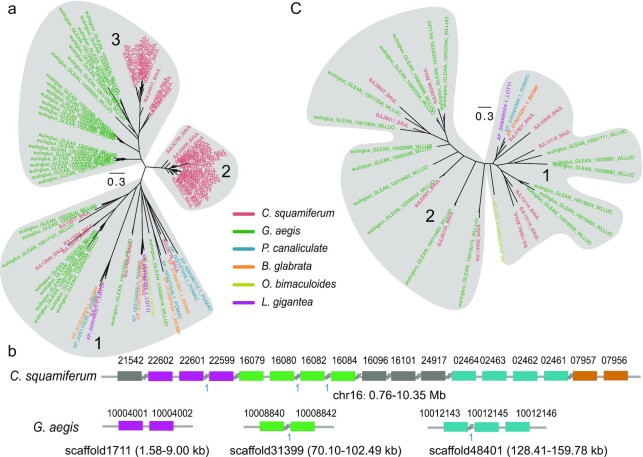
Expansion of nervous system–related genes. **a**, Phylogentic tree of *BTBD6* genes in the examined species. The grey ellipses mark different clusters of genes. **b**, Expansion pattern of *BTBD6* genes in 2 deep-sea snails. Grey lines represent scaffold sequences. Coloured rectangles represent *BTBD6* genes. Symbols “//” represent other genes along the scaffolds. The blue numbers “1” represent only 1 gene between the tandem duplicated genes. **c**, Expansion of *HTR4* genes. The species legend in the middle applies to **a** and **c**. Gene trees of **a** and **c** were constructed using MUSCLE (v3.8.31) [72] and FastTree (v2.1.10) [27].

Using CAFE (CAFE, RRID:SCR_005983) [24] (see details in Methods), we identified 2 significantly (*P*< 0.01) expanded gene families in the 2 deep-sea snail genomes compared to the freshwater snails and shallow-water limpet. The BTB/POZ domain-containing protein 6 (*BTBD6*) had 56 copies in *C. squamiferum* and 35 copies in *G. aegis*, while <5 copies were found in the 4 other snail species examined (Fig. 3a). We found 17 *BTBD6* genes on chromosome 16 of *C. squamiferum*, and these genes showed traces of tandem duplications (Fig. 3b). In *G. aegis*, we also found several tandem gene clusters (Fig. 3b). *HTR4* (5-hydroxytryptamine receptor 4) had 12 copies in *C. squamiferum* and 18 copies in *G. aegis*, while only 1 copy was found in the other snail species (Fig. 3c). The expansions of these gene families also displayed tandem duplications (Supplementary Fig. S4). Both of these genes play roles in neuroregulation; *BTBD6* is an adaptor of the Cul3 ubiquitin ligase complex and is essential for neural differentiation [25], while *HTR4* modulates the release of various neurotransmitters [26]. A previous study revealed that a large unganglionated nervous system exists in *C. squamiferum* [7]. We speculate that the expansions of *BTBD6* and *HTR4* contribute to this system by sustaining life in a deep-sea environment.

#### Metabolism-related genes

**Figure 4:**
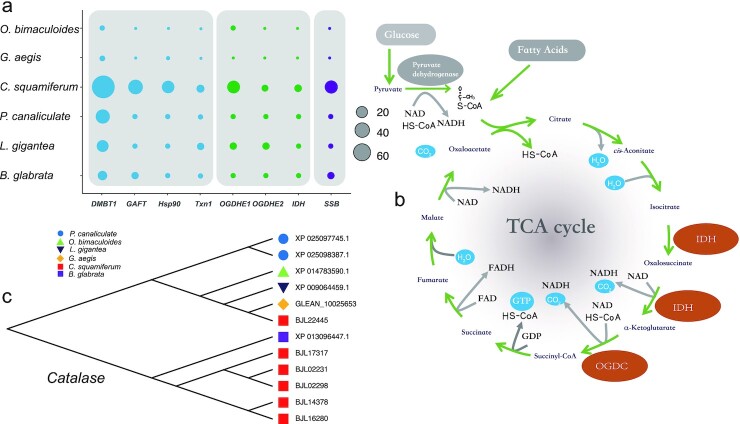
Expansion of immune, metabolism, DNA stability, and antioxidation genes. **a**, Gene numbers of 4 defence-related genes (*DMBT1, GAFT, Hsp90*, and *Txn1*), 3 metabolism-related genes (*OGDHE1, OGDHE2*, and *IDH*), and the *SSB* gene. **b**, TCA cycle signal pathway. The brown ellipses represent important enzymes and the expansion of these genes (*OGDHE1, OGDHE2*, and *IDH*). **c**, Expansion of the catalase (*CAT*) gene family in selected species.

*C. squamiferum* houses abundant endosymbionts in its greatly enlarged oesophageal gland, and these endosymbionts supply nutrition for its host. KEGG enrichment analysis on the 183 expanded gene families of *C. squamiferum* revealed significant enrichment for metabolic pathways (*q*-value < 0.0001, Supplementary Table S16). Among these genes, 9 gene families encoded enzymes in the glycolysis pathway and citrate cycle (TCA cycle). For example, the genes for isocitrate dehydrogenase (IDH), which catalyses the oxidative decarboxylation of isocitrate to produce α-ketoglutarate and carbon dioxide, expanded significantly (*P* < 0.01). The α-ketoglutarate dehydrogenase complex (OGDC) consists of 3 components: oxoglutarate dehydrogenase (OGDH), dihydrolipoyl succinyltransferase (DLST), and dihydrolipoyl dehydrogenase (DLD), among which the genes for OGDH were expanded (*P* < 0.01, Fig. 4a). IDH and OGDC are 2 rate-limiting enzymes in the TCA cycle, and the related biochemical reactions are irreversible (Fig. 4b).

#### Defence mechanisms

Endosymbiotic bacteria are critical for snail life in deep-sea hydrothermal vent ecosystems [28]. These bacterial taxa are largely restricted to chemosynthetic environments, with some being exclusive to vents [29]. The divergent evolution of the *C. squamiferum* and *G. aegis* genomes may have generated diverse defence mechanisms.

A total of 183 expanded gene families were identified in the *C. squamiferum* genome. As expected, many of these families have roles in the immune system. However, unlike the freshwater snail *B. glabrata* [14] and deep-sea mussels [3], we did not detect an expansion of the Toll-like receptor 13 (*TLR13*) gene family, but identified other expanded gene families (Fig. 4a). For example, increased expression of thioredoxin 1 (*Txn1*; 22 copies in *C. squamiferum*) was identified. Thioredoxin 1 (*Txn1*), a redox protein, is important for regulating of cellular redox homeostasis and anti-apoptotic functions. Txn1 stimulates cell proliferation and cell cycle progression, induces hypoxia-inducible factor-1α (HIF-1α) and angiogenesis, and alters the balance between the matrix metalloproteinases and their tissue inhibitors [30, 31]. Txn1 also plays a pivotal role in T-cell activation in mice [32]. Although T-cell–related adaptive immunity only appears in vertebrates, the existence and expansion of this gene may assist the innate immune system of *C. squamiferum*. Glutamine-fructose-6-phosphate transaminase (*GAFT*; 21 copies in *C. squamiferum*) promotes the biosynthesis of chitin [33, 34], which is one of the stable components of the crustacean shell and provides protection against predation and infection.

We identified expanded gene families that maintain the stability of nucleic acids and proteins, such as heat shock protein 90 (Hsp90; 13 copies in *C. squamiferum*, Fig. 4a), which protects proteins against heat stress [35]; the single-stranded DNA-binding proteins, encoded by SSB genes (19 copies in *C. squamiferum*, and 1 copy in other species, Fig. 4a), which are required for DNA replication, recombination, and repair processes [36]; and catalase (*CAT*, 6 copies *C. squamiferum*; Fig. 4c), which is critical in the response against oxidative stress [37]. The elevated levels of heavy metals and sulphide and high temperatures in hydrothermal vents are likely to greatly increase the risk of DNA damage and misfolded proteins. Thus, these expanded gene families may help these snails resist environmental stress.

We also found a special gene family, deleted in malignant brain tumours 1 (*DMBT1*), expanded (70 copies, Fig. 4a) in the *C. squamiferum* genome. *DMBT1* can encode 3 glycoproteins (DMBT1 [deleted in malignant brain tumours 1 protein], SAG [salivary agglutinin], and GP340 [lung glycoprotein-340]) and belongs to the scavenger receptor cysteine-rich (SRCR) protein superfamily of the immune system [38]. This gene consists of the SRCR, CUB, and zona pellucida domains, and all 70 copies of this gene in *C. squamiferum* contain the SRCR domain, which can bind a broad range of pathogens, including cariogenic *streptococci, Helicobacter pylori*, and HIV [39]. However, previous studies have shown that SRCR domains that contain proteins are commonly expressed in the shell martrix [40] and have been proven to be potentially linked to biomineralization [41], which would be associated with the foot scales of *C. squamiferum*. Nonetheless, the expansion of this gene family will either strengthen the immune ability or help construct the scale armour of these snails.

Correspondingly, we identified the expansion of 198 gene families (containing 4,515 genes) in the *G. aegis* genome. These families were enriched in 58 KEGG pathways (*q*-value < 0.05) (Supplementary Table S17). The majority of these pathways were associated with the immune and disease response, and included terms such as “infection,” “NOD-like receptor signalling,” “Tumour necrosis factor (TNF) signalling pathway,” and “Antigen processing and presentation” (Supplementary Fig. S5). Surprisingly, we found 251 copies of the H-2 Class II histocompatibility antigen, A-U α chain-like (H2-Aal) genes, which is one of the major histocompatibility complex (MHC) genes in vertebrates [42]. The existence and super-expansion of this gene family in the invertebrate positions in *G. aegis* is useful for the study of immune system evolution.

## Discussion

Molluscs are a highly diverse group, and their high biodiversity makes them an excellent model to address topics such as biogeography, adaptability, and evolutionary processes [43]. Members of the family Peltospiridae in the gastropod clade Neomphalina are restricted to chemosynthetic ecosystems and, so far, are only known from hot vents [6]. Based on the chromosome-scale genome assembly analyses of the scaly-foot snail (*C. squamiferum*) and deep-sea snail (*G. aegis*), which both belong to the Peltospiridae family from chemosynthetic ecosystems, our results provide insight into the possible evolution and adaptation mechanisms of hydrothermal vent animals.

By constructing a phylogenetic tree, we found that snails diverged from other molluscs ∼555.2 MYA (Fig. 2a). These 2 deep-sea snails were found to be independent of other shallow-water gastropods ∼536.6 MYA. At the end of the Cretaceous geological period, ∼66.3 MYA, *C. squamiferum* and *G. aegis* diverged from each other and later had different *N_e_* (Fig. 2b). This finding indicated that they faced different environmental factors and selective pressures. This evolutionary time frame implies that the last common ancestor of all molluscs had already lived before the Cambrian Explosion (530–540 MYA), which was also speculated by the palaeobiological hypothesis [44]. It also elucidated that deep-sea gastropod lineages originated at least ∼540 MYA and diverged from other gastropods in the same age of the oldest mollusc taxons, Aculifera and Conchifera [45, 46]. The deep-sea gastropod lineages were also confirmed by the phylogenetic analysis of mitogenomes [47]. Further confirmed by the evolutionary rate of single-copy orthologous genes, deep-sea gastropod lineages have experienced stronger selective pressures than shallow-water gastropods (Fig. 2c).

Transposable elements (TEs) play multiple roles in driving genome evolution in eukaryotes [48]. The genome sizes of 4 representative snails were quite divergent (440 Mb to 1.29 Gb). The deep-sea snail *G. aegis* had the largest genome (1.29 Gb), with the highest percentage of DNA transposons (32.15%). Deep-sea snails (*C. squamiferum* and *G. aegis*) had more DNA transposons and LTRs than other snails but fewer LINEs. LTR class has been identified as the main contributor to open chromatin regions and transcription factor binding sites [49, 50]. LINEs may be associated with the duplicability of genomic regions, which are always shared between related lineages [51]. Thus, the higher portions of DNA transposons and LTRs may be the results of genome evolution due to environmental changes and associated with the ability of deep-sea snails to adapt to extreme environments.

Specifically, we analysed expanded gene families in deep-sea snail genomes (Fig. 4a). They both significantly expanded the nervous system, especially *BTBD6* and *HTR4*, which are involved in the neuroregulation of activities, such as movement, predation, and resistance to environmental change. As for the chemosynthetic snails, they both had expanded immune system–related gene families. In the *C. squamiferum* genome, the expansions of *Txn1* and *GAFT* were found. In the *G. aegis* genome, different immune and disease response gene families were expanded, such as *H2-Aal* genes. These expanded gene families were different from those found in freshwater snails and deep-sea mussels.

Interestingly, in the scaly-foot snail (*Chrysomallon squamiferum*) genome, genes involved in the main metabolic pathways were significantly enriched, including the glycolysis pathway and the citrate cycle (TCA cycle). Other enriched gene families included the single-stranded DNA-binding protein (*SSB)* family, which stabilize single-stranded DNA; heat shock protein 90 (*Hsp90*) family, which keep proteins folded properly; and the catalase (*CAT*) family, which prevents the generation of free radicals due to exposure to peroxides. The expansions of these gene families may have provided deep-sea snails with better immune reactions with symbionts, rapid nerve signal conduction, stronger metabolism, and effective resistance while adapting to their hydrothermal vent habitat.

In particular, we found that *DMBT1* gene families that encode multiple SRCR domains were expanded significantly in *C. squamiferum*. These genes play important roles in immune response and biomineralization, both of which are vital for deep-sea chemosynthetic snails.

In conclusion, the genome analysis of deep-sea snails (*C. squamiferum* and *G. aegis*) from hydrothermal vents revealed mechanisms of their evolution and molecular adaptations to extreme environments, and will be a valuable resource for studying the evolution of invertebrates.

## Methods

### Sample collection and DNA isolation

*C. squamiferum* samples were obtained from the Wocan vent field (60 31.410 E,6 21.410 N, 2,919 m depth) on the Carlsberg Ridge, northwest Indian Ocean, in March 2017 during the Chinese DY38th cruise. *G. aegis* samples were obtained from the Longqi vent field (49 38.969 E,37 47.025 S, 2,780 m) on the southwest Indian ridge in March 2015 during the Chinese DY35th cruise. DNA was extracted from the muscle sample of 1 individual using the cetyl trimethylammonium bromide (CTAB) method and a DNeasy blood & tissue kit (QIAGEN). DNA quality and quantity were checked using pulsed-field gel electrophoresis and a Qubit Fluorometer (Thermo Scientific).

### Library preparation and sequencing

#### Whole-genome shotgun sequencing

Four whole-genome sequencing (WGS) libraries were prepared for sequencing: 1 short insert size library (350 bp) and 3 mate-pair large insert size libraries (2, 5, and 10 kb). Libraries were constructed using an MGI Easy FS DNA Library Prep Set kit (MGI, China). Paired-end reads (100 bp) and mate-pair reads (50 bp) were obtained from the BGISEQ-500 platform.

#### 10X Genomics sequencing

To prepare the Chromium library, 1 ng of high-quality DNA was denatured, spiked into the reaction mix, and mixed with gel beads and emulsification oil to generate droplets within a Chromium Genome chip. Then, the rest of the steps were completed following the standard protocols for performing PCR. After PCR, the standard circularization step for BGISEQ-500 was carried out, and DNA nanoballs (DNBs) were prepared [52]. Paired-end reads with a length of 150 bp were generated on the BGISEQ-500 platform [53].

#### Oxford Nanopore Technologies

DNA for long-read sequencing was isolated from the muscle tissues of our samples. Using 5 flow cells and the ONT chemistry for the GridION X5 sequencer (GridION, RRID:SCR_017986). following manufacturer's protocols, we generated 39.61 Gbp of raw genome sequencing data.

### Hi-C library and sequencing

The Hi-C library was prepared following the standard *in situ* Hi-C [54] protocol for muscle samples, using DpnII (NEB, Ipswich, MA, USA) as the restriction enzyme. After that, a standard circularization step was carried out, followed by DNA nanoballs (DNB) preparation following the standard protocols of the BGISEQ-500 sequencing platform as previously described [52]. Paired-end reads with a length of 100 bp were generated on the BGISEQ-500 platform [53].

### Genome assembly

For the genome assembly of *C. squamiferum*, Canu v1.7 was first used to perform corrections of ONT reads with the parameters “correctedErrorRate=0.105 corMinCoverage=0 minReadLength=1000 minOverlapLength=800.” Then, wtdbg (v1.2.8) was used to assemble the genome with the parameters “–tidy-reads 3000 -k 0 -p 21 -S 4 –rescue-low-cov-edges” using corrected reads generated by Canu. Next, we made use of the sequencing reads from the 10X Genomics library to carry out genome polishing using Pilon v1.22 (Pilon, RRID:SCR_014731) with its default parameters. Quality control of Hi-C sequencing reads was first performed using the HiC-Pro pipeline (HiC-Pro, RRID:SCR_017643) [55] with the parameters “[BOWTIE2_GLOBAL_OPTIONS = –very-sensitive -L 30 –score-min L, -0.6, -0.2 –end-to-end –reorder; BOWTIE2_LOCAL_OPTIONS = –very-sensitive -L 20 –score-min L, -0.6, -0.2 –end-to-end –reorder; IGATION_SITE = GATC; MIN_FRAG_SIZE = 100; MAX_FRAG_SIZE = 100 000; MIN_INSERT_SIZE = 50; MAX_INSERT_SIZE = 1500].” In total, 23,646,810 pairs of valid reads were obtained. Next, the valid Hi-C data were used to anchor the nanopore contigs onto chromosomes separately by applying the 3D-DNA pipeline [56]. The contact maps were then generated by the Juicer pipeline [57], and the boundaries for each chromosome were manually rectified by visualizing the inter.hic file in Juicebox [58]. Sixteen chromosomes were identified by combining the linkage information from the agp file.

For the genome assembly of *G. aegis*, we obtained only WGS sequencing reads because of limited DNA and tissue samples. Platanus v1.2.4 [13] was used to assemble the genome with WGS clean data with the parameters “assemble –k 29 –u 0.2, scaffold -l 3 -u 0.2 -v 32 -s 32 and gap_close –s 34 –k 32 –d 5000.” BUSCO v2 weas used to evaluate genome assemblies with the metazoan_odb9 database.

### Genome annotation

#### Repeat annotation

Homolog-based and *de novo* prediction methods were used to detect repeat contents. In particular, RepeatMasker v4.0.5 (RepeatMasker, RRID:SCR_012954) [59] and RepeatProteinMask v4.0.5 (RepeatProteinMask, RRID:SCR_012954) were used to detect TEs against the Repbase database [60] at the nuclear and protein levels, respectively. RepeatMasker was used again to detect species-specific TEs against databases generated by RepeatModeler v1.0.8 (RepeatModeler, RRID:SCR_015027) and LTR-FINDER v1.0.6 (LTR-FINDER, RRID:SCR 01 5247) [61]. Moreover, Tandem Repeat Finder v4.0.7 [62] was used to predict tandem repeats.

#### Gene annotation

We combined homology-based and *de novo* evidence to predict protein-coding genes in 2 genomes. For the homology-based method, we used 6 relative gene sets of *A. californica, B. platifrons, B. glabrata, L. gigantea, M. philippinarum*, and *P. canaliculata*. First, these homologous protein sequences were aligned onto each assembled genome using TBLASTN (TBLASTN, RRID:SCR_011822), with an *E*-value cut-off of 1 × 10^−5^, and the alignment hits were linked to candidate gene loci by GenBlastA [63]. Second, we extracted genomic sequences of candidate gene regions, including 2-kb flanking sequences, and then used GeneWise v2.2.0 (GeneWise, RRID:SCR_015054) [64] to determine gene models.

In the *de novo* method, we used Augustus (Augustus, RRID:SCR_008417) [65] to predict the gene models on repeat-masked genome sequences. We selected high-quality genes with intact open reading frames (ORFs) and the highest GeneWise [64] score from a homology-based gene set to train Augustus with default parameters before prediction. Gene models with incomplete ORFs and small genes with protein-coding lengths <150 bp were filtered out. Finally, a BLASTP (BLASTP, RRID:SCR_001010) search of predicted genes was performed against the Swiss-Prot database [66]. Genes with matches to Swiss-Prot proteins containing any one of the following keywords were filtered: transpose, transposon, retrotransposon, retrovirus, retrotransposon, reverse transcriptase, transposase, and retroviral. Finally, the results of the homology- and *de novo*–based gene sets were merged using GLEAN (GLEAN, RRID:SCR_002890) [67] to yield a nonredundant reference gene set.

#### Gene function annotation

We annotated the protein-coding genes by searching against the following public databases: Swiss-Prot [68], KEGG [69], InterPro [70], and TrEMBL [68].

### Phylogenetic tree reconstruction and divergence time estimation

The TreeFam tool (Tree families database, RRID:SCR_013401) [71] was used to identify gene families as follows: first, all the protein sequences from a selection of 10 representative species (8 species including *Aplysia californica* [GCF_000002075.1], *Octopus bimaculoides* [GCF_0 011 94135.1], *Biomphalaria glabrata* [GCF_000457365.1], *Crassostrea gigas* [GCF_000297895.1], *Lottia gigantea* [GCF_000 327385.1], *Pomacea canaliculata* [GCF_003073045.1], *Pinctada fucata* [GCA_0 022 16045.1], and *Helobdella robusta* [GCF_000 326865.1] from the NCBI database and *C. squamiferum* and *G. aegis* from this research) were compared using blastp with the *E*-value threshold set as 1e−7. Then, alignment segments of each protein pair were concatenated using the in-house software Solar v0.9.6 [71]. H-scores were computed on the basis of Bit-scores and were used to evaluate the similarity among proteins. Finally, gene families were obtained by clustering homologous gene sequences using Hcluster_sg v0.5.0 [71].

We obtained 406 one-to-one single-copy orthology gene families based on gene family classification. Then, these gene families were extracted and aligned using guidance from amino acid alignments created using the default parameters of the MUSCLE (MUSCLE, RRID:SCR_011812) [72] programme. All sequence alignments were then concatenated to construct 1 super-matrix and then a phylogenetic tree was constructed under a GTR+gamma model for nucleotide sequences using ML and Bayesian methods. The same set of codon sequences were used for phylogenetic tree construction and estimation of divergence time. The PAML mcmctree programme [73, 74] was used to determine divergence times with the approximate likelihood calculation method, and the correlated molecular clock and REV substitution model. The concatenated coding sequences of one-to-one orthologous genes and the phylogenomics topology were used as inputs. We used 5 calibration time points based on fossil records: *A. californica*—*C. gigas* (∼516.3–558.3 MYA), *A. californica—P. canaliculata* (∼310–496 MYA), *A. californica—O. bimaculoides* (∼551–628 MYA), *C. gigas*—*H. robusta* (∼585–790 MYA), and *C. gigas—P. fucata* (394  MYA) [75] were used as constraints in the MCMCTree estimation.

### SNP calling and estimation of history population sizes

Approximately 50× clean WGS reads were mapped to genomes of *C. squamiferum* and *G. aegis* using BWA-MEM (v0.7.12-r1039) (BWA, RRID:SCR_010910) [76] with default parameters, respectively. Then, SAMtools (v0.1.19–44428cd) (Samtools, RRID:SCR_002105) [77] and “SortSam.jar” in the Picard package (v1.54) was used to convert and sort BAM files. Local realignment was again carried out using RealignerTargetCreator and IndelRealigner in GATK v3.6 (GATK, RRID:SCR_001876) [78] with default parameters. SNPs were identified using HaplotypeCaller and filtered using VariantFiltration with parameters “-filter-expression “QD < 2.0 || MQ < 40.0 || ReadPosRankSum < -8.0 || FS > 60.0 –filter-name LowQualFilter –genotype-filter-expression DP < 5.0” –genotype-filter-name lt_5.” Estimation of the historical effective population sizes was carried out using PSMC v0.6.5-r67 [21]. First, diploid genome references were constructed using SAMtools and BCFtools call with “samtools mpileup -C50” and “vcfutils.pl vcf2fq -d 20 -D 100.” Second, the demographic history was inferred using PSMC with parameters “-N25 -t15 -r5 -p 4+25*2+4+6” [79].

### Expansion and contraction of gene families

We used CAFE v2.1 [24] to analyse gene family expansion and contraction under the ML framework. The gene family results from the TreeFam pipeline and the estimated divergence time between species were used as inputs. We used the parameters “-p 0.01, -r 10 000, -s” to search for the birth and death parameter (λ) of gene families, calculated the probability of each gene family with observed sizes using 10,000 Monte Carlo random samplings, and reported birth and death parameters in gene families with probabilities <0.01.

## Data Availability

The genome assemblies of these 2 genomes have been deposited in GenBank under accession No. CNP0000854. The raw sequencing reads were also uploaded to the SRA database under accession No. CNP0000854. All supporting data are available in the *GigaScience* GigaDB database [80].

## Additional Files

Supplementary Figure S1.17-mer frequency distribution for C. squamiferum and G. aegis genomes.

Supplementary Figure S2.Construction of Phylogenetic trees for ten representative molluscs using coding sequences of 407 single-copy orthologs.

Supplementary Figure S3.Box plot of Ka and Ks values of 1,324 single copy orthologous genes from two deep-sea snails, one shallow-water snail, and two fresh-water snails.

Supplementary Figure S4.Expansion pattern of HTR4 genes in two deep-sea snails.

Supplementary Figure S5.KEGG enrichment analysis of unique gene families of G. aegis.

Supplementary Table S1.Statistics of raw sequencing data of Chrysomallon squamiferum.

Supplementary Table S2.Statistics of raw sequencing data of Gigantopelta aegis.

Supplementary Table S3.Summary from the genome assembly of Chrysomallon squamiferum without using Hi-C data.

Supplementary Table S4.Lengths of the 16 chromosomes assembled for Chrysomallon squamiferum.

Supplementary Table S5.BUSCO assessment of the assembled genome of Chrysomallon squamiferum using metazoa_odb9 database.

Supplementary Table S6.Summary of the genome assembly for Gigantopelta aegis.

Supplementary Table S7.BUSCO assessment of the assembled genome for Gigantopelta aegis using the metazoa_odb9 database.

Supplementary Table S8.General statistics of predicted protein-coding genes of Chrysomallon squamiferum.

Supplementary Table S9.General Statistics of Predicted Protein-coding Genes of Gigantopelta aegis.

Supplementary Table S10.Summary of predicted gene functions of the Chrysomallon squamiferum gene set.

Supplementary Table S11.Summary of predicted gene functions of the Gigantopelta aegis gene set.

Supplementary Table S12.Summary of repeat contents in four selected species.

Supplementary Table S13.Gene family clusters in selected species.

Supplementary Table S14.Estimation of mutation rates of two deep-sea snails.

Supplementary Table S15.Ka and Ks values of 1,324 single copy orthologous genes from five snails.

Supplementary Table S16.KEGG enrichment of expanded gene families of C. squamiferum.

Supplementary Table S17.KEGG enrichment of unique gene families in G. aegis genome.

## Abbreviations

bp: base pairs; BTBD6: BTB/POZ domain-containing protein 6; BUSCO: Benchmarking Universal Single-Copy Orthologs; BWA: Burrows-Wheeler Aligner; CAFE: Computational Analysis of gene Family Evolution; CAT: catalase; CR1: chicken repeat 1; DLD: dihydrolipoyl dehydrogenase; DLST: dihydrolipoyl succinyltransferase; DMBT1: deleted in malignant brain tumours 1; DNB: DNA nanoball; GAFT: glutamine-fructose-6-phosphate transaminase; GATK: Genome Analysis Toolkit; Gb: gigabase pairs; GP340: glycoprotein-340; Hi-C: high-throughput chromosome conformation capture; HIF-1α: hypoxia-inducible factor-1α; HIV: human immunodeficiency virus; Hsp90: heat shock protein 90; HTR4: 5-hydroxytryptamine receptor 4; IDH: isocitrate dehydrogenase; IUCN: International Union for Conservation of Nature; kb: kilobase pairs; KEGG: Kyoto Encyclopedia of Genes and Genomes; LINE: long interspersed nuclear element; LTR: long terminal repeat; Mb: megabase pairs; MHC: major histocompatibility complex; ML: maximum likelihood; MYA: million years ago; NCBI: National Center for Biotechnology Information; OGDC: α-ketoglutarate dehydrogenase complex; OGDH: oxoglutarate dehydrogenase; ONT: Oxford Nanopore Technologies; ORF: open reading frame; PAML: Phylogenetic Analysis by Maximum Likelihood; PSMC: pairwise sequential Markovian coalescent; SAG: salivary agglutinin; SNP: single-nucleotide polymorphism; SRA: Sequence Read Archive; SRCR: scavenger receptor cysteine-rich; TCA: tricarboxylic acid; TE: transposable element; TLR13: Toll-like receptor 13; TNF: tumour necrosis factor; Txn1: Thioredoxin 1; WGS: whole-genome sequencing.

## Competing Interests

The authors declare that they have no competing interests.

## Funding

This work was supported by the National Key R&D Programme of China (No. 2018YFC0310702).

## Authors' Contributions

Zongze Shao, S.L., G.F., and X.L. conceived and managed this project and amended the manuscript. X.Z., Y.Z., L.M., and I.S. performed the evolutionary analysis and wrote the manuscript. L.M., J.C., and Y.S. performed genome assembly and annotation. J.B., S.L., X.F., C.W., Zenghua Shao, H.L., N.L., and L.W. were responsible for sample collection, DNA extraction, and library construction.

## Supplementary Material

giaa139_GIGA-D-20-00187_Original_Submission

giaa139_GIGA-D-20-00187_Revision_1

giaa139_Response_to_Reviewer_Comments_Original_Submission

giaa139_Reviewer_1_Report_Original_Submission Takeshi Takeuchi, Ph.D -- 7/31/2020 Reviewed

giaa139_Reviewer_1_Report_Revision_1 Takeshi Takeuchi, Ph.D -- 10/9/2020 Reviewed

giaa139_Reviewer_2_Report_Original_Submission Mathew J. Jenny -- 8/5/2020 Reviewed

giaa139_Reviewer_3_Report_Original_Submission Reuben William Nowell, Ph.D. -- 8/10/2020 Reviewed

giaa139_Supplemental_Tables_and_Figures
