# Supplementary material for: Genome sequencing of deep-sea hydrothermal vent snails reveals adaptions to extreme environments
Source: Gigascience. 2020 Dec 15;9(12):giaa139. doi: 10.1093/gigascience/giaa139 (PMC7736800; doi:10.1093/gigascience/giaa139)
Supplement: giaa139_GIGA-D-20-00187_Original_Submission [file giaa139_giga-d-20-00187_original_submission.pdf]

GigaScience

Genome sequencing of deep-sea hydrothermal vent snails reveals adaptions to extreme environments

--Manuscript Draft--

**Manuscript Number:**	GIGA-D-20-00187
**Full Title:**	Genome sequencing of deep-sea hydrothermal vent snails reveals adaptions to extreme environments
**Article Type:**	Data Note
**Funding Information:**	National Key R&D Programme of China (No. 2018YFC0310702) — Dr. Xiang Zeng
**Abstract:**	Background<br><br>The scaly-foot snail (Chrysomallon squamiferum) is highly adapted to deep-sea hydrothermal vents and drew people's interest once it was found. However, the limited information on its genome inmedes related research and understanding of its adaptation to deep-sea hydrothermal vents.<br><br>Findings<br><br>Here, we report the whole-genome sequencing and assembly of the scaly-foot snail and another snail (Gigantopelta aegi), which inhabits similar environments. Using ONT, 10X genomic, and Hi-C technologies, we obtained a chromosome-level genome of C. squamiferum with an N50 size of 20.71 Mb. By constructing a phylogenetic tree, we found that these two deep-sea snails were independent of other snails, and their divergence from each other occurred approximately 66.3 million years ago. Comparative genomic analysis showed that different snails have diverse genome sizes and repeat contents. Deep-sea snails have more DNA transposons and LTRs, but fewer LINEs, than other snails. Gene family analysis revealed that deep-sea snails experienced stronger selective pressures than shallow-water snails, and the nervous system, immune system, metabolism, DNA stability, antioxidation and biomineralization-related gene families were significantly expanded in scaly-foot snails. We also found 251 class II histocompatibility antigen H2-Aal, which uniquely exist in the Gigantopelta aegi genome, which is important for investigating the evolution of MHC genes.<br><br>Conclusion<br><br>Our study provides new insights into deep-sea snail genomes and valuable resources for further studies.
**Corresponding Author:**	Yaolei Zhang<br>BGI<br>Qingdao, CHINA
**Corresponding Author Secondary Information:**	
**Corresponding Author's Institution:**	BGI
**Corresponding Author's Secondary Institution:**	
**First Author:**	Yaolei Zhang
**First Author Secondary Information:**	
**Order of Authors:**	Yaolei Zhang
	Xiang Zeng
	Lingfeng Meng
	Guangyi Fan

	Jie Bai
	Jianwei Chen
	Yue Song
	Inge Seim
	Congyan Wang
	Zenghua Shao
	Nanxi Liu
	Haorong Lu
	Xiaoteng Fu
	Liping Wang
	Xin Liu
	Shanshan Liu
	Zongze Shao
**Order of Authors Secondary Information:**	
**Additional Information:**	
**Question**	**Response**
Are you submitting this manuscript to a special series or article collection?	No
**Experimental design and statistics**<br><br>Full details of the experimental design and statistical methods used should be given in the Methods section, as detailed in our Minimum Standards Reporting Checklist. Information essential to interpreting the data presented should be made available in the figure legends.<br><br>Have you included all the information requested in your manuscript?	Yes
**Resources**<br><br>A description of all resources used, including antibodies, cell lines, animals and software tools, with enough information to allow them to be uniquely identified, should be included in the Methods section. Authors are strongly encouraged to cite Research Resource Identifiers (RRIDs) for antibodies, model organisms and tools, where possible.	Yes

Powered by Editorial Manager® and ProduXion Manager® from Aries Systems Corporation

Have you included the information requested as detailed in our Minimum Standards Reporting Checklist?	
**Availability of data and materials**<br><br>All datasets and code on which the conclusions of the paper rely must be either included in your submission or deposited in publicly available repositories (where available and ethically appropriate), referencing such data using a unique identifier in the references and in the “Availability of Data and Materials” section of your manuscript.<br><br>Have you have met the above requirement as detailed in our Minimum Standards Reporting Checklist?	Yes

Powered by Editorial Manager® and ProduXion Manager® from Aries Systems Corporation

main text Click here to download Manuscript Manuscript.docx

Genome sequencing of deep-sea hydrothermal vent snails reveals adaptions to extreme environments

Xiang Zeng[1†], Yaolei Zhang[2,3,4†], Lingfeng Meng[2†], Guangyi Fan[2,3,6], Jie Bai[3], Jianwei Chen[2], Yue Song[2] , Inge Seim[7,8], Congyan Wang[2], Zenghua Shao[2], Nanxi Liu[3], Haorong Lu[3], Xiaoteng Fu[1], Liping Wang[1],Xin Liu[2,3,5], Shanshan Liu[2*], Zongze Shao[1*]

[1]Key Laboratory of Marine Biogenetic Resources, Third Institute of Oceanography, Ministry of Natural Resources, No.178 Daxue Road, Xiamen 361005, China.

[2]BGI-Qingdao, BGI-Shenzhen, Qingdao 266555, China

[3]BGI-Shenzhen, Shenzhen, 518083, China

[4]Department of Biotechnology and Biomedicine, Technical University of Denmark, Lyngby, 2800, Denmark

[5]China National GeneBank, BGI-Shenzhen, Shenzhen 518120, China

[6]State Key Laboratory of Agricultural Genomics, BGI-Shenzhen, Shenzhen 518083, China.

[7]Integrative Biology Laboratory, College of Life Sciences, Nanjing Normal University, Nanjing, 210046, China

[8]Comparative and Endocrine Biology Laboratory, Translational Research Institute-Institute of Health and Biomedical Innovation, School of Biomedical Sciences, Queensland University of Technology, Woolloongabba, 4102, Australia

† These authors contributed equally.

*Correspondence: shaozz@163.com (Z.S.); liushanshan@genomics.cn (S.L.)

Abstract

Background

The scaly-foot snail (*Chrysomallon squamiferum*) is highly adapted to deep-sea hydrothermal vents and drew people's interest once it was found. However, the limited information on its genome inmedes related research and understanding of its adaptation to deep-sea hydrothermal vents.

Findings

Here, we report the whole-genome sequencing and assembly of the scaly-foot snail and another snail (*Gigantopelta aegi*), which inhabits similar environments. Using ONT, 10X genomic, and Hi-C technologies, we obtained a chromosome-level genome of *C. squamiferum* with an N50 size of 20.71 Mb. By constructing a phylogenetic tree, we found that these two deep-sea snails were independent of other snails, and their divergence from each other occurred approximately 66.3 million years ago. Comparative genomic analysis showed that different snails have diverse genome sizes and repeat contents. Deep-sea snails have more DNA transposons and LTRs, but fewer LINEs, than other snails. Gene family analysis revealed that deep-sea snails experienced stronger selective pressures than shallow-water snails, and the nervous system, immune system, metabolism, DNA stability, antioxidation and biomineralization-related gene families were significantly expanded in scaly-foot snails. We also found 251 class II histocompatibility antigen H2-Aal, which uniquely exist in the *Gigantopelta aegi* genome, which is important for investigating the evolution of MHC genes.

Conclusion

Our study provides new insights into deep-sea snail genomes and valuable resources for further studies.

Keywords: Deep-sea snails; Genome assembly; Comparative genomics; Biomineralization;

Background

The discovery of deep-sea hydrothermal vents in the late 1970s expanded our knowledge of the extent of life on Earth [1]. Deep-sea macrobenthos, which are animals that inhabit deep-sea hydrothermal vents, face high hydrostatic pressure, variable temperatures and pH, and high levels of hydrogen sulphide, methane, and heavy metals [2]. To date, the literature contains a limited number of studies on the genetics of macrobenthos. A recent report on the genome of deep-sea hydrothermal vent/cold seep mussels (*Bathymodiolus platifrons*) showed that, while most of the genes present in a related shallow-water mussel (*Modiolus philippinarum*) have been retained, many gene families have expanded in the *B. platifrons* genome. These include families that are associated with stabilising protein structures, removing toxic substances from cells, and the immune response to symbionts [3].

Gastropods represent the largest class of the phylum Mollusca, with different estimates of diversity varying from 80,000 to 150,000 species [4]. More than 218 gastropod (i.e. snail and slugs) species have been described from chemosynthetic ecosystems (i.e. solely rely on endosymbiotic bacterias for sustenance), of which more than 138 are believed to be endemic to these ecosystems [5]. Gastropods are an important component of the fauna in hydrothermal vents in terms of abundance and biomass [6]. Due to the lack of samples and fossil evidence, studies on the evolution and adaptation of deep sea chemosynthetic gastropods are very limited. The scaly-foot snail (*Chrysomallon squamiferum*) is only found in hydrothermal vents at a depth of ~3,000-metres in the Indian Ocean. There are two types of varieties without genetic differences: black (due to greigite, which is an iron sulphide mineral that covers its exterior) scaly-foot individuals from the Kairei field on the central Indian ridge and Longqi field on the Southwest Indian ridge, and white scaly-foot individuals from the Solitaire field on the Central Indian Ridge and the Carlsberg ridge on the Northwest Indian Ridge [7]. *C. squamiferum* was included in the International Union for Conservation of Nature (IUCN) Red List of Endangered Species on July 18, 2019 [8]. *Gigantopelta* spp. is a major megafaunal gastropod genus in some hydrothermal fields. The genus includes two species, *Gigantopelta chessoia* sp. nov. from East Scotia Ridge and *Gigantopelta aegis* sp. nov. from the Southwest Indian Ridge [6]. Both *Chrysomallon* and *Gigantopelta* are members

of the family Peltospiridae. They live in high-density aggregations and share several features, such as a large body size (up to > 45 mm, compared to typical sizes in other taxa of 10-15 mm, a 10-50 fold increase in body volume) and an enlarged oesophageal gland [9].

In this study, we sequenced and analysed chromosome-level genomes of the white scaly-foot snail *Chrysomallon squamiferum* (*C. squamiferum*, **Figure 1a**) from the Carlsberg ridge on the Northwest Indian Ridge and *Gigantopelta aegis* (*G. aegis*, **Figure 1a**) from the Southwest Indian Ridge. We gained insights into the evolution, gene family expansions, and adaptations of these extremophile gastropods.

Data Description

Genome assembly and annotation

The *C. squamiferum* genome was sequenced using a combination of sequencing libraries – 10X Genomics, Oxford Nanopore Technology (ONT), and Hi-C – to generate ~369.03 Gb of raw data (**Table S1**). Due to the limited sample material, *G. aegis* was sequenced from whole genome shotgun libraries (with 350 bp to 10 kb inserts on the BGISEQ-500 platform) to generate 910.08 Gb of raw data (**Table S2**). The genome of *C. squamiferum* was assembled with long ONT reads by using Canu [10] and WTDBG [11]. After polishing the genome with 10X Genomics sequencing data, a 454.58 Mb assembly (a little smaller than the estimated genome size: 495 Mb, **Figure S1**) with 6,449 contigs and an N50 of 541.32 kb was generated (**Table S3**). Next, Hi-C data were used to anchor the assembly, yielding a 16-chromosome assembly (**Figure 1b**). This effort increased the N50 size to ~20.71 Mb (**Table 1**). The 16 chromosomes cover ~80% of the whole genome, and the average length, maximal length, and minimal length of the 16 chromosomes were 22.67, 46.78, and 10.64 Mb, respectively, (**Table S4**). A Benchmarking Universal Single-Copy Orthologs (BUSCO) completeness score of 94.8% for this genome suggests that it is of good quality (**Table S5**). This is the first chromosome-level deep-sea snail genome assembly to date. An approximately 1.29 Gb (a little smaller than the estimated genome size:1.50 Gb, **Figure S1**) genome assembly of *G. aegis* with a scaffold N50 of 120.96 kb (**Table S6**) and a BUSCO completeness score of 88.4% (**Table S7**) was obtained using Platanus [12]. After masking repeat elements, we

employed homologous and *de novo* prediction methods to construct gene models for the two genomes, obtaining 28,781 *C. squamiferum* genes and 25,601 *G. aegis* genes (**Tables S8** and **S9**). The gene sets were functionally annotated using KEGG, Swiss-Prot, InterPro, and TrEMBL (**Tables S10** and **S11**).

Genome sizes and repeat contents.

The genome assembly sizes of *C. squamiferum* (~455.36 Mb) and *G. aegis* (~1.29 Gb) add to previous studies on freshwater snails (~916 Mb (*Biomphalaria glabrata*) [13] and ~440 Mb (*Pomacea canaliculate*) [14]), which suggests that there is significant genome size diversity within snails (**Figure 1c**). In the absence of ploidy effects [15, 16], differences in genome size often stem from the accumulation of various repetitive elements. A comparison of repeat elements (**Figure 1c** and **Table S12**) supports this contention. The genomes of *C. squamiferum* and *P. canaliculate* (smaller genome sizes) contained fewer repeats than *B. glabrata* and *G. aegis*, whereas *G. aegis* had more repeats than *B. glabrata* (**Figure 1d**). This suggests that snail genome sizes correlate with repeat content. Despite the similar genome sizes of *C. squamiferum* and *P. canaliculata*, their genome landscapes are distinct. For example, ~10.17% of the *C. squamiferum* genome consists of tandem repeats compared to ~2.89% in *P. canaliculata* (**Table S12**). DNA transposons and LTRs comprise ~17.73% and ~5.99% of the *C. squamiferum* genome, respectively, but only ~6.84% and ~3.53% in *P. canaliculata*. LINEs make up ~8.63% of the *P. canaliculata* genome compared to ~5.65% in *C. squamiferum*. Similarly, although the larger *G. aegis* and *B. glabrata* genomes have similar proportions of tandem repeats, *G. aegis* has a higher percentage of DNA transposons (~32.15% versus ~20.20%) and LTRs (~13.32% versus ~3.75%). LINEs make up ~23.93% of the *B. glabrata* genome compared to ~11.51% in *G. aegis*. Taken together, these data suggest that deep-sea hydrothermal vent snail genomes have more DNA transposons and LTRs and fewer LINEs than their freshwater counterparts. In particular, DNA/CMC-EnSpm, DNA/TcMar−Tc1, and DNA/DNA were the main factors that caused the differences in DNA transposon content in the four snail genomes (**Figure 1d**). We found that LINE/L2, LINE/RTE-BovB, LINE/LINE, and LINE/CR1 were much higher in fresh-water snail genomes than in deep-sea snails. Although most of the precise functions of these repeats

have not been studied in-depth, repeats have been thought to have a regulatory function in related genes that play an important role in the life cycle. Thus, we might infer that the expansion of DNA transposons and LTRs, as well as the abandonment of some LINEs, may be closely associated with adaptation to extreme environments for deep-sea snails.

Construction of phylogenetic relationships for deep-sea snails

To determine the phylogenetic relationships between deep-sea snails and other molluscs, we compared two mussels, two freshwater snails, and two shallow-water snails. The California two-spot octopus and the freshwater leech *Helobdella robusta* were used as the outgroup. We identified 26,668 gene families in the ten species examined (**Table S13**). Phylogenetic trees were constructed from 406 shared single-copy orthologs. Both ML and Bayesian methods revealed the same topology (**Figure 2a** and **Figure S2**), which is consistent with a recent study [14]. In the tree, mussels and snails are clearly separated and the two deep-sea snails are located on the same branch and are independent of other snails (although their genome sizes are quite different). We estimated that *C. squamiferum* and *G. aegis* diverged from a common ancestor approximately 66.3 million years ago (MYA). This time is consistent with the most recent 'mass extinction', at the end of the Cretaceous geological period ~66 MYA, where ~76% of species became extinct [17].

Demographic histories of the deep-sea snails

As the speciation of the two deep-sea snails may be related to geological events (*see above*), we estimated their historical effective population size (N_e) using whole-genome genetic variation. We identified ~3.51 and ~3.19 million heterozygous SNPs with nucleotide diversities of 0.0077 and 0.0025 for *C. squamiferum* and *G. aegis*, respectively. We estimated N_e changes using the pairwise sequential Markovian coalescent (PSMC) method, which can infer demography from approximately 20,000 to 1 million years ago (MYA) [18]. The effective population sizes of *C. squamiferum* and *G. aegis* – species derived from different geographical locations in the Indian Ocean – are distinct (**Figure 2b**). The demographic history of *G. aegis* decreased until ~250 KYA (thousand years ago), followed by an N_e increase, from ~50,000 to 450,000 individuals, 20,000 years ago.

Several cycles of N_e increase and decrease have been observed for *C. squamiferum*, with the effective population size recovering and stabilising at 35,000 individuals from 70 KYA onwards. Thus, although deep-sea habitats are inhabited, deep-sea snail populations are sensitive to habitat disturbances, such as major geological events. Unfortunately, the *C. squamiferum* population size has dramatically decreased recently due to deep-sea mining [8], which has made this species endangered.

Evolution of single-copy orthologous genes

The evolution and expression of single-copy orthologous genes are unique features of organisms. To explore the evolutionary rate of single-copy orthologous genes, we calculated the synonymous substitution rate (*Ka*) and nonsynonymous substitution rate (*Ks*) values of 1,324 single-copy orthologous genes shared by the two deep-sea snails, one shallow-water snail (*L. gigantea*), and two freshwater snails (*B. glabrata* and *P. canaliculate*) (**Figure 2c**, **Figure S3,** and **Table S15**). We found that the *Ka* values of the two deep-sea snails (average: 0.37 and 0.41) were higher than that of the shallow-water snail (0.35) but similar to those of two freshwater snails (0.39 and 0.41), which suggests that the genes of deep-sea and freshwater snails both evolved faster after their divergence from shallow-water snails. The *Ks* values of the deep-sea (3.34 and 3.09) and freshwater (3.19 and 3.24) snails were also similar and lower than those of the shallow-water snails (3.72). Additionally, the *Ka*/*Ks* values of the deep-sea snails (average: 0.13 and 0.15) were approximately ~20% and ~40% higher than those of the shallow-water snails (0.11); from this we could infer that deep-sea snails have experienced stronger selective pressures, possibly to allow adaptation to life in hydrothermal vents.

Expanded gene families in deep-sea snail genomes

Nervous system

Using CAFÉ [19] (*see details in methods*), we identified two significantly (p-value < 0.01) expanded gene families in the two deep-sea snail genomes compared to the freshwater and shallow-water snails. BTB/POZ domain-containing protein 6 (*BTBD6*) had 56 copies in *C. squamiferum* and 35 copies in *G. aegis*, while fewer than 5 copies were found in the four other snail species examined (**Figure 3a**). We found 17 *BTBD6*

genes on chromosome 16 of *C. squamiferum*, and these genes showed traces of tandem duplications (**Figure 3b**). In *G. aegis*, we also found several tandem gene clusters (**Figure 3b**). *HTR4* (5-hydroxytryptamine receptor 4) had 12 copies in *C. squamiferum* and 18 copies in *G. aegis*, while only one copy was found in the other snail species (**Figure 3c**). The expansions of these gene families also displayed tandem duplications (**Figure S4**). Both of these genes have roles in neuroregulation; *BTBD6*, which is an adaptor of the Cul3 ubiquitin ligase complex, is essential for neural differentiation [20], while *HTR4* modulates the release of various neurotransmitters[21]. A previous study revealed that a large unganglionated nervous system exists in *C. squamiferum* [7] (**Figure 3d**). We speculate that the expansions of *BTBD6* and *HTR4* contribute to this system by sustaining life in a deep-sea environment.

Metabolism related genes

C. squamiferum houses abundant endosymbionts in its greatly enlarged oesophageal gland, and these endosymbionts supply nutrition for its host. KEGG enrichment analysis on the 183 expanded gene families of *C. squamiferum* revealed significant enrichment for metabolic pathways (q-value < 0.0001, **Table S16**). Among these genes, nine gene families encoded enzymes in the glycolysis pathway and citrate cycle (TCA cycle). For example, isocitrate dehydrogenase (IDH), which catalyses the oxidative decarboxylation of isocitrate to produce α-ketoglutarate and CO2, expanded significantly ($p < 0.01$). The α-ketoglutarate dehydrogenase complex (OGDC) consists of three components: oxoglutarate dehydrogenase (OGDH), dihydrolipoyl succinyltransferase (DLST), and dihydrolipoyl dehydrogenase (DLD), among which OGDH expanded ($p < 0.01$, **Figure 4a**). IDH and OGDC are two rate-limiting enzymes in the TCA cycle, and related biochemical reactions are irreversible (**Figure 4b**).

Defence mechanisms

Endosymbiotic bacteria are critical for snail life in deep-sea hydrothermal vent ecosystems [22]. These bacterial taxa are largely restricted to chemosynthetic environments, with some being exclusive to vents [23]. The different genome

evolutionary processes of *C. squamiferum* and *G. aegis* may generate diverse defence mechanisms that are used to adapt to different gene evolutions.

A total of 183 expanded gene families were identified in the *C. squamiferum* genome. As expected, many of these have roles in the immune system. However, unlike the freshwater snail *B. glabrata* [13] and deep-sea mussels [3], we did not detect an expansion of the Toll-like receptor 13 (*TLR13*) gene family, but identified other expanded gene families (**Figure 4a**). For example, increased expression of thioredoxin 1 (*Txn1*; 22 copies in *C. squamiferum*) plays a pivotal role in T-cell activation in mice [24]. Although T-cell related adaptive immunity only appears in vertebrates, the existence and expansion of this gene may assist the innate immune system of *C. squamiferum*. Glutamine-fructose-6-phosphate transaminase (*GAFT*; 21 copies in *C. squamiferum*) promotes the biosynthesis of chitin [25, 26], which is one of the stable components of the crustacean shell, and provides protection against predation and infection.

We identified expanded gene families that maintain the stability of nucleic acids and proteins, such as heat shock protein 90 (Hsp90; 13 copies in *C. squamiferum*, **Figure 4a**), which protects proteins against heat stress [27]; the single-stranded DNA-binding proteins, encoded by SSB genes (19 copies in *C. squamiferum*, and 1 copy in other species, **Figure 4a)**, which are required for DNA replication, recombination, and repair processes [28]; and catalase (*CAT*, 6 copies *C. squamiferum*; **Figure 4c**), which is critical in the response against oxidative stress [29]. The elevated levels of heavy metals and sulphide, and high temperatures in hydrothermal vents are likely to greatly increase the risk of DNA damage and misfolded proteins. Thus, these expanded gene families may help these snails resist environmental stress.

We also found a special gene family, deleted in malignant brain tumours 1 (*DMBT1*), expanded (70 copies, **Figure 4a**) in the *C. squamiferum* genome. *DMBT1* can encode three glycoproteins (DMBT1 (deleted in malignant brain tumours 1 protein), SAG (salivary agglutinin), and GP340 (lung glycoprotein-340)) and belongs to the scavenger receptor cysteine-rich (SRCR) protein superfamily of the immune system [30]. This gene consists of the SRCR, CUB, and zona pellucida domains, and all 70 copies of this gene in *C. squamiferum* contain the SRCR domain, which can bind a broad range of pathogens, including cariogenic *streptococci*, *Helicobacter pylori*, and HIV [31]. However, previous

studies have shown that SRCR domains that contain proteins are commonly expressed in the shell martrix[32] and have been proven to be potentially linked to biomineralisation[33], which would be associated with the foot scales of *C. squamiferum*. Nonetheless, the expansion of this gene family will either strengthen the immune ability or help construct the scale armour of these snails.

Correspondingly, we identified the expansion of 198 gene families (containing 4,515 genes) in the *G. aegis* genome. These families were enriched in 58 KEGG pathways (*q*-value < 0.05) (**Table S17**). The majority of these pathways were associated with the immune and disease response, and included terms such as ‘infection’, ‘NOD-like receptor signalling’, ‘Tumour necrosis factor (TNF) signalling pathway’, and ‘Antigen processing and presentation’ (**Figure S5**). Surprisingly, we found 251 copies of the H-2 class II histocompatibility antigen, A-U alpha chain-like (H2-Aal) genes, which is one of the major histocompatibility complex (MHC) genes in vertebrates [34]. The existence and super expansion of this gene family in an invertebrate positions *G. aegis* as useful for the study of immune system evolution.

Discussion

Molluscs are a highly diverse group, and their high biodiversity makes them an excellent model to address topics such as biogeography, adaptability, and evolutionary processes [35]. Members of the family Peltospiridae in the gastropod clade Neomphalina are restricted to chemosynthetic ecosystems and, so far, are only known from hot vents [6]. Based on the chromosome-scale genome assembly analyses of the scaly-foot snail (*C. squamiferum*) and deep-sea snail (*G. aegi*), which both belong to the Peltospiridae family from chemosynthetic ecosystems, our results provide insight into the possible evolution and adaptation mechanisms of hydrothermal vent animals.

By constructing a phylogenetic tree, we found that snails diverged from other molluscs approximately 555.2 MYA (**Figure 2a**). These two deep-sea snails were found to be independent of other shallow-water snails around 536.6 MYA and diverged from each other approximately 66.3 MYA. This evolutionary time frame implies that the last common ancestor of all molluscs (LCAM) already lived before the infamous Cambrian Explosion (530-540 MYA), which was speculated by the palaeobiological hypothesis

[36]. It also elucidated that deep-sea gastropod lineages originated at least around 540 MYA and diverged from other gastropods in the same age of the oldest molluscs taxons, Aculifera and Conchifera [37, 38]. The deep sea gastropod lineages were also confirmed by the phylogenetic analysis of mitogenomes [39]. Further conceived by the evolutionary rate of single-copy orthologous genes, deep sea gastropod lineages have experienced stronger selective pressures than shallow-water snails (**Figure 2c**). At the end of the Cretaceous geological period, ~66 MYA, *C. squamiferum* and *G. aegis* diverged from each other and had different historical effective population sizes (*Ne*) later (**Figure 2b**). This indicated that they faced different environmental factors and selected pressures.

The transposable elements (TEs) play multiple roles in driving genome evolution in eukaryotes[40]. The genome sizes of four representative snails were quite divergent (440 Mb-1.29 Gb). The deep sea snail *G.aegi* had the largest genome (1.29Gb), with the highest percentage of DNA transposons (32.15%). Deep sea snails (*C squamiferum* and *G.aegi*) had more DNA transposons and LTRs than other snails, but fewer LINEs. LTR class has been identified as the main contributor to open chromatin regions and transcription factor binding sites[41] [42]. LINEs may be associated with the duplicability of genomic regions, which are always shared between related lineages[43]. It also indicated that the evolution of deep sea snail linages depends more on adaptive needs than on a region-specific feature shared between lineages.

Specifically, we analysed expanded gene families in deep-sea snail genomes (**Figure 4a**). They both significantly expanded the nervous system, especially BTB/POZ domain-containing protein 6 (*BTBD6*) and 5-hydroxytryptamine receptor 4 (*HTR4*), which are involved in the neuroregulation of activities, such as movement, predation, and resistance to environmental change. As for the chemosynthetic snails, they both expanded immune system-related genes. In the *C. squamiferum* genome, the expansions of thioredoxin 1 (Txn1) and Glutamine-fructose-6-phosphate transaminase (GAFT) were found. In the *G. aegi* genome, different immune and disease response genes were expanded; for example, the major histocompatibility complex (MHC) genes, H-2 class II histocompatibility antigen-like (H2-Aal). These expanded gene families were different from fresh water snails and deep sea mussels.

Interestingly, in the scaly-foot snail (*Chrysomallon squamiferum*) genome, it significantly enriched the main metabolic pathways, including the glycolysis pathway and citrate cycle (TCA cycle), expanded the single-stranded DNA-binding protein (*SSB)* family to stabilise ssDNA, heat shock protein 90 (*Hsp90*) to keep proteins folded properly, and catalase (*CAT*) to avoid free radical generation by the peroxide. These gene expansions might have provided deep sea snails with better immune reactions with symbionts, rapid nerve signal conduction, stronger metabolism, and effective resistance for adaptation to their hydrothermal vent habitat.

In particular, we found that *DMBT1* gene families that encode multiple SRCR domains expanded significantly. These genes play important roles in immune response and biomineralisation, both of which are vital for deep sea snails.

In conclusion, the genome analysis of deep-sea snails (*Chrysomallon squamiferum* and *Gigantopelta aegi*) from hydrothermal vents revealed their evolution and different molecular adaptation to extreme environments and will be a valuable resource for studying the evolution of inveterbrates.

Materials and Methods

Sample collection and DNA isolation

Chrysomallon squamiferum samples were obtained from the Daxi hydrothermal field (60.5°W 6.4°N, 2919m depth) on the Carlsberg Ridge, northwest Indian Ocean, in March 2017 during the Chinese DY38th cruise. *Gigantopelta aegis* samples were obtained from the Longqi vent field (37.8°S, 49.9°E, 2,780 m) on the southwest Indian ridge in March 2015 during the Chinese DY35th cruise. DNA was extracted from muscle samples using the cetyl trimethylammonium bromide (CTAB) method and a DNeasy blood & tissue kit (QIAGEN). DNA quality and quantity were checked using pulsed field gel electrophoresis and a Qubit Fluorometer (Thermo Scientific).

Libraries preparation and sequencing

Whole Genome Shotgun Sequencing

Four WGS libraries were prepared for sequencing: one short insert size library (350 bp) and three mate-pair large insert size libraries (2 kb, 5 kb, and 10 kb). Libraries were constructed using an MGI Easy FS DNA Library Prep Set kit (MGI, China). Paired-end reads (100 bp) and mate-pair reads (50 bp) were obtained from the BGISEQ-500 platform.

10X Genomics sequencing

To prepare the Chromium library, 1 ng of high quality DNA was denatured, spiked into reaction mix, and mixed with gel beads and emulsification oil to generate droplets within a Chromium Genome chip. Then, the rest of the steps were completed following the standard protocols for performing PCR. After PCR, the standard circularisation step for BGISEQ-500 was carried out, and DNA nanoballs (DNBs) were prepared [44]. Paired-end reads with a length of 150 bp were generated on the BGISEQ-500 platform.

Oxford Nanopore Technologies

DNA for long-read sequencing was isolated from the muscle tissues of our samples. Using 5 flow cells of the ONT chemistry for the GridION X5 sequencer

following manufacturer's protocols, we generated 39.61 Gbp of raw genome sequencing data.

Hi-C **library and sequencing**

The Hi-C library was prepared following the standard *in situ* Hi-C [45] protocol for muscle samples, using *DpnII* (NEB, Ipswich, America) as the restriction enzyme. After that, a standard circularisation step was carried out, followed by DNA nanoballs (DNB) preparation following the standard protocols of the BGISEQ-500 sequencing platform as previously described [44]. Paired-end reads with a length of 100 bp were generated on the BGISEQ-500 platform.

Genome assembly

For the genome assembly of *Chrysomallon squamiferum*, Canu (v1.7) was first used to perform corrections of ONT reads with the parameters "correctedErrorRate=0.105 corMinCoverage=0 minReadLength=1000 minOverlapLength=800". Then, wtdbg (v1.2.8) was used to assemble the genome with the parameters "--tidy-reads 3000 -k 0 -p 21 -S 4 --rescue-low-cov-edges" using corrected reads generated by Canu. Next, we made use of the sequencing reads from the 10X genomic library to carry out genome polishing using Pilon (version 1.22) with its default parameters. Quality control of Hi-C sequencing reads was first performed using the HiC-Pro pipeline [46] with the parameters "[BOWTIE2_GLOBAL_OPTIONS = --very-sensitive -L 30 --score-min L,-0.6,-0.2 --end-to-end –reorder;BOWTIE2_LOCAL_OPTIONS = --very-sensitive -L 20 --score-min L,-0.6,-0.2 --end-to-end –reorder; IGATION_SITE = GATC; MIN_FRAG_SIZE = 100; MAX_FRAG_SIZE = 100000; MIN_INSERT_SIZE = 50; MAX_INSERT_SIZE = 1500]". In total, 23,646,810 pairs of valid reads were obtained. Next, the valid Hi-C data was used to anchor the nanopore contigs onto chromosomes separately by applying the 3D-DNA [47] pipeline. The contact maps were then generated by the Juicer pipeline[48], and the boundaries for each chromosome were manually rectified by visualising the inter.hic file in Juicebox [49]. 16 chromosomes were identified by combining the linkage information from the agp file.

For the genome assembly of *Gigantopelta aegis*, we only have WGS sequencing reads because of limited DNA and tissue samples. Platanus (v1.2.4)[12] was used to perform genome assembly with WGS clean data with the parameters "assemble –k 29 –u 0.2, scaffold -l 3 -u 0.2 -v 32 -s 32 and gap_close –s 34 –k 32 –d 5000". BUSCO (v2) were used to evaluate genome assemblies with the metazoan_odb9 database.

Genome annotation

Repeat annotation

Homolog-based and *de novo* prediction methods were used to detect repeat contents. In particular, RepeatMasker (v4.0.5) [50] and RepeatProteinMasker (v 4.0.5) were used to detect transposable elements against the Repbase database[51] at the nuclear and protein levels, respectively. RepeatMasker was used again to detect species-specific transposable elements against databases generated by RepeatModeler (v1.0.8) and LTR-FINDER (v1.0.6)[52]. Moreover, Tandem Repeat Finder (v4.0.7)[53] was utilised to predict tandem repeats.

Gene annotation

We combined homology-based and *de novo* evidence to predict protein-coding genes in two genomes. For the homology-based method, we used six relative gene sets of *Aplysia californica*, *Bathymodiolus platifrons*, *Biomphalaria glabrata*, *Lottiu gigantea*, *Modiolus philippinarum*, and *Pomacea canaliculata*. First, these homologous protein sequences were aligned onto each assembled genome using TBLASTN (RRID:SCR 011822), with an *E*-value cut-off of 1×10^{-5}, and the alignment hits were linked to candidate gene loci by GenBlastA [54]. Second, we extracted genomic sequences of candidate gene regions, including 2 kb flanking sequences, and then used GeneWise (v2.2.0)[55] to determine gene models.

In the *de novo* method, we used Augustus (Augustus, RRID:SCR 008417)[56] to predict the gene models on repeat-masked genome sequences. We selected high-quality genes with intact open reading frames (ORFs) and the highest GeneWise [55] score from a homology-based gene set to train Augustus with default parameters before prediction.

Gene models with incomplete ORFs and small genes with protein-coding lengths less than 150 bp were filtered out. Finally, a BLASTP (BLASTP, RRID:SCR 001010) search of predicted genes was performed against the Swiss-Prot database (UniProt, RRID:SCR 002380) [57]. Genes with matches to Swiss-Prot proteins containing any one of the following keywords were filtered: transpose, transposon, retrotransposon, retrovirus, retrotransposon, reverse transcriptase, transposase, and retroviral. Finally, the results of the homology- and *de novo*-based gene sets were merged using GLEAN to yield a nonredundant reference gene set.

Gene function annotation

We annotated the protein-coding genes by searching against the following public databases: Swiss-Prot[58], the Kyoto Encyclopedia of Genes and Genomes (KEGG)[59], InterPro[60], and TrEMBL[58].

Phylogenetic tree reconstruction and divergence time estimation

The TreeFam tool [61] was used to identify gene families as follows: first, all the protein sequences from selected 10 representative species (*Aplysia californica*, *Octopus bimaculoides*, *Biomphalaria glabrata*, *Crassostrea gigas*, *Lottia gigantea*, *Pomacea canaliculata*, *Pinctada fucata*, *Chrysomallon squamiferum*, *Gigantopelta aegis*, and *Helobdella robusta*) were compared using blastp with the *E*-value threshold set as 1e-7. Then, alignment segments of each protein pair were concatenated using the in-house software Solar. H-scores were computed based on Bit-scores and were used to evaluate the similarity among proteins. Finally, gene families were obtained by clustering homologous gene sequences using Hcluster sg (v0.5.0).

We obtained 406 one-to-one single-copy orthology gene families based on gene family classification. Then, these gene families were extracted and aligned using guidance from amino-acid alignments created using the default parameters of the MUSCLE [62] programme. All sequence alignments were then concatenated to construct 1 super-matrix and then a phylogenetic tree was constructed under a GTR+gamma model for nucleotide sequences using ML and Bayesian methods. The same set of codon sequences were used

for phylogenetic tree construction and estimation of divergence time. The PAML mcmctree programme [63, 64] was used to determine divergence times with the approximate likelihood calculation method, and the correlated molecular clock and REV substitution model. The concatenated CDS of one-to-one orthologous genes and the phylogenomics topology were used as inputs. We used five calibration time points based on fossil records: *A. californica* - *C. gigas* (~516.3 - 558.3 million years ago (Mya)), *A. californica* - *P. canaliculata* (~310 – 496 Mya), *A. californica* - *Octopus bimaculoides* (~551 – 628 Mya), *C. gigas* - *H. robusta* (~585 – 790 Mya), and *C. gigas*– *P. fucata* (394 Mya) (http://www.timetree.org), were used as constraints in the MCMCTree estimation.

Expansion and contraction of gene families

We used CAFE (Computational Analysis of gene Family Evolution) v2.1[19] to analyse gene family expansion and contraction under the maximum likelihood framework. The gene family results from the TreeFam pipeline and the estimated divergence time between species were used as inputs. We used the parameters "-p 0.01, -r 10000, -s" to search for the birth and death parameter (λ) of gene families, calculated the probability of each gene family with observed sizes using 10,000 Monte Carlo random samplings, and reported birth and death parameters in gene families with probabilities less than 0.01.

Figure legends

Figure 1. Genome characteristics of *C. squamiferum* and *G. aegis*. a) Photos of two species. Left: *C. squamiferum*; right: *G. aegis*. **b**) Heat map of chromatin interaction relationships at a 125 kb resolution of 16 chromosomes. **c**) Genome sizes and transposable elements in *C. squamiferum*, *G. aegis*, and two representative freshwater snail genomes. **d**) Distribution of repeat sub-types of four species.

Figure 2. Phylogenetic tree, estimated N_e, and evolution of single copy orthologous genes of deep-sea snails. **a)** Phylogenetic tree of ten representative molluscs. Expanded and contracted gene families were identified using CAFE. Divergence time was estimated using mcmctree. Species names in red represent two deep-sea snails. The timescale refers to the TimeTree database. **b)** Estimated demographic histories of two deep-sea snails. The generation time set to “3” refers to the land snail [65]. The μ values are calculated in **Table S15**. **c)**. Box plot of *K*a/*K*s values for five snails.

Figure 3. Expansion of nervous system-related genes a) Phylogentic tree of *BTBD6* genes in the examined species. The grey ellipses mark different clusters of genes. **b**) Expansion pattern of *BTBD6* genes in two deep-sea snails. Grey lines represent scaffold sequences. Coloured rectangles represent *BTBD6* genes. Symbols “//” represent other genes along the scaffolds. The blue numbers: “1” represent only one gene between the tandem duplicated genes. **c**) Expansion of *HTR4* genes. The species legend in the middle was used for **a** and **c**. Gene trees of **a** and **c** were constructed using MUSCLE (v3.8.31) and FastTree (v2.1.10). **d**) Sketch map of the large unganglionated nervous system of *C. squamiferum.* The prunosus represents the nervous system. The right amplifying represents one example of neurotransmitter release.

Figure 4. **Expansion of immune, metabolism, DNA stability, and antioxidation genes**. **a**) Gene numbers of four defence-related genes (*DMBT1*, *GAFT*, *Hsp90,* and *Txn1*), three metabolism-related genes (*OGDHE1*, *OGDHE2,* and *IDH*), and the *SSB* gene. **b**) TCA cycle signal pathway. The brown ellipses represent important enzymes and the expansion

of these genes (*OGDHE1*, *OGDHE2*, and *IDH*). **c**) Expansion of the catalase (CAT) gene in selected species.

Table 1. Genome assembly and annotation of *Chrysomallon squamiferum* and *Gigantopelta aegis*.

Species	*Chrysomallon squamiferum*	*Gigantopelta aegis*
Genome size	455.36 Mb	1.29 GB
Scaffold N50	20.7 Mb	120.96 kb
Contig N50	541.32 kb	6.96 kb
Number of genes	28,781	25,601
Repeat content	30.56%	64.17%
GC content	34.48%	37.45%
Complete BUSCO	94.80%	88.40%

Data and code availability

The genome assemblies of these two genomes have been deposited in GenBank under the accession number CNP0000854. The raw sequencing reads were also uploaded to the SRA database under accession number CNP0000854.

Additional Files

Additional File 1: Supplementary Figures and Tables.docx

Author contributions

Z.S., S.L., G.F., and X.L. conceived and managed this project and amended the manuscript. X.Z., Y.Z., L.M., and I.S. performed the evolutionary analysis and wrote the manuscript. L.M., J.C., and Y.S. performed genome assembly and annotation. J.B., S.L., X.F., C.W., Z.S., H.L., N.L., and L.W. were responsible for sample collection, DNA extraction, and library construction.

Acknowledgments

We thank Dr. Yadong Zhou for providing the animal photographs. We are very grateful to the RV “Xiangyanghong 9” crew and HOV “Jiaolong” operation teams for helping us collect samples from the deep-sea hydrothermal vent field. This work was supported by the National Key R&D Programme of China (No. 2018YFC0310702).

Supplemental information

Supplemental Information can be found online.

Declaration of interests

The authors declare that they have no competing interests.

References

1. Corliss JB, Dymond J, Gordon LI, Edmond JM, Von Herzen RP, Ballard RD, et al. Submarine Thermal Springs on the Galápagos Rift. Science. 1979;203 4385:1073-83.
2. VAN DOVER CL. The ecology of deep-sea hydrothermal vents. Princeton: Princeton University Press; 2000.
3. Sun J, Zhang Y, Xu T, Zhang Y, Mu H, Zhang Y, et al. Adaptation to deep-sea chemosynthetic environments as revealed by mussel genomes. Nature ecology & evolution. 2017;1 5:0121.
4. Parkhaev PY. The Cambrian ‘basement’of gastropod evolution. Geological Society, London, Special Publications. 2007;286 1:415-21.
5. Sasaki T, Warén A, Kano Y, Okutani T and Fujikura K. Gastropods from Recent Hot Vents and Cold Seeps: Systematics, Diversity and Life Strategies. 2010.
6. Chong C, Linse K, Copley TJ and Rogers DA. The 'scaly-foot gastropod': a new genus and species of hydrothermal vent-endemic gastropod (Neomphalina: Peltospiridae) from the Indian Ocean. 2015;81 3:322-34.
7. Chen C, Copley JT, Linse K and Rogers AD. Low connectivity between ‘scaly-foot gastropod’ (Mollusca: Peltospiridae) populations at hydrothermal vents on the Southwest Indian Ridge and the Central Indian Ridge. Organisms Diversity & Evolution. 2015;15 4:663-70.
8. Sigwart JD, Chen C, Thomas EA, Allcock AL, Bohm M and Seddon M. Red Listing can protect deep-sea biodiversity. Nature Ecology and Evolution. 2019;3 8:1134-.
9. Chen C, Uematsu K, Linse K and Sigwart JD. By more ways than one: Rapid convergence at hydrothermal vents shown by 3D anatomical reconstruction of Gigantopelta (Mollusca: Neomphalina). BMC Evolutionary Biology. 2017;17 1:62.

10. Koren S, Walenz BP, Berlin K, Miller JR, Bergman NH and Phillippy AM. Canu: scalable and accurate long-read assembly via adaptive k-mer weighting and repeat separation. Genome research. 2017;27 5:722-36.
11. Ruan J and Li H. Fast and accurate long-read assembly with wtdbg2. Nature Methods. 2019:1-4.
12. Kajitani R, Toshimoto K, Noguchi H, Toyoda A, Ogura Y, Okuno M, et al. Efficient de novo assembly of highly heterozygous genomes from whole-genome shotgun short reads. Genome Res. 2014;24 8:1384-95. doi:10.1101/gr.170720.113.
13. Adema CM, Hillier LW, Jones CS, Loker ES, Knight M, Minx P, et al. Whole genome analysis of a schistosomiasis-transmitting freshwater snail. Nature communications. 2017;8:15451.
14. Liu C, Zhang Y, Ren Y, Wang H, Li S, Jiang F, et al. The genome of the golden apple snail Pomacea canaliculata provides insight into stress tolerance and invasive adaptation. GigaScience. 2018;7 9:giy101.
15. Biemont C. Genome size evolution: within-species variation in genome size. Nature Publishing Group, 2008.
16. Dehal P and Boore JL. Two rounds of whole genome duplication in the ancestral vertebrate. PLoS biology. 2005;3 10.
17. Barnosky AD, Matzke N, Tomiya S, Wogan GO, Swartz B, Quental TB, et al. Has the Earth's sixth mass extinction already arrived? Nature. 2011;471 7336:51-7.
18. Li H and Durbin R. Inference of human population history from individual whole-genome sequences. Nature. 2011;475 7357:493-6.
19. De Bie T, Cristianini N, Demuth JP and Hahn MW. CAFE: a computational tool for the study of gene family evolution. Bioinformatics. 2006;22 10:1269-71.
20. Sobieszczuk DF, Poliakov A, Xu Q and Wilkinson DG. A feedback loop mediated by degradation of an inhibitor is required to initiate neuronal differentiation. Genes & development. 2010;24 2:206-18.
21. Conductier G, Dusticier N, Lucas G, Côté F, Debonnel G, Daszuta A, et al. Adaptive changes in serotonin neurons of the raphe nuclei in 5-HT4 receptor knock-out mouse. European Journal of Neuroscience. 2006;24 4:1053-62.
22. Goffredi SK, Warén A, Orphan VJ, Van Dover CL and Vrijenhoek RC. Novel forms of structural integration between microbes and a hydrothermal vent gastropod from the Indian Ocean. Appl Environ Microbiol. 2004;70 5:3082-90.
23. Wolff T. Composition and endemism of the deep-sea hydrothermal vent fauna. CBM-Cahiers de Biologie Marine. 2005;46 2:97-104.
24. Muri J, Heer S, Matsushita M, Pohlmeier L, Tortola L, Fuhrer T, et al. The thioredoxin-1 system is essential for fueling DNA synthesis during T-cell metabolic reprogramming and proliferation. Nature communications. 2018;9 1:1851.
25. Kato N, Dasgupta R, Smartt C and Christensen B. Glucosamine: fructose-6-phosphate aminotransferase: gene characterization, chitin biosynthesis and peritrophic matrix formation in Aedes aegypti. Insect molecular biology. 2002;11 3:207-16.

26. Lagorce A, Le Berre-Anton V, Aguilar-Uscanga B, Martin-Yken H, Dagkessamanskaia A and François J. Involvement of GFA1, which encodes glutamine–fructose-6-phosphate amidotransferase, in the activation of the chitin synthesis pathway in response to cell-wall defects in Saccharomyces cerevisiae. European journal of biochemistry. 2002;269 6:1697-707.
27. Csermely P, Schnaider T, So C, Prohászka Z and Nardai G. The 90-kDa molecular chaperone family: structure, function, and clinical applications. A comprehensive review. Pharmacology & therapeutics. 1998;79 2:129-68.
28. Marceau AH. Functions of single-strand DNA-binding proteins in DNA replication, recombination, and repair. Single-Stranded DNA Binding Proteins. Springer; 2012. p. 1-21.
29. Nazıroğlu M. Molecular role of catalase on oxidative stress-induced Ca2+ signaling and TRP cation channel activation in nervous system. Journal of Receptors and Signal Transduction. 2012;32 3:134-41.
30. Hohenester E, Sasaki T and Timpl R. Crystal structure of a scavenger receptor cysteine-rich domain sheds light on an ancient superfamily. Nature Structural & Molecular Biology. 1999;6 3:228.
31. Ligtenberg AJ, Karlsson NG and Veerman EC. Deleted in malignant brain tumors-1 protein (DMBT1): a pattern recognition receptor with multiple binding sites. International journal of molecular sciences. 2010;11 12:5212-33.
32. Aguilera F, McDougall C and Degnan BM. Co-option and de novo gene evolution underlie molluscan shell diversity. Molecular biology and evolution. 2017;34 4:779-92.
33. Mann K, Edsinger-Gonzales E and Mann M. In-depth proteomic analysis of a mollusc shell: acid-soluble and acid-insoluble matrix of the limpet Lottia gigantea. Proteome science. 2012;10 1:28.
34. Benoist CO, Mathis DJ, Kanter MR, Williams II VE and McDevitt HO. Regions of allelic hypervariability in the murine Aα immune response gene. Cell. 1983;34 1:169-77.
35. Lindberg DR PW, Haszprunar G. The Mollusca: Relationships and Patterns fromTheir First Half-Billion Years. Oxford: Oxford University Press; 2004.
36. Wanninger A and Wollesen T. The evolution of molluscs. Biological Reviews. 2019;94 1:102-15.
37. Vinther J. A molecular palaeobiological perspective on aculiferan evolution. Journal of natural history. 2014;48 45-48:2805-23.
38. Vinther J. The origins of molluscs. Palaeontology. 2015;58 1:19-34.
39. Lee H, Chen W, Puillandre N, Aznarcormano L, Tsai M and Samadi S. Incorporation of deep-sea and small-sized species provides new insights into gastropods phylogeny. Molecular Phylogenetics and Evolution. 2019;135:136-47.
40. Kazazian HH. Mobile elements: drivers of genome evolution. science. 2004;303 5664:1626-32.
41. Jacques P-E, Jeyakani J and Bourque G. The majority of primate-specific regulatory sequences are derived from transposable elements. PLoS genetics. 2013;9 5.
42. Sundaram V, Cheng Y, Ma Z, Li D, Xing X, Edge P, et al. Widespread

contribution of transposable elements to the innovation of gene regulatory networks. Genome research. 2014;24 12:1963-76.

43. Janoušek V, Laukaitis CM, Yanchukov A and Karn RC. The role of retrotransposons in gene family expansions in the human and mouse genomes. Genome biology and evolution. 2016;8 9:2632-50.

44. Drmanac R, Sparks AB, Callow MJ, Halpern AL, Burns NL, Kermani BG, et al. Human genome sequencing using unchained base reads on self-assembling DNA nanoarrays. Science. 2010;327 5961:78-81. doi:10.1126/science.1181498.

45. Rao SS, Huntley MH, Durand NC, Stamenova EK, Bochkov ID, Robinson JT, et al. A 3D map of the human genome at kilobase resolution reveals principles of chromatin looping. Cell. 2014;159 7:1665-80. doi:10.1016/j.cell.2014.11.021.

46. Servant N, Varoquaux N, Lajoie BR, Viara E, Chen CJ, Vert JP, et al. HiC-Pro: an optimized and flexible pipeline for Hi-C data processing. Genome Biol. 2015;16:259. doi:10.1186/s13059-015-0831-x.

47. Dudchenko O, Batra SS, Omer AD, Nyquist SK, Hoeger M, Durand NC, et al. De novo assembly of the Aedes aegypti genome using Hi-C yields chromosome-length scaffolds. Science. 2017;356 6333:92-5. doi:10.1126/science.aal3327.

48. Durand NC, Shamim MS, Machol I, Rao SS, Huntley MH, Lander ES, et al. Juicer Provides a One-Click System for Analyzing Loop-Resolution Hi-C Experiments. Cell Syst. 2016;3 1:95-8. doi:10.1016/j.cels.2016.07.002.

49. Durand NC, Robinson JT, Shamim MS, Machol I, Mesirov JP, Lander ES, et al. Juicebox Provides a Visualization System for Hi-C Contact Maps with Unlimited Zoom. Cell Syst. 2016;3 1:99-101. doi:10.1016/j.cels.2015.07.012.

50. Tarailo-Graovac M and Chen N. Using RepeatMasker to identify repetitive elements in genomic sequences. Current Protocols in Bioinformatics. 2009:4.10. 1-4.. 4.

51. Jurka J, Kapitonov VV, Pavlicek A, Klonowski P, Kohany O and Walichiewicz J. Repbase Update, a database of eukaryotic repetitive elements. Cytogenetic and genome research. 2005;110 1-4:462-7.

52. Xu Z and Wang H. LTR_FINDER: an efficient tool for the prediction of full-length LTR retrotransposons. Nucleic acids research. 2007;35 suppl 2:W265-W8.

53. Benson G. Tandem repeats finder: a program to analyze DNA sequences. Nucleic acids research. 1999;27 2:573.

54. She R, Chu JS, Wang K, Pei J and Chen N. GenBlastA: enabling BLAST to identify homologous gene sequences. Genome Res. 2009;19 1:143-9. doi:10.1101/gr.082081.108.

55. Birney E, Clamp M and Durbin R. GeneWise and Genomewise. Genome Res. 2004;14 5:988-95. doi:10.1101/gr.1865504.

56. Keller O, Kollmar M, Stanke M and Waack S. A novel hybrid gene prediction method employing protein multiple sequence alignments. Bioinformatics. 2011;27 6:757-63. doi:10.1093/bioinformatics/btr010.

57. UniProt C. UniProt: a worldwide hub of protein knowledge. Nucleic Acids Res. 2019;47 D1:D506-D15. doi:10.1093/nar/gky1049.

58. Boeckmann B, Bairoch A, Apweiler R, Blatter M-C, Estreicher A, Gasteiger E, et al. The SWISS-PROT protein knowledgebase and its supplement TrEMBL in 2003. Nucleic acids research. 2003;31 1:365-70.

59. Kanehisa M and Goto S. KEGG: kyoto encyclopedia of genes and genomes. Nucleic acids research. 2000;28 1:27-30.
60. Apweiler R, Attwood TK, Bairoch A, Bateman A, Birney E, Biswas M, et al. The InterPro database, an integrated documentation resource for protein families, domains and functional sites. Nucleic acids research. 2001;29 1:37-40.
61. Li H, Coghlan A, Ruan J, Coin LJ, Heriche JK, Osmotherly L, et al. TreeFam: a curated database of phylogenetic trees of animal gene families. Nucleic Acids Res. 2006;34 Database issue:D572-80. doi:10.1093/nar/gkj118.
62. Edgar RC. MUSCLE: multiple sequence alignment with high accuracy and high throughput. Nucleic Acids Res. 2004;32 5:1792-7. doi:10.1093/nar/gkh340.
63. Yang Z. PAML 4: phylogenetic analysis by maximum likelihood. Mol Biol Evol. 2007;24 8:1586-91. doi:10.1093/molbev/msm088.
64. Yang Z and Rannala B. Bayesian estimation of species divergence times under a molecular clock using multiple fossil calibrations with soft bounds. Mol Biol Evol. 2006;23 1:212-26. doi:10.1093/molbev/msj024.
65. Schilthuizen M. Rapid, habitat-related evolution of land snail colour morphs on reclaimed land. Heredity. 2013;110 3:247-52.

Figure 1

Click here to download Figure Figure 1.pdf

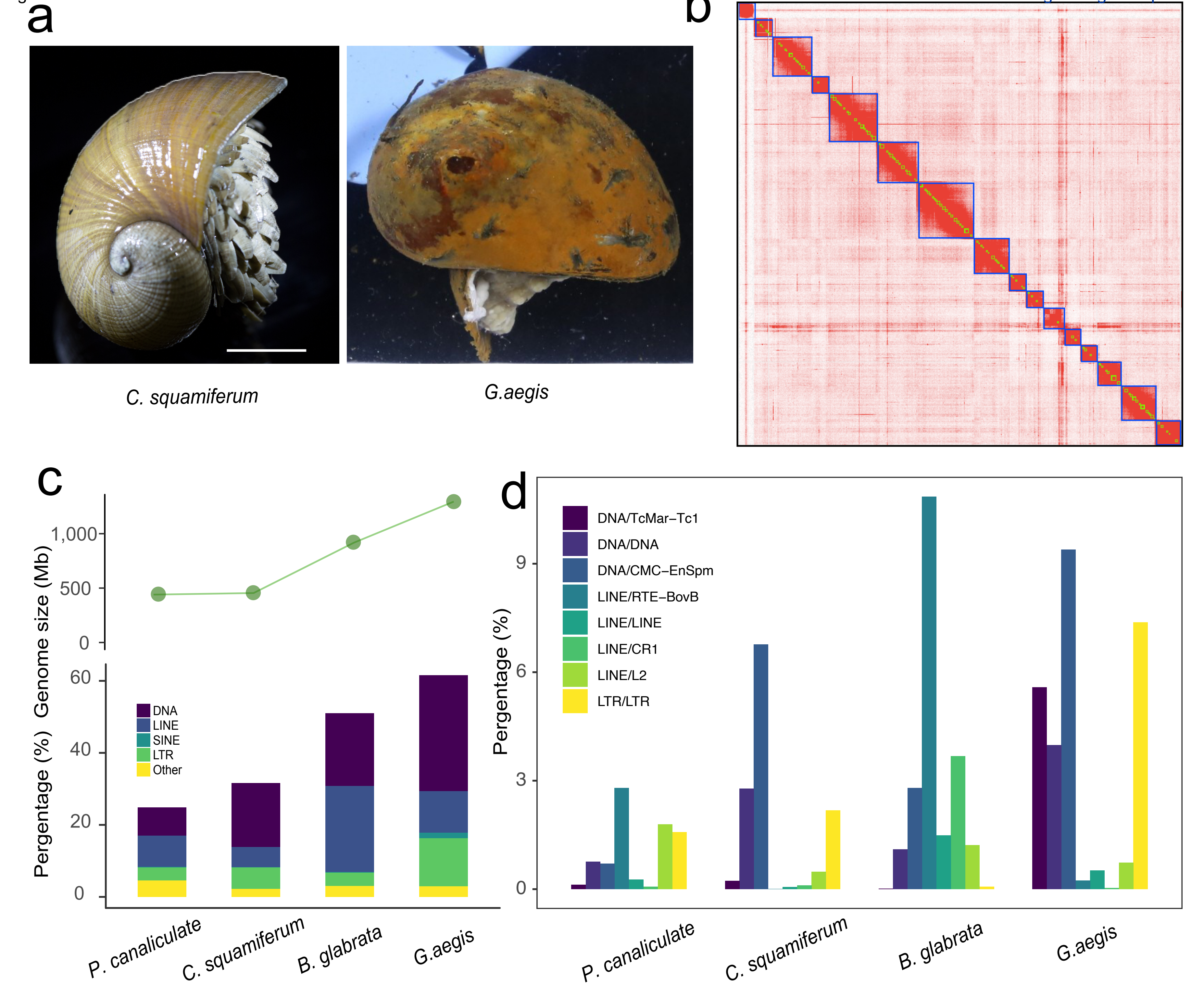

Figure 2

Click here to download Figure Figure 2.pdf

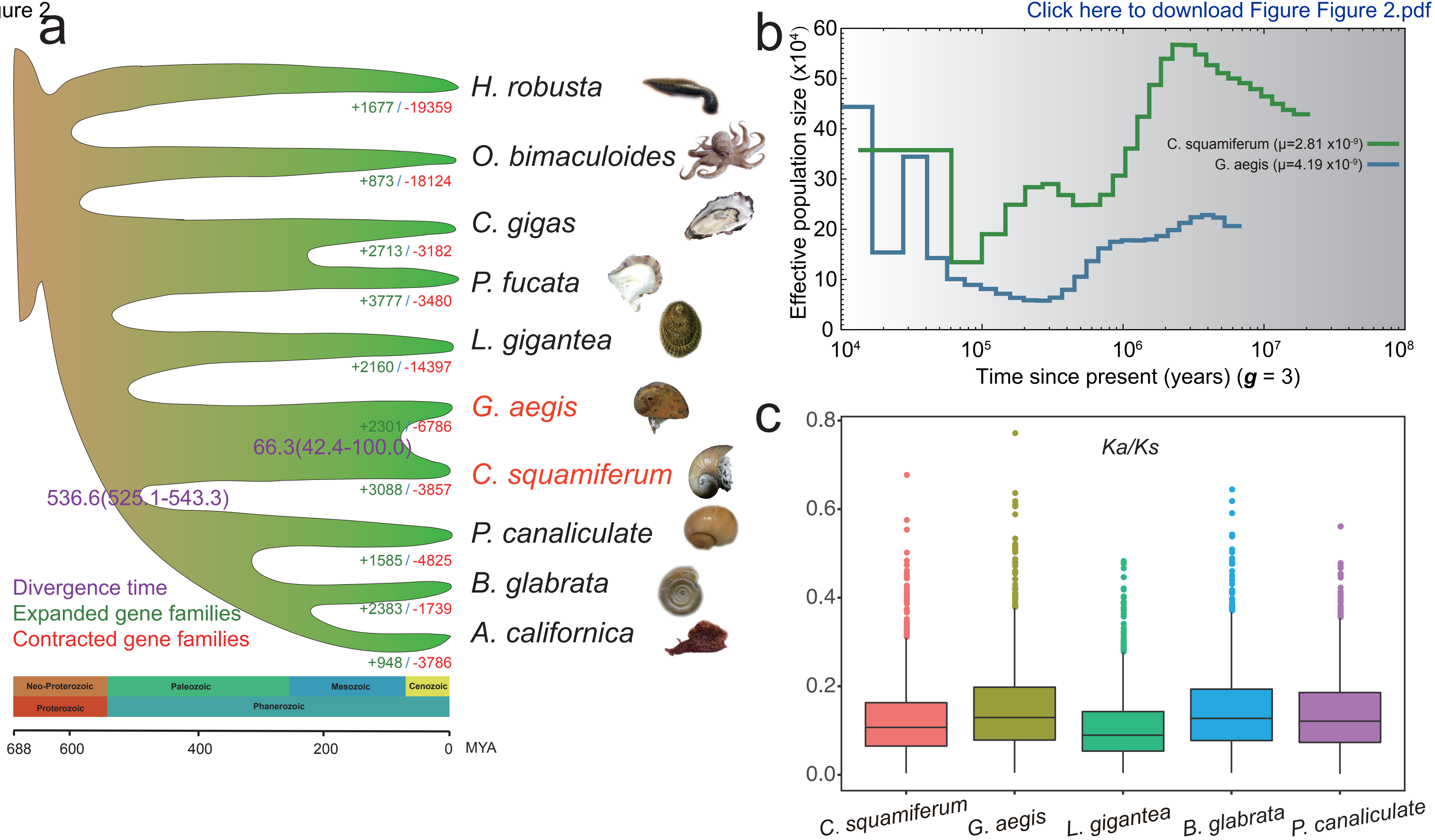

Figure 3 Click here to download Figure Figure 3.pdf

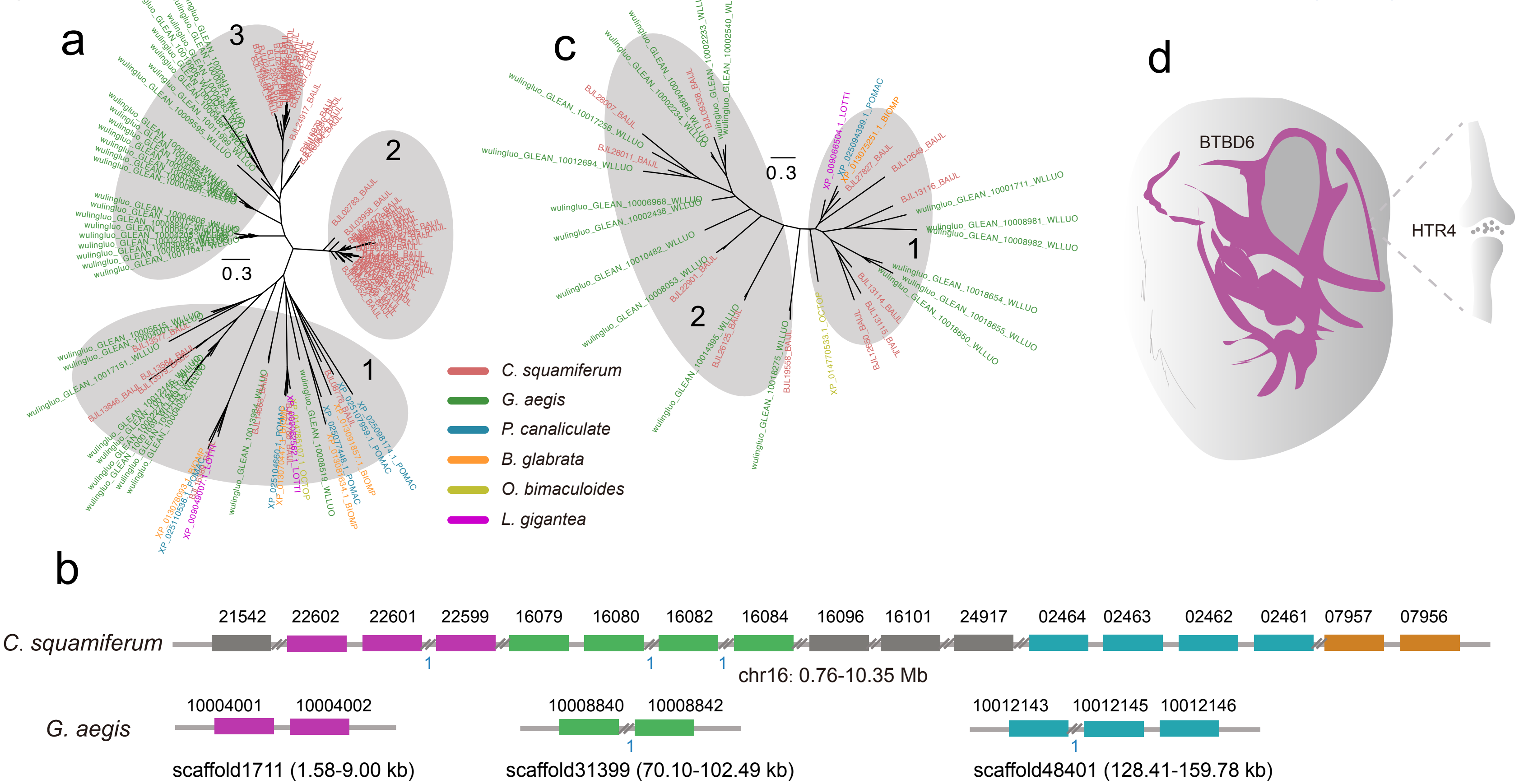

Figure 4

Click here to download Figure Figure 4.pdf

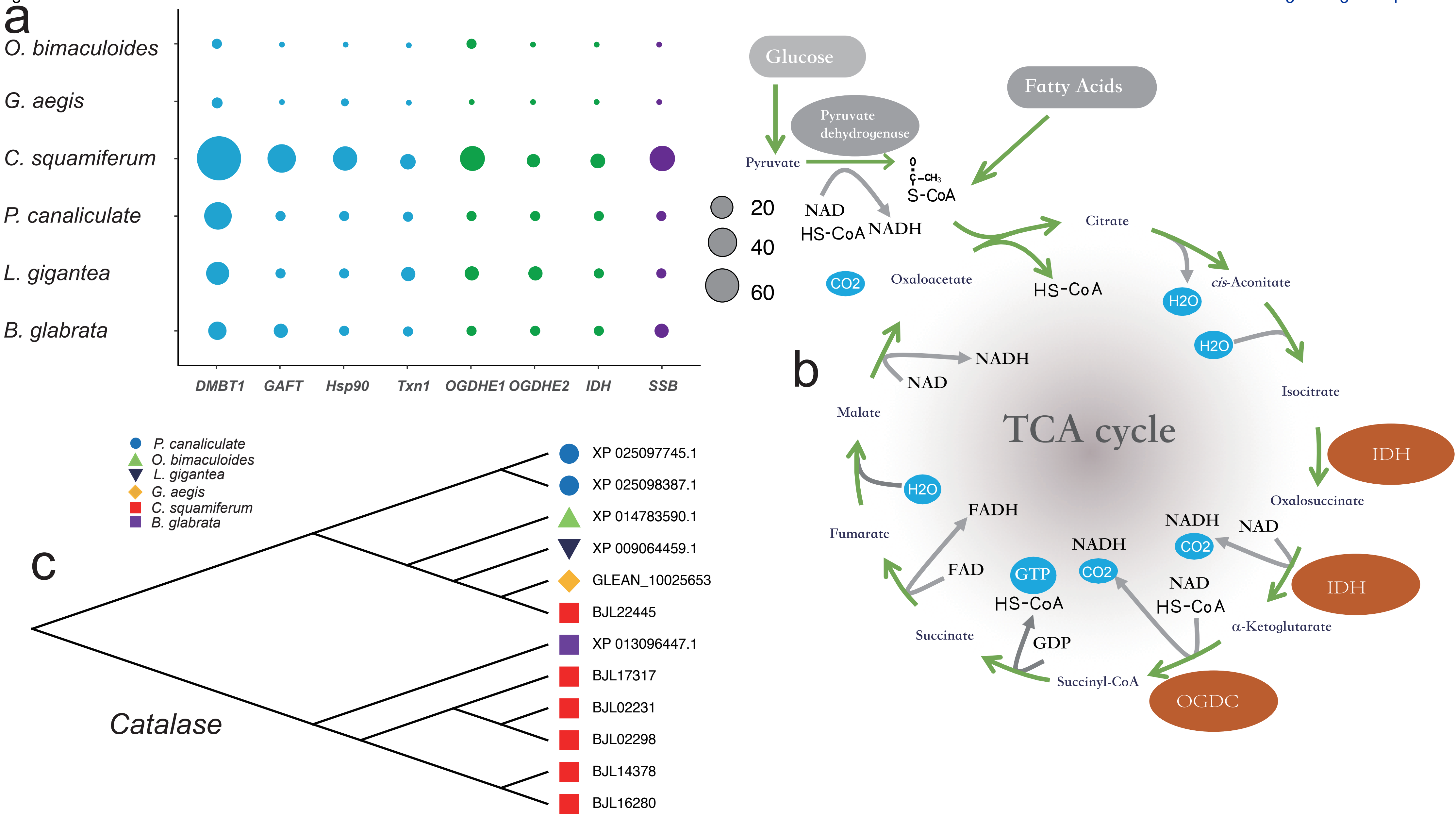

Click here to access/download
Supplementary Material
Supplementary Figures and Tables.docx
