# Supplementary material for: Genome sequencing of deep-sea hydrothermal vent snails reveals adaptions to extreme environments
Source: Gigascience. 2020 Dec 15;9(12):giaa139. doi: 10.1093/gigascience/giaa139 (PMC7736800; doi:10.1093/gigascience/giaa139)
Supplement: giaa139_GIGA-D-20-00187_Revision_1 [file giaa139_giga-d-20-00187_revision_1.pdf]

GigaScience

Genome sequencing of deep-sea hydrothermal vent snails reveals adaptations to extreme environments

--Manuscript Draft--

**Manuscript Number:**	GIGA-D-20-00187R1
**Full Title:**	Genome sequencing of deep-sea hydrothermal vent snails reveals adaptations to extreme environments
**Article Type:**	Data Note
**Funding Information:**	National Key R&D Programme of China (No. 2018YFC0310702) — Dr. Xiang Zeng
**Abstract:**	Background<br><br>The scaly-foot snail (Chrysomallon squamiferum) is highly adapted to deep-sea hydrothermal vents and drew people’s interest once it was found. However, the limited information on its genome impeded related research and understanding of its adaptation to deep-sea hydrothermal vents.<br><br>Findings<br><br>Here, we report the whole-genome sequencing and assembly of the scaly-foot snail and another snail (Gigantopelta aegis), which inhabits similar environments. Using ONT, 10X genomic, and Hi-C technologies, we obtained a chromosome-level genome of C. squamiferum with an N50 size of 20.71 Mb. By constructing a phylogenetic tree, we found that these two deep-sea snails were independent of other snails, and their divergence from each other occurred approximately 66.3 million years ago. Comparative genomic analysis showed that different snails have diverse genome sizes and repeat contents. Deep-sea snails have more DNA transposons and LTRs, but fewer LINEs, than other snails. Gene family analysis revealed that deep-sea snails experienced stronger selective pressures than freshwater snails, and the nervous system, immune system, metabolism, DNA stability, antioxidation and biomineralization-related gene families were significantly expanded in scaly-foot snails. We also found 251 class II histocompatibility antigen H2-Aal, which uniquely exist in the Gigantopelta aegis genome, which is important for investigating the evolution of MHC genes.<br><br>Conclusion<br><br>Our study provides new insights into deep-sea snail genomes and valuable resources for further studies.
**Corresponding Author:**	Yaolei Zhang<br>BGI<br>Qingdao, CHINA
**Corresponding Author Secondary Information:**	
**Corresponding Author's Institution:**	BGI
**Corresponding Author's Secondary Institution:**	
**First Author:**	Yaolei Zhang
**First Author Secondary Information:**	
**Order of Authors:**	Yaolei Zhang<br>Xiang Zeng<br>Lingfeng Meng<br>Guangyi Fan

Powered by Editorial Manager® and ProduXion Manager® from Aries Systems Corporation

	Jie Bai
	Jianwei Chen
	Yue Song
	Inge Seim
	Congyan Wang
	Zenghua Shao
	Nanxi Liu
	Haorong Lu
	Xiaoteng Fu
	Liping Wang
	Xin Liu
	Shanshan Liu
	Zongze Shao
**Order of Authors Secondary Information:**	
**Response to Reviewers:**	Response to editors and reviewers:<br><br>Dear editor and reviewers:<br>On behalf of all the coauthors, we would like to thank you very much and all the reviewers for the time spent to assess our manuscript (GIGA-D-20-00187) and for your relevant remarks and suggestions that allow to improve the quality of this manuscript. We have checked the manuscript carefully and revised it according to the comments.<br><br>Sincerely,<br>Xiang Zeng, Yaolei Zhang<br><br>Reviewer reports:<br>Reviewer #1: The Data Note by Zeng et al. reported two genome assemblies of deep sea gastropods, Chrysomallon squamiferum and Gigantopelta aegis.<br>I would ask the authors for additional informations about assembling process and references of data sources in order to guarantee the quality of the data and analyses. I also found there are many ambiguous expressions in the present manuscript, making it unclear how the genome resources can contribute to understand biology of these animals.<br>Please find my specific comments and concerns below, which need to be addressed.<br>Response: Thank you very much for your thoughtful and helpful suggestions. We have revised our manuscript according to your comments. Please see that.<br><br>Background<br>The authors should mention the fact that the Chrysomallon squamiferum genome has been published by Sun et al. (Nat Commun 11, 1657, 2020) somewhere in the Background section.<br>I would suggest the authors to explain that they analyzed the genome of "white scaly foot individual" while Sun et al. sequenced "black" one, to emphasize the uniqueness of this study.<br>Response: Thank you very much for this helpful suggestion. We have added descriptions as you suggested. Please see lines 91-93 “And the whole genome of black scaly-foot snail was reported recently, which highlighted its evolutionary mechanisms of biomineralised armour [9]” and 109-110: “In this study, we sequenced and assembled genomes of the white scaly-foot snail (Figure 1a), which is different from the published black individual”.<br><br>Line 93<br>Remove "sp. nov.". This abbreviation is used when new species is named.<br>Response: Thank you. We removed this abbreviation. See lines 94-96: “The genus includes two species, Gigantopelta chessoia from East Scotia Ridge and Gigantopelta

aegis from the Southwest Indian Ridge [6]. Both Chrysomallon and Gigantopelta are members of the family Peltospiridae.”

Data description
Line 110
Was the insert size 350bp (main text) or 300bp (Table S2)?
Response: Thank you very much. We modified it as “350 bp” in Table S2.

Line 121-122
As mentioned above, the C. squamiferum genome has been published. Therefore this sentence needs to be removed.
Response: Thank you. We delete this sentence.

Line 156-160
These sentences do not make sense to me. Why despite "precise functions of these repeats have not been studied," the authors can infer the composition of repeat elements "may be closely associated with adaptation to extreme environment"? Please describe more specifically by mentioning some references that support this idea.
Response: Thank you and sorry for confusing you. We added more description and relevant references about the importance of transposons/repeats. Please see lines: 156-161 “Although most of the precise functions of these repeats have not been studied in depth, repeats have been thought to have a regulatory function in related genes that play an important role in the life cycle and can introduce great genome flexibility [18]. And in the mammalian genome, transposons were described to be redundant enhancers that regulate their target genes which are higher or tissue specially expressed, indicating the importance of transposons”

Lines 173-175
It seems the authors assumed the split of C. squamiferum and G. aegis was related to the mass extinction event around 66 MYA. However, estimated divergence time is considerably ambiguous (42.4-100 MYA, Fig 1a), making the idea less reliable.
Response: Thank you. We agree with you and delete this description.

Deleted sentences: “This time is consistent with the most recent ‘mass extinction’, at the end of the Cretaceous geological period ~66 MYA, where ~76% of species became extinct” for your reference.

Lines 178-180
This sentence is difficult to understand. Speciation and demographic histories of each species are different topics.
Response: Thank you. We also delete this sentence.

Deleted sentences: “As the speciation of the two deep-sea snails may be related to geological events (see above)” for your reference.

Lines 190-191
This sentence is not clear. Please describe what "major geological events" affected the population sizes, with references describing the geological events.

An: Thank you. We added one example of geological events and relevant reference. Please see lines 194-197: “For example, the Cretaceous/Paleogene (K/Pg) extinction event caused extinction of three-quarters of species on earth and affected population dynamics approximately 66 million years ago, when an asteroid impact caused global environmental devastation [21, 22]”

Lines 191-193
The recent decreased population size was reported by ref[8] and was not related to this study (Fig2b). Then, this sentence may be put in the Background section.
An: Thank you. We deleted this sentence.

Deleted sentences: ” Unfortunately, the C. squamiferum population size has dramatically decreased recently due to deep-sea mining [8], which has made this species endangered” just for your reference

Powered by Editorial Manager® and ProduXion Manager® from Aries Systems Corporation

Lines 196-210
Comparisons of Ks and Ka values among snail species should be tested statistically. In the figures 2C and S3 it is not clear whether these values are significantly different.
Response: Thank you. We have added statistical test (Mann-Whitney U test) for Ka/Ks comparisons. Please see lines 204-213: "We found that the Ka values of the two deep-sea snails (average: 0.37 and 0.41) were higher (Mann-Whitney U test, p-value<0.001) than that of the shallow-water limpet (0.35) but similar to those of two freshwater snails (0.39 and 0.41), which suggests that the genes of deep-sea and freshwater snails both evolved faster after their divergence from shallow-water limpet . The Ks values of the deep-sea (3.34 and 3.09) and freshwater (3.19 and 3.24) snails were also similar and lower (Mann-Whitney U test, p-value<0.001) than those of the shallow-water limpet (3.72). Additionally, the Ka/Ks values of the deep-sea snails (average: 0.13 and 0.15) were approximately ~20% and ~40% higher (Mann-Whitney U test, p-value<0.001) than those of the shallow-water limpet (0.11)".

Discussion
Lines 309-310
I have no idea what the "infamous Cambrian Explosion" means. Please explain the authors' idea more in detail.
Response: Thank you. It was modified to "Cambrian Explosion". It was a clerical error. See line 319.

Lines 328-329
Please describe what are "adaptive needs" and "region-specific features" specifically.
Response: Thank you. We deleted this sentence.

Materials and Methods
Line 370
350bp or 300bp?
Response: It is "350 bp" actually for BGISEQ.

Lines 403-405
Based on the description, the 10X Chromium reads were used only for polishing, not for scaffolding. On the other hand, there are stats of scaffolds before Hi-C scaffolding in Table S3.
Response: Yes, the 10X Chromium reads were used only for polishing, not for scaffolding.

My questions are;
i) Were the scaffolds in Table S3 generated using 10X Chromium reads?
ii) If so, the scaffolds were improved very little (sequences are reduced from 6449 to 6444), indicating there was problem in 10X Chromium sequencing. How the authors interpreted the results?
Response: Thank you. The 6,444 scaffolds (also 6,444 contigs) were generated using Oxford Nanopore reads. We then used 10X Chromium reads to polish (error correction) 6,444 scaffolds with software Pilon. Pilon introduced 5 bp gaps so the contig number became 6,449 while the scaffold number was still 6,444.

Line 436
"Lottia"
Response: Thank you. We corrected this word.

Line 454
Describe a reference for GLEAN.
Response: Thank you. We added a reference for GLEAN. Please see line 465.

Lines 464-467
Describe sources or references for these genomic data.
Response: Thank you. We added sources for these genomic data. Please see lines 474-479: " all the protein sequences from selected 10 representative species (8 species including Aplysia californica (GCF_000002075.1), Octopus bimaculoides (GCF_001194135.1), Biomphalaria glabrata (GCF_000457365.1), Crassostrea gigas (GCF_000297895.1), Lottia gigantea (GCF_000327385.1), Pomacea canaliculate (GCF_003073045.1), Pinctada fucata (GCA_002216045.1), Helobdella robusta

Powered by Editorial Manager® and ProduXion Manager® from Aries Systems Corporation

(GCF_000326865.1) from NCBI database, C. squamiferum and G. aegis from this research) were compared using blastp with the E-value threshold set as 1e-7."

Lines 484-487
Describe references of these fossil records rather than summary database (Timetree.org) so that readers can refer the original data.
Response: Thank you very much and we can't agree with you more. However, each of the time point between two species refers a lot of references and TimeTree database summarized all of these references to estimate one divergence time with a confidence interval. For example, the divergence time between Aplysia californica and Crassostrea gigas was estimated to be 537 MYA with a confidence interval of 516.3-558.3 MYA based on 11 references. We used this confidence interval time to calibrate our estimation. This is a common method used frequently in nowadays genome research. So here we cite TimeTree database (Timetree.org) for reference which includes many references. However, if you think list all the references is a must, we are pleased to do this.

Methods of SNP identification and PSMC (lines 177-193) were not described.
Response: Thank you. We have added methods of SNP identification and PSMC. Please see lines 503-516: "SNP calling and estimation of history population sizes About 50X clean WGS reads were mapped to genomes of C. squamiferum and G. aegis using BWA mem (v0.7.12-r1039) [73] with default parameters respecitvely. Then SAMtools (v0.1.19-44428cd) [74] and "SortSam.jar" in the picard package (v1.54) was used to convert and sort BAM files. Local realignment was again carried out using RealignerTargetCreator and IndelRealigner in GATK (v3.6) [75] with default parameters. SNPs were identified using HaplotypeCaller and filtered using VariantFiltration with parameter "-filter-expression "QD < 2.0 || MQ < 40.0 || ReadPosRankSum < -8.0 || FS > 60.0" --filter-name LowQualFilter --genotype-filter-expression "DP < 5.0" --genotype-filter-name lt_5". Estimatation of history population sizes were carried out using PSMC (v0.6.5-r67) [76]. Firstly, diploid genome references were constructed using samtools and bcftools call with "samtools mpileup -C50" and "vcfutils.pl vcf2fq -d 20 -D 100". Secondly, the demographic history was inferred using PSMC with parameters '-N25 -t15 -r5 -p 4+25*2+4+6' [77]."

Fig 1a
Add the size of the scale for C. squamiferum. No scale is indicated for G.aegis.
Response: Thank you. Scale was added in Fig 1a. Also, we added "Scale bar = 1cm" in Fig 1 legend.
Fig 2a
This cartoon is too ambiguous and not suitable for scientific paper. The molecular phylogeny should be clearly shown by solid lines.
Response: Thank you. We have updated Fig. 2a. Please see that.

Fig.3d
This figure is not very informative for readers. The authors may want to draw molecular phylogeny trees for BTBD6 and HTR4.
Response: Thank you. We also deleted Fig.3d.

Reviewer #2: The manuscript entitled "Genome sequencing of deep-sea hydrothermal vent snails reveals adaptations to extreme environments" presents a nice description of a good genome assembly (16 chromosomes representing ~80% of the genome) of the scaly foot snail (Chrysomallon squamiferum) and compare it to genomes of other molluscan species. Overall the paper is well written and presents a nice view of some unique adaptations by this deep-sea mollusc. One concern that I had is throughout the manuscript (starting at line 164 and onward) the authors describe comparing two mussels, two freshwater snails and two shallow-water snails to their genomes. However, these other molluscan species include C. gigas and P. fucata…which are both oysters and not mussels, and while two of the other molluscs included in the tree are in Gastropoda and considered snails, Lottia gigantea is a limpet and Aplysia californica is a sea slug. I would encourage the authors to describe all of these species more accurately, i.e., as limpet and sea slug, because these are very different from what people commonly think of when they hear "snail", represented by the more traditional Pomacea and Biomphalaria. Referring to all the "snails" as gastropods would be a more suitable term that captures the true diversity of this large group. But

when discussing individual species, I would prefer to see the more accurate descriptions because limpets and sea slugs are different from traditional snails, and will have unique adaptations of their own related to their unique characteristics. Overall, the authors give a good general description of the results and present a reasonable discussion about some of the potential adaptations that they observed in the genome. One minor point - thioredoxins are much more likely play a role in repairing proteins that have been altered by oxidation (Lines 255-256), so to limit this expansion to innate immunity leaves out a lot of other possibilities. My other question was regarding the source of the genomic DNA. The authors describe using muscle samples for isolating DNA, but it is not clear if DNA from one individual was used for all sequencing or if pooling occurred?
Response: Thank you very much for your approval and your thoughtful advices. We have updated our descriptions based on your suggestions. Please see below response.

1) About the scientific name (line 164 and onward), we modified this part as "we compared them with two shallow-water bivalves (P.fucata and C.gigas) and four shallow-water gastropods, including two fresh-water snails (B. glabrata and P. canaliculate), one limpet (B. glabrata) and one sea slug (A. californica). The California two-spot octopus (O. bimaculoides) and the freshwater leech (H.robusta) were used as the outgroup (Figure 2a) (lines 167-171)."
2) About thioredoxins, we added the description of thioredoxin as redox proteins and references. Please lines 260-265: "For example, increased expression of thioredoxin 1 (Txn1; 22 copies in C. squamiferum) was identified. Thioredoxin 1 (Txn1), a redox protein, is important in regulation of cellular redox homeostasis and anti-apoptotic functions. Txn1 stimulates cell proliferation and cell cycle progression, induces hypoxia-inducible factor-1α (HIF-1α) and angiogenesis, and alters the balance between the matrix metalloproteinases and their tissue inhibitors [29, 30]"
3) About isolating DNA, all DNA was isolated from one individual and we add clear description about this in lines 374-375: "DNA was extracted from muscle sample of one individual using the cetyl trimethylammonium bromide (CTAB) method and a DNeasy blood & tissue kit (QIAGEN)."

Reviewer #3: The manuscript of Zeng et al seems to describe a well-put together genome for one species of deep-sea snail, with an additional 'draft' genome for another species. It is clear and well-written, with most of the methods described sufficiently. My main criticism is that I found some of the discussions regarding the adaptative significance and/or putative "function" of various TE content and gene-family expansion results quite speculative, given that the comparative results are often observational with no hypothesis testing or statistical framework. That may well be beyond the remit of the paper, but the language could be more careful in places to reflect the putative nature of any hypothesised effects. Nonetheless I have no doubt that the genomes themselves will be useful additions to the community for future work on mollusc and animal evolution.
Response: Thank you very much for your approval of our manuscript and your helpful criticism. Yes, you are quite right. Here we did not show any experiments results to valid our hypothesis or speculation because this is a data description paper, mainly focusing on observational data. More investigations including both in-depth analysis and experiments based on these two genomes and these findings will be carried out in the future to verify function of important genes or TEs or conserved no-coding regions. These are important and interesting issues and must be done then. Thank you again.

Minor comments:
- Typo line 38: "impedes"
Response: Thank you. This word was modified

- Line 158: is there a reference or two for this? I would assume that most TEs are simply selfish genetic elements that do not serve a "function" per se but exist only for their own purpose, i.e. to copy themselves independently of the host genome
- Line 158-160: but most TE content differences are probably driven by stochastic forces (i.e. drift) rather than deterministic forces such as adaptation, and here we have only 2 data points. The language used for this statement is careful, but I wonder if it is

	too far to extrapolate that some differences in TE content may be adaptive<br>Response: Thank you very much for your helpful thoughts. We added more descriptions and references to support “TEs are functional” to make it clear. Please see lines 156-161: “Although most of the precise functions of these repeats have not been studied in depth, repeats have been thought to have a regulatory function in related genes that play an important role in the life cycle and can introduce great genome flexibility[18]. And in the mammalian genome, transposons were described to be redundant enhancers that regulate their target genes which are higher or tissue specially expressed, indicating the importance of transposon”.<br><br>- Line 196: I don't know what the authors mean by this statement<br>Response: Thank you. We deleted the sentence.<br><br>Deleted sentence “The evolution and expression of single-copy orthologous genes are unique features of organisms.” for your reference.<br><br>- Section on Ka/Ks values: there is no impression given about the statistical significance of the differences observed between Ka/Ks in any given lineage, or what the distribution of error looks like for these point estimates. Perhaps a more refined PAML analysis could resolve this? It is also not written how Ka/Ks values were calculated<br>Response: Thank you. We have added statistical test (Mann-Whitney U test) for Ka/Ks comparisons. Please see lines 204-213: “We found that the Ka values of the two deep-sea snails (average: 0.37 and 0.41) were higher (Mann-Whitney U test, p-value<0.001) than that of the shallow-water limpet (0.35) but similar to those of two freshwater snails (0.39 and 0.41), which suggests that the genes of deep-sea and freshwater snails both evolved faster after their divergence from shallow-water limpet . The Ks values of the deep-sea (3.34 and 3.09) and fresh-water (3.19 and 3.24) snails were also similar and lower (Mann-Whitney U test, p-value<0.001) than those of the shallow-water limpet (3.72). Additionally, the Ka/Ks values of the deep-sea snails (average: 0.13 and 0.15) were approximately ~20% and ~40% higher (Mann-Whitney U test, p-value<0.001) than those of the shallow-water limpet (0.11).<br><br>And the Ka/Ks values were calculated actually using codeml in PAML package. We have added this information to lines 200-204: “To explore the evolutionary rate of single-copy orthologous genes, we calculated the synonymous substitution rate (Ka) and nonsynonymous substitution rate (Ks) values of 1,324 single-copy orthologous genes shared by the two deep-sea snails, one shallow-water limpet (L. gigantea), and two freshwater snails (B. glabrata and P. canaliculate) using Codeml in PAML package[23].”<br><br>- Typo line 214: CAFE not CAFÉ<br>Response: Thank you. It was modified.<br>- Line 329: "region-specific feature shared between lineages" - not sure what is meant by this?<br>Response: Thank you. We deleted this confusing sentence.<br>- Line 350: it seems speculative - surely both immune response and biomineralization are "vital" for all snails, not particularly deep-sea ones?<br>Response: Thank you. We modified it and specified it in C. squamiferum which is a chemosynthetic snail species depending on endosymbionts. See lines 360-362 “In particular, we found that DMBT1 gene families that encode multiple SRCR domains expanded significantly in C. squamiferum. These genes play important roles in immune response and biomineralization, both of which are vital for deep sea chemosynthetic snail”.<br><br>- Line 454: reference for GLEAN is missing<br>Response: Thank you. We added this reference (65). See line 465<br><br>- Line 469: references for Solar and Hcluster are missing, and what is a H-score?<br>Response: Thank you. Solar, Hcluster_sg and H-score are tools and concept of TreeFam tools. We added reference for Solar and Hcluster_sg. H-score means hcluster score. Details can be found from TreeFam tools.

Powered by Editorial Manager® and ProduXion Manager® from Aries Systems Corporation

	- Figure 2a: it's a weird looking tree that, in fact, looks a bit like a snail itself! Are the widths of the blobs representative of the error around the divergence times or topological support?<br>Response: Thank you and sorry for confusing you. We updated Fig.2a. Please see that.
**Additional Information:**	
**Question**	**Response**
Are you submitting this manuscript to a special series or article collection?	No
**Experimental design and statistics**<br><br>Full details of the experimental design and statistical methods used should be given in the Methods section, as detailed in our Minimum Standards Reporting Checklist. Information essential to interpreting the data presented should be made available in the figure legends.<br><br>Have you included all the information requested in your manuscript?	Yes
**Resources**<br><br>A description of all resources used, including antibodies, cell lines, animals and software tools, with enough information to allow them to be uniquely identified, should be included in the Methods section. Authors are strongly encouraged to cite Research Resource Identifiers (RRIDs) for antibodies, model organisms and tools, where possible.<br><br>Have you included the information requested as detailed in our Minimum Standards Reporting Checklist?	Yes
**Availability of data and materials**<br><br>All datasets and code on which the conclusions of the paper rely must be either included in your submission or deposited in publicly available repositories (where available and ethically appropriate), referencing such data using	Yes

a unique identifier in the references and in the “Availability of Data and Materials” section of your manuscript.<br><br>Have you have met the above requirement as detailed in our Minimum Standards Reporting Checklist?	

Powered by Editorial Manager® and ProduXion Manager® from Aries Systems Corporation

Manuscript Click here to access/download;Manuscript;Manuscript_Revison_2-2.docx

Genome sequencing of deep-sea hydrothermal vent snails reveals adaptions to extreme environments

Xiang Zeng[1†], Yaolei Zhang[2,3,4†], Lingfeng Meng[2†], Guangyi Fan[2,3,6], Jie Bai[3], Jianwei Chen[2], Yue Song[2] , Inge Seim[7,8], Congyan Wang[2], Zenghua Shao[2], Nanxi Liu[3], Haorong Lu[3], Xiaoteng Fu[1], Liping Wang[1],Xin Liu[2,3,5], Shanshan Liu[2*], Zongze Shao[1*]

[1]Key Laboratory of Marine Biogenetic Resources, Third Institute of Oceanography, Ministry of Natural Resources, No.178 Daxue Road, Xiamen 361005, China.

[2]BGI-Qingdao, BGI-Shenzhen, Qingdao 266555, China

[3]BGI-Shenzhen, Shenzhen, 518083, China

[4]Department of Biotechnology and Biomedicine, Technical University of Denmark, Lyngby, 2800, Denmark

[5]China National GeneBank, BGI-Shenzhen, Shenzhen 518120, China

[6]State Key Laboratory of Agricultural Genomics, BGI-Shenzhen, Shenzhen 518083, China.

[7]Integrative Biology Laboratory, College of Life Sciences, Nanjing Normal University, Nanjing, 210046, China

[8]Comparative and Endocrine Biology Laboratory, Translational Research Institute-Institute of Health and Biomedical Innovation, School of Biomedical Sciences, Queensland University of Technology, Woolloongabba, 4102, Australia

[†] These authors contributed equally.

*Correspondence: shaozz@163.com (Z.S.); liushanshan@genomics.cn (S.L.)

ORCIDs:

Yaolei Zhang: 0000-0003-2131-0254

Xiang Zeng: 0000-0001-6662-4551

Lingfeng Meng: 0000-0001-9051-0992

Guangyi Fan: 0000-0001-7365-1590

Jie Bai: 0000-0003-3839-5450

Jianwei Chen: 0000-0003-2332-4609

Yue Song: 0000-0002-2974-6442

Inge Seim: 0000-0001-8594-7217

Congyan Wang: 0000-0002-0734-5461
Zenghua Shao: 0000-0001-5000-3216
Nanxi Liu: 0000-0002-9524-1952
Haorong Lu: 0000-0003-0132-8166
Xiaoteng Fu: 0000-0001-7937-6980
Liping Wang: 0000-0001-7660-7288
Xin Liu: 0000-0003-3256-2940
Shanshan Liu: 0000-0002-5756-1728
Zongze Shao: 0000-0002-4784-090X

Abstract

Background

The scaly-foot snail (*Chrysomallon squamiferum*) is highly adapted to deep-sea hydrothermal vents and has drawn much interest since its discovery. However, the limited information on its genome has impeded further related research and understanding of its adaptation to deep-sea hydrothermal vents.

Findings

Here, we report the whole-genome sequencing and assembly of the scaly-foot snail and another snail (*Gigantopelta aegis*), which inhabits similar environments. Using Oxford Nanopore Technology, 10X Genomic, and Hi-C technologies, we obtained a chromosome-level genome of *C. squamiferum* with an N50 size of 20.71 Mb. By constructing a phylogenetic tree, we found that these two deep-sea snails are independent of other snails. Their divergence from each other occurred approximately 66.3 million years ago. Comparative genomic analysis showed that different snails have diverse genome sizes and repeat contents. Deep-sea snails have more DNA transposons and LTRs, but fewer LINEs, than other snails. Gene family analysis revealed that deep-sea snails experienced stronger

selective pressures than freshwater snails, and the nervous system, immune system, metabolism, DNA stability, antioxidation and biomineralization-related gene families were significantly expanded in scaly-foot snails. We also found 251 H-2 class II histocompatibility antigen, A-U alpha chain-like (*H2-Aal*) genes, which exist uniquely in the *Gigantopelta aegis* genome. This finding is important for investigating the evolution of major histocompatibility complex (MHC) genes.

Conclusion

Our study provides new insights into deep-sea snail genomes and valuable resources for further studies.

Keywords: Deep-sea snails; Genome assembly; Comparative genomics; Biomineralization;

Background

The discovery of deep-sea hydrothermal vents in the late 1970s expanded our knowledge of the extent of life on Earth [1]. Deep-sea macrobenthos, which are animals that inhabit deep-sea hydrothermal vents, face high hydrostatic pressure, variable temperatures and pH, and high levels of hydrogen sulphide, methane, and heavy metals [2]. To date, the literature contains a limited number of studies on the genetics of macrobenthos. A recent report on the genome of deep-sea hydrothermal vent/cold seep mussels (*Bathymodiolus platifrons*) showed that, while most of the genes present in a related shallow-water mussel (*Modiolus philippinarum*) have been retained, many gene families have expanded in the *B. platifrons* genome. These families include those that are associated with stabilising protein structures, removing toxic substances from cells, and the immune response to symbionts [3].

Gastropods represent the largest class of the phylum Mollusca, with different estimates of diversity varying from 80,000 to 150,000 species [4]. More than 218 gastropod (i.e. snail and slug) species have been described from chemosynthetic ecosystems (i.e. solely rely on endosymbiotic bacteria for sustenance), of which more than 138 are believed to be endemic to these ecosystems [5]. Gastropods are an important component of the fauna in hydrothermal vents in terms of abundance and biomass [6]. Due to the lack of samples and fossil evidence, studies on the evolution and adaptation of deep sea chemosynthetic gastropods are very limited. The scaly-foot snail: *Chrysomallon squamiferum* (*C. squamiferum*) is only found in hydrothermal vents at a depth of ~3,000-metres in the Indian Ocean. There are two types of varieties: black (due to greigite, which is an iron sulphide mineral that covers its exterior) scaly-foot individuals from the Kairei field on the central Indian ridge and Longqi field on the Southwest Indian ridge, and white scaly-foot individuals from the Solitaire field on the Central Indian Ridge [7] and Wocan field on the Carlsberg ridge of Northwest Indian ocean (this study). In particular, *C. squamiferum* has been included in the International Union for Conservation of Nature (IUCN) Red List of Endangered Species on July 18, 2019 [8]. Furthermore, the recently reported whole genome of the black scaly-foot snail was reported recently, which highlighted its evolved defence mechanisms of biomineralised armour [9]. *Gigantopelta* spp. is a major megafaunal gastropod genus found in some hydrothermal fields. The genus includes two species, *Gigantopelta chessoia* from East Scotia Ridge and *Gigantopelta aegis* from the

Southwest Indian Ridge [6]. Both *Chrysomallon* and *Gigantopelta* are members of the family Peltospiridae. They live in high-density aggregations and share several features, such as a large body size (up to > 45 mm, compared to typical sizes in other taxa of 10-15 mm, a 10-50 fold increase in body volume) and an enlarged oesophageal gland [10].

In this study, we sequenced and assembled genomes of the white scaly-foot snail *Chrysomallon squamiferum* (NCBI: txid216257; marinespecies.org:taxname:736932) (**Figure 1a**), which differ from the published genomes of the black varieties, from the Wocan field on the Carlsberg ridge of Northwest Indian ocean and *Gigantopelta aegis* (NCBI: txid1735272; marinespecies.org:taxname:853164) *(G. aegis*, **Figure 1a**) from the Southwest Indian Ridge. We gained insights into the evolution, gene family expansions, and adaptations of these extremophile gastropods.

Data Description

Genome assembly and annotation

The *C. squamiferum* genome was sequenced using a combination of sequencing libraries – 10X Genomics, Oxford Nanopore Technology (ONT), and Hi-C – to generate ~369.03 Gb of raw data (**Table S1**). Due to the limited sample material, *G. aegis* was sequenced from whole genome shotgun libraries (with 350 bp to 10 kb inserts on the BGISEQ-500, RRID:SCR_017979) to generate 910.08 Gb of raw data (**Table S2**). The genome of *C. squamiferum* was assembled with long ONT reads by using Canu v1.7 (Canu, RRID:SCR 015880) [11] and WTDBG (WTDBG, RRID:SCR_017225) [12]. After polishing the genome with 10X Genomics sequencing data, a 454.58 Mb assembly (a little smaller than the estimated genome size: 495 Mb, **Figure S1**) with 6,449 contigs and an N50 of 541.32 kb was generated (**Table S3**). Next, Hi-C data were used to anchor the assembly, yielding a 16-chromosome assembly (**Figure 1b**). This effort increased the N50 size to ~20.71 Mb (**Table 1**). The 16 chromosomes cover ~80% of the whole genome, and the average length, maximal length, and minimal length of the 16 chromosomes were 22.67, 46.78, and 10.64 Mb, respectively, (**Table S4**). A Benchmarking Universal Single-Copy Orthologs (BUSCO, RRID:SCR_015008) completeness score of 94.8% for this genome suggested that it was of good quality (**Table S5**). An approximately 1.29 Gb (a little smaller than the estimated genome size:1.50 Gb, **Figure S1**) genome assembly of *G. aegis* with a scaffold

N50 of 120.96 kb (**Table S6**) and a BUSCO completeness score of 92.4% (**Table S7**) was obtained using Platanus (Platanus, RRID:SCR_015531) [13]. After masking repeat elements, we employed homologous and *de novo* prediction methods to construct gene models for the two genomes, obtaining 28,781 *C. squamiferum* genes and 25,601 *G. aegis* genes (**Tables S8** and **S9**). The gene sets were functionally annotated using KEGG (KEGG, RRID:SCR_012773), Swiss-Prot (UniProt, RRID:SCR_002380), InterPro (InterPro, RRID:SCR_006695), and TrEMBL (TrEMBL, RRID:SCR_002380) (**Tables S10** and **S11**).

Genome sizes and repeat contents.

The genome assembly sizes of *C. squamiferum* (~455.36 Mb) and *G. aegis* (~1.29 Gb) differed from those of freshwater snails (~916 Mb (*Biomphalaria glabrata*) [14] and ~440 Mb (*Pomacea canaliculate*) [15]), which suggests that there is significant genome size diversity within snails (**Figure 1c**). In the absence of ploidy effects [16, 17], differences in genome size often stem from the accumulation of various repetitive elements. A comparison of the repeat elements (**Figure 1c** and **Table S12**) supported this trend. The genomes of *C. squamiferum* and *P. canaliculate* (smaller genome sizes) contained fewer repeats than *B. glabrata* and *G. aegis*, whereas *G. aegis* had more repeats than *B. glabrata* (**Figure 1d**). This finding suggests that snail genome sizes correlate with repeat content. Despite the similar genome sizes of *C. squamiferum* and *P. canaliculata*, their genome landscapes were distinct. For example, ~10.17% of the *C. squamiferum* genome consisted of tandem repeats compared to ~2.89% in *P. canaliculata* (**Table S12**). DNA transposons and LTRs comprise ~17.73% and ~5.99% of the *C. squamiferum* genome, respectively, but only ~6.84% and ~3.53% in *P. canaliculata*. LINEs made up ~8.63% of the *P. canaliculata* genome compared to ~5.65% in *C. squamiferum*. Similarly, although the larger *G. aegis* and *B. glabrata* genomes have similar proportions of tandem repeats, *G. aegis* had a higher percentage of DNA transposons (~32.15% versus ~20.20%) and LTRs (~13.32% versus ~3.75%). LINEs made up ~23.93% of the *B. glabrata* genome compared to ~11.51% in *G. aegis*. Taken together, these data suggest that deep-sea hydrothermal vent snail genomes have more DNA transposons and LTRs and fewer LINEs than their freshwater counterparts. In particular, DNA/CMC-EnSpm, DNA/TcMar–Tc1, and

DNA/DNA were the main factors that caused the differences in DNA transposon content in the four snail genomes (**Figure 1d**). We found that LINE/L2, LINE/RTE-BovB, LINE/LINE, and LINE/CR1 were much higher in fresh-water snail genomes than in deep-sea snails. Although most of the precise functions of these repeats have not been studied in depth, repeats have been thought to have a regulatory function in related genes that play an important role in the life cycle and can introduce great genome flexibility [18]. Also, in the mammalian genome, transposons have been described as redundant enhancers that regulate their target genes, which are higher or specific tissue expressed, indicating the importance of transposons[19]. Thus, we might infer that the expansion of DNA transposons and LTRs, as well as the absence of some LINEs, may be closely associated with important genes that help these deep-sea snails adapt to extreme environments.

Construction of phylogenetic relationships for deep-sea snails

To determine the phylogenetic relationships between deep-sea snails and other molluscs, we compared their genomes with those from two shallow-water bivalves (*P.fucata* and *C.gigas*) and four shallow-water gastropods, including two fresh-water snails (*B. glabrata* and *P. canaliculate*), one limpet (*B. glabrata*) and one sea slug (*A. californica*). The genomes of the California two-spot octopus (*O. bimaculoides*) and the freshwater leech (*H.robusta*) were used as the outgroup (**Figure 2a**). We identified 26,668 gene families in the ten species examined (**Table S13**). Phylogenetic trees were constructed from 406 shared single-copy orthologs. Both maximum likelihood (ML) and Bayesian methods revealed the same topology (**Figure 2a** and **Figure S2**), which is consistent with a recent study [15]. In the tree, bivalves and gastropods are clearly separated and the two deep-sea snails are located on the same branch and are independent of other snails (although their genome sizes are quite different). We estimated that *C. squamiferum* and *G. aegis* diverged from a common ancestor approximately 66.3 million years ago (MYA).

Demographic histories of the deep-sea snails

Based on these two assembled genomes, we estimated their historical effective population size (N_e) using whole-genome genetic variation. We identified ~3.51 and ~3.19 million heterozygous SNPs with nucleotide diversities of 0.0077 and 0.0025 for *C. squamiferum* and *G. aegis*, respectively. We estimated changes in N_e using the pairwise

sequential Markovian coalescent (PSMC, RRID:SCR_017229) method, which can infer demography from approximately 20,000 to 1 MYA [20]. The effective population sizes of *C. squamiferum* and *G. aegis* – species derived from different geographical locations in the Indian Ocean – are distinct (**Figure 2b**). In the demographic history of *G. aegis* decreased until ~250 thousand years ago, followed by an N_e increase, from ~50,000 to 450,000 individuals, 20,000 years ago. Several cycles of increasing and decreasing N_e have been observed for *C. squamiferum*, with the effective population size recovering and stabilising at 35,000 individuals approximately 70 thousand years ago. Thus, although deep-sea habitats are inhabited, deep-sea snail populations are sensitive to habitat disturbances. It was reported that vent organisms are exquisitely sensitive to nuances in fluid flux, such as chemical compositions, temperature, geological setting and biological interactions[21].Our results revealed that the demographic histories of these two snails differd because their habitat conditions are markedly different.

Evolution of single-copy orthologous genes

To explore the evolutionary rate of single-copy orthologous genes, we calculated the synonymous substitution rate (*Ka*) and nonsynonymous substitution rate (*Ks*) values of 1,324 single-copy orthologous genes shared by the two deep-sea snails, one shallow-water limpet (*L. gigantea*), and two freshwater snails (*B. glabrata* and *P. canaliculate*) using Codeml in the PAML package (PAML, RRID:SCR_014932) [22] (**Figure 2c**, **Figure S3,** and **Table S15**). We found that the *Ka* values of the two deep-sea snails (average: 0.37 and 0.41) were higher (Mann-Whitney U test, p-value<0.001) than that of the shallow-water limpet (0.35) but similar to those of two freshwater snails (0.39 and 0.41), which suggests that the genes of deep-sea and freshwater snails both evolved faster after their divergence from the shallow-water limpet. The *Ks* values of the deep-sea (3.34 and 3.09) and fresh-water (3.19 and 3.24) snails were also similar to and lower (Mann-Whitney U test, p-value<0.001) than those of the shallow-water limpet (3.72). Additionally, the *Ka*/*Ks* values of the deep-sea snails (average: 0.13 and 0.15) were approximately ~20% and ~40% higher (Mann-Whitney U test, p-value<0.001) than those of the shallow-water limpet (0.11). From these findings, we could infer that deep-sea snails have experienced stronger selective

pressures than the shallow and fresh water species discussed here, possibly to allow adaptation to life in hydrothermal vents.

Expanded gene families in deep-sea snail genomes

Nervous system

Using CAFE (CAFE, RRID:SCR_005983) [23] (*see details in Methods*), we identified two significantly (*p*-value < 0.01) expanded gene families in the two deep-sea snail genomes compared to the freshwater snails and shallow-water limpet. The BTB/POZ domain-containing protein 6 (*BTBD6*) had 56 copies in *C. squamiferum* and 35 copies in *G. aegis*, while fewer than 5 copies were found in the four other snail species examined (**Figure 3a**). We found 17 *BTBD6* genes on chromosome 16 of *C. squamiferum*, and these genes showed traces of tandem duplications (**Figure 3b**). In *G. aegis*, we also found several tandem gene clusters (**Figure 3b**). *HTR4* (5-hydroxytryptamine receptor 4) had 12 copies in *C. squamiferum* and 18 copies in *G. aegis*, while only one copy was found in the other snail species (**Figure 3c**). The expansions of these gene families also displayed tandem duplications (**Figure S4**). Both of these genes have roles in neuroregulation; *BTBD6*, which is an adaptor of the Cul3 ubiquitin ligase complex and is essential for neural differentiation [24], while *HTR4* modulates the release of various neurotransmitters [25]. A previous study revealed that a large unganglionated nervous system exists in *C. squamiferum* [7]. We speculate that the expansions of *BTBD6* and *HTR4* contribute to this system by sustaining life in a deep-sea environment.

Metabolism related genes

C. squamiferum houses abundant endosymbionts in its greatly enlarged oesophageal gland and these endosymbionts supply nutrition for its host. KEGG enrichment analysis on the 183 expanded gene families of *C. squamiferum* revealed significant enrichment for metabolic pathways (*q*-value < 0.0001, **Table S16**). Among these genes, nine gene families encoded enzymes in the glycolysis pathway and citrate cycle (TCA cycle). For example, the genes for isocitrate dehydrogenase (IDH), which catalyses the oxidative decarboxylation of isocitrate to produce α-ketoglutarate and CO2, expanded significantly (*p* < 0.01). The α-ketoglutarate dehydrogenase complex (OGDC) consists of three

components: oxoglutarate dehydrogenase (OGDH), dihydrolipoyl succinyltransferase (DLST), and dihydrolipoyl dehydrogenase (DLD), among which the genes for OGDH were expanded ($p < 0.01$, **Figure 4a**). IDH and OGDC are two rate-limiting enzymes in the TCA cycle, and the related biochemical reactions are irreversible (**Figure 4b**).

Defence mechanisms

Endosymbiotic bacteria are critical for snail life in deep-sea hydrothermal vent ecosystems [26]. These bacterial taxa are largely restricted to chemosynthetic environments, with some being exclusive to vents [27]. The divergent evolution of the *C. squamiferum* and *G. aegis* genomes may have generated diverse defence mechanisms.

A total of 183 expanded gene families were identified in the *C. squamiferum* genome. As expected, many of these families have roles in the immune system. However, unlike the freshwater snail *B. glabrata* [14] and deep-sea mussels [3], we did not detect an expansion of the Toll-like receptor 13 (*TLR13*) gene family, but identified other expanded gene families (**Figure 4a**). For example, increased expression of thioredoxin 1 (*Txn1*; 22 copies in *C. squamiferum*) was identified. Thioredoxin 1 (*Txn1*), a redox protein, is important for regulating of cellular redox homeostasis and anti-apoptotic functions. Txn1 stimulates cell proliferation and cell cycle progression, induces hypoxia-inducible factor-1α (HIF-1α) and angiogenesis, and alters the balance between the matrix metalloproteinases and their tissue inhibitors [28, 29]. Txn1 also plays a pivotal role in T cell activation in mice [30]. Although T-cell related adaptive immunity only appears in vertebrates, the existence and expansion of this gene may assist the innate immune system of *C. squamiferum*. Glutamine-fructose-6-phosphate transaminase (*GAFT*; 21 copies in *C. squamiferum*) promotes the biosynthesis of chitin [31, 32], which is one of the stable components of the crustacean shell and provides protection against predation and infection.

We identified expanded gene families that maintain the stability of nucleic acids and proteins, such as heat shock protein 90 (Hsp90; 13 copies in *C. squamiferum*, **Figure 4a**), which protects proteins against heat stress [33]; the single-stranded DNA-binding proteins, encoded by SSB genes (19 copies in *C. squamiferum*, and 1 copy in other species, **Figure 4a)**, which are required for DNA replication, recombination, and repair processes [34]; and catalase (*CAT*, 6 copies *C. squamiferum*; **Figure 4c**), which is critical in the response

against oxidative stress [35]. The elevated levels of heavy metals and sulphide and high temperatures in hydrothermal vents are likely to greatly increase the risk of DNA damage and misfolded proteins. Thus, these expanded gene families may help these snails resist environmental stress.

We also found a special gene family, deleted in malignant brain tumours 1 (*DMBT1*), expanded (70 copies, **Figure 4a**) in the *C. squamiferum* genome. *DMBT1* can encode three glycoproteins (DMBT1 (deleted in malignant brain tumours 1 protein), SAG (salivary agglutinin), and GP340 (lung glycoprotein-340)) and belongs to the scavenger receptor cysteine-rich (SRCR) protein superfamily of the immune system [36]. This gene consists of the SRCR, CUB, and zona pellucida domains, and all 70 copies of this gene in *C. squamiferum* contain the SRCR domain, which can bind a broad range of pathogens, including cariogenic *streptococci*, *Helicobacter pylori*, and HIV [37]. However, previous studies have shown that SRCR domains that contain proteins are commonly expressed in the shell martrix [38] and have been proven to be potentially linked to biomineralization [39], which would be associated with the foot scales of *C. squamiferum*. Nonetheless, the expansion of this gene family will either strengthen the immune ability or help construct the scale armour of these snails.

Correspondingly, we identified the expansion of 198 gene families (containing 4,515 genes) in the *G. aegis* genome. These families were enriched in 58 KEGG pathways (*q*value < 0.05) (**Table S17**). The majority of these pathways were associated with the immune and disease response, and included terms such as ‘infection’, ‘NOD-like receptor signalling’, ‘Tumour necrosis factor (TNF) signalling pathway’, and ‘Antigen processing and presentation’ (**Figure S5**). Surprisingly, we found 251 copies of the H-2 class II histocompatibility antigen, A-U alpha chain-like (H2-Aal) genes, which is one of the major histocompatibility complex (MHC) genes in vertebrates [40]. The existence and super expansion of this gene family in the invertebrate positions in *G. aegis* is useful for the study of immune system evolution.

Discussion

Molluscs are a highly diverse group, and their high biodiversity makes them an excellent model to address topics such as biogeography, adaptability, and evolutionary processes

[41]. Members of the family Peltospiridae in the gastropod clade Neomphalina are restricted to chemosynthetic ecosystems and, so far, are only known from hot vents [6]. Based on the chromosome-scale genome assembly analyses of the scaly-foot snail (*C. squamiferum*) and deep-sea snail (*G. aegis*), which both belong to the Peltospiridae family from chemosynthetic ecosystems, our results provide insight into the possible evolution and adaptation mechanisms of hydrothermal vent animals.

By constructing a phylogenetic tree, we found that snails diverged from other molluscs approximately 555.2 MYA (**Figure 2a**). These two deep-sea snails were found to be independent of other shallow-water gastropods around 536.6 MYA. At the end of the Cretaceous geological period, approximately 66.3 MYA, *C. squamiferum* and *G. aegis* diverged from each other and later had different *Ne* (**Figure 2b**). This finding indicated that they faced different environmental factors and selected pressures. This evolutionary time frame implies that the last common ancestor of all molluscs (LCAM) already lived before the Cambrian Explosion (530-540 MYA), which was also speculated by the palaeobiological hypothesis [42]. It also elucidated that deep-sea gastropod lineages originated at least around 540 MYA and diverged from other gastropods in the same age of the oldest molluscs taxons, Aculifera and Conchifera [43, 44]. The deep-sea gastropod lineages were also confirmed by the phylogenetic analysis of mitogenomes [45]. Further conceived by the evolutionary rate of single-copy orthologous genes, deep-sea gastropod lineages have experienced stronger selective pressures than shallow-water gastropods (**Figure 2c**).

Transposable elements (TEs) play multiple roles in driving genome evolution in eukaryotes[46]. The genome sizes of four representative snails were quite divergent (440 Mb-1.29 Gb). The deep-sea snail *G. aegis* had the largest genome (1.29 Gb), with the highest percentage of DNA transposons (32.15%). Deep-sea snails (*C. squamiferum* and *G. aegis*) had more DNA transposons and LTRs than other snails, but fewer LINEs. LTR class has been identified as the main contributor to open chromatin regions and transcription factor binding sites [47, 48]. LINEs may be associated with the duplicability of genomic regions, which are always shared between related lineages[49]. Thus, the higher portions of DNA transposons and LTRs may be the results of genome evolution due

to environmental changes and associated with the ability of deep-sea snails to adapt to extreme environments.

Specifically, we analysed expanded gene families in deep-sea snail genomes (**Figure 4a**). They both significantly expanded the nervous system, especially *BTBD6* and *HTR4*, which are involved in the neuroregulation of activities, such as movement, predation, and resistance to environmental change. As for the chemosynthetic snails, they both had expanded immune system-related gene families. In the *C. squamiferum* genome, the expansions of *Txn1* and *GAFT* were found. In the *G. aegis* genome, different immune and disease response genes families were expanded, such as *H2-Aal* genes. These expanded gene families were different from those found in fresh water snails and deep-sea mussels.

Interestingly, in the scaly-foot snail (*Chrysomallon squamiferum*) genome, genes involved in the main metabolic pathways were significantly enriched, including the glycolysis pathway and the citrate cycle (TCA cycle) . Other enriched gene families included the single-stranded DNA-binding protein (*SSB)* family, which stabilise ssDNA; heat shock protein 90 (*Hsp90*) family, which keep proteins folded properly; and the catalase (*CAT*) family, which prevents the generation of free radicals due to exposure to peroxides. The expansions of these gene families may have provided deep-sea snails with better immune reactions with symbionts, rapid nerve signal conduction, stronger metabolism, and effective resistance while adapting to their hydrothermal vent habitat.

In particular, we found that *DMBT1* gene families that encode multiple SRCR domains were expanded significantly in *C. squamiferum*. These genes play important roles in immune response and biomineralisation, both of which are vital for deep-sea chemosynthetic snail.

In conclusion, the genome analysis of deep-sea snails (*C. squamiferum* and *G. aegis*) from hydrothermal vents revealed mechanisms of their evolution and molecular adaptations to extreme environments, and will be a valuable resource for studying the evolution of invertebrates.

Materials and Methods

Sample collection and DNA isolation

C. squamiferum samples were obtained from the Wocan vent field (60.5°E 6.4°N, 2919m depth) on the Carlsberg Ridge, northwest Indian Ocean, in March 2017 during the Chinese DY38th cruise. *G. aegis* samples were obtained from the Longqi vent field (37.5°S, 49.4°E, 2,780 m) on the southwest Indian ridge in March 2015 during the Chinese DY35th cruise. DNA was extracted from the muscle sample of one individual using the cetyl trimethylammonium bromide (CTAB) method and a DNeasy blood & tissue kit (QIAGEN). DNA quality and quantity were checked using pulsed field gel electrophoresis and a Qubit Fluorometer (Thermo Scientific).

Libraries preparation and sequencing

Whole Genome Shotgun Sequencing

Four WGS libraries were prepared for sequencing: one short insert size library (350 bp) and three mate-pair large insert size libraries (2 kb, 5 kb, and 10 kb). Libraries were constructed using an MGI Easy FS DNA Library Prep Set kit (MGI, China). Paired-end reads (100 bp) and mate-pair reads (50 bp) were obtained from the BGISEQ-500 platform.

10X Genomics sequencing

To prepare the Chromium library, 1 ng of high quality DNA was denatured, spiked into the reaction mix, and mixed with gel beads and emulsification oil to generate droplets within a Chromium Genome chip. Then, the rest of the steps were completed following the standard protocols for performing PCR. After PCR, the standard circularisation step for BGISEQ-500 was carried out, and DNA nanoballs (DNBs) were prepared [50]. Paired-end reads with a length of 150 bp were generated on the BGISEQ-500 platform [51].

Oxford Nanopore Technologies

DNA for long-read sequencing was isolated from the muscle tissues of our samples. Using 5 flow cells and the ONT chemistry for the GridION X5 sequencer (GridION, RRID:SCR_017986).

following manufacturer's protocols, we generated 39.61 Gbp of raw genome sequencing data.

Hi-C **library and sequencing**

The Hi-C library was prepared following the standard *in situ* Hi-C [52] protocol for muscle samples, using *DpnII* (NEB, Ipswich, America) as the restriction enzyme. After that, a standard circularization step was carried out, followed by DNA nanoballs (DNB) preparation following the standard protocols of the BGISEQ-500 sequencing platform as previously described [50]. Paired-end reads with a length of 100 bp were generated on the BGISEQ-500 platform[51].

Genome assembly

For the genome assembly of *Chrysomallon squamiferum*, Canu v1.7 was first used to perform corrections of ONT reads with the parameters "correctedErrorRate=0.105 corMinCoverage=0 minReadLength=1000 minOverlapLength=800". Then, wtdbg (v1.2.8) was used to assemble the genome with the parameters "--tidy-reads 3000 -k 0 -p 21 -S 4 --rescue-low-cov-edges" using corrected reads generated by Canu. Next, we made use of the sequencing reads from the 10X Genomic library to carry out genome polishing using Pilon v1.22 (Pilon, RRID:SCR_014731) with its default parameters. Quality control of Hi-C sequencing reads was first performed using the HiC-Pro pipeline (HiC-Pro, RRID:SCR_017643) [53] with the parameters "[BOWTIE2_GLOBAL_OPTIONS = --very-sensitive -L 30 --score-min L,-0.6,-0.2 --end-to-end –reorder; BOWTIE2_LOCAL_OPTIONS = --very-sensitive -L 20 --score-min L,-0.6,-0.2 --end-to-end –reorder; IGATION_SITE = GATC; MIN_FRAG_SIZE = 100; MAX_FRAG_SIZE = 100000; MIN_INSERT_SIZE = 50; MAX_INSERT_SIZE = 1500]". In total, 23,646,810 pairs of valid reads were obtained. Next, the valid Hi-C data was used to anchor the nanopore contigs onto chromosomes separately by applying the 3D-DNA pipeline [54]. The contact maps were then generated by the Juicer pipeline [55], and the boundaries for each chromosome were manually rectified by visualising the inter.hic file in Juicebox [56]. 16 chromosomes were identified by combining the linkage information from the agp file.

For the genome assembly of *G. aegis*, we obtained only WGS sequencing reads because of limited DNA and tissue samples. Platanus v1.2.4 [13] was used to assemble the genome with WGS clean data with the parameters "assemble –k 29 –u 0.2, scaffold -l 3 -u 0.2 -v 32 -s 32 and gap_close –s 34 –k 32 –d 5000". BUSCO v2 were used to evaluate genome assemblies with the metazoan_odb9 database.

Genome annotation

Repeat annotation

Homolog-based and *de novo* prediction methods were used to detect repeat contents. In particular, RepeatMasker v4.0.5 (RepeatMasker, RRID:SCR_012954) [57] and RepeatProteinMask v4.0.5 (RepeatProteinMask, RRID:SCR_012954) were used to detect transposable elements against the Repbase database[58] at the nuclear and protein levels, respectively. RepeatMasker was used again to detect species-specific transposable elements against databases generated by RepeatModeler v1.0.8 (RepeatModeler, RRID:SCR_015027) and LTR-FINDER v1.0.6 (LTR-FINDER, RRID:SCR 015247) [59]. Moreover, Tandem Repeat Finder v4.0.7 [60] was utilised to predict tandem repeats.

Gene annotation

We combined homology-based and *de novo* evidence to predict protein-coding genes in two genomes. For the homology-based method, we used six relative gene sets of *Aplysia californica*, *Bathymodiolus platifrons*, *Biomphalaria glabrata*, *Lottia gigantea*, *Modiolus philippinarum*, and *Pomacea canaliculata*. First, these homologous protein sequences were aligned onto each assembled genome using TBLASTN (TBLASTN, RRID_SCR 011822), with an *E*-value cut-off of 1×10^{-5}, and the alignment hits were linked to candidate gene loci by GenBlastA [61]. Second, we extracted genomic sequences of candidate gene regions, including 2 kb flanking sequences, and then used GeneWise v2.2.0 (GeneWise, RRID:SCR_015054) [62] to determine gene models.

In the *de novo* method, we used Augustus (Augustus, RRID:SCR_008417)[63] to predict the gene models on repeat-masked genome sequences. We selected high-quality genes with intact open reading frames (ORFs) and the highest GeneWise [62] score from a homology-

based gene set to train Augustus with default parameters before prediction. Gene models with incomplete ORFs and small genes with protein-coding lengths less than 150 bp were filtered out. Finally, a BLASTP (BLASTP, RRID:SCR_001010) search of predicted genes was performed against the Swiss-Prot database [64]. Genes with matches to Swiss-Prot proteins containing any one of the following keywords were filtered: transpose, transposon, retrotransposon, retrovirus, retrotransposon, reverse transcriptase, transposase, and retroviral. Finally, the results of the homology- and *de novo*-based gene sets were merged using GLEAN (GLEAN, RRID:SCR_002890)[65] to yield a nonredundant reference gene set.

Gene function annotation

We annotated the protein-coding genes by searching against the following public databases: Swiss-Prot [66], the Kyoto Encyclopedia of Genes and Genomes [67], InterPro [68], and TrEMBL [66].

Phylogenetic tree reconstruction and divergence time estimation

The TreeFam tool (Tree families database, RRID:SCR 013401) [69] was used to identify gene families as follows: first, all the protein sequences from a selection of 10 representative species (8 species including *Aplysia californica* (GCF_000002075.1), *Octopus bimaculoides* (GCF_001194135.1), *Biomphalaria glabrata* (GCF_000457365.1), *Crassostrea gigas* (GCF_000297895.1), *Lottia gigantea* (GCF_000327385.1), *Pomacea canaliculate* (GCF_003073045.1), *Pinctada fucata* (GCA_002216045.1), *Helobdella robusta* (GCF_000326865.1) from the NCBI database, *C. squamiferum* and *G. aegis* from this research) were compared using blastp with the *E*-value threshold set as 1e-7. Then, alignment segments of each protein pair were concatenated using the in-house software Solar v0.9.6 [69]. H-scores were computed based on Bit-scores and were used to evaluate the similarity among proteins. Finally, gene families were obtained by clustering homologous gene sequences using Hcluster_sg v0.5.0 [69].

We obtained 406 one-to-one single-copy orthology gene families based on gene family classification. Then, these gene families were extracted and aligned using guidance from

amino-acid alignments created using the default parameters of the MUSCLE (MUSCLE, RRID:SCR_011812) [70] programme. All sequence alignments were then concatenated to construct 1 super-matrix and then a phylogenetic tree was constructed under a GTR+gamma model for nucleotide sequences using ML and Bayesian methods. The same set of codon sequences were used for phylogenetic tree construction and estimation of divergence time. The PAML mcmctree programme [71, 72] was used to determine divergence times with the approximate likelihood calculation method, and the correlated molecular clock and REV substitution model. The concatenated coding sequences of one-to-one orthologous genes and the phylogenomics topology were used as inputs. We used five calibration time points based on fossil records: *A. californica* - *C. gigas* (~516.3 - 558.3 MYA), *A. californica* - *P. canaliculata* (~310 – 496 MYA), *A. californica* - *Octopus bimaculoides* (~551 – 628 MYA), *C. gigas* - *H. robusta* (~585 – 790 MYA), and *C. gigas*–*P. fucata* (394 MYA) (http://www.timetree.org), were used as constraints in the MCMCTree estimation.

SNP calling and estimation of history population sizes

About 50X clean WGS reads were mapped to genomes of *C. squamiferum* and *G. aegis* using BWA-MEM (v0.7.12-r1039) (BWA, RRID:SCR_010910) [73] with default parameters respecitvely. Then, SAMtools (v0.1.19-44428cd) (Samtools, RRID:SCR_002105) [74] and "SortSam.jar" in the Picard package (v1.54) was used to converte and sort BAM files. Local realignment was again carried out using RealignerTargetCreator and IndelRealigner in GATK v3.6 (GATK, RRID:SCR_001876) [75] with default parameters. SNPs were identified using HaplotypeCaller and filtered using VariantFiltration with parameter "-filter-expression "QD < 2.0 || MQ < 40.0 || ReadPosRankSum < -8.0 || FS > 60.0" --filter-name LowQualFilter --genotype-filter-expression "DP < 5.0" --genotype-filter-name lt_5". Estimatation of the historical effective population sizes were carried out using PSMC v0.6.5-r67 [76]. Firstly, diploid genome references were constructed using SAMtools and BCFtools call with "samtools mpileup -C50" and "vcfutils.pl vcf2fq -d 20 -D 100". Secondly, the demographic history was inferred using PSMC with parameters '-N25 -t15 -r5 -p 4+25*2+4+6' [77].

Expansion and contraction of gene families

We used CAFE (Computational Analysis of gene Family Evolution) v2.1 [23] to analyse gene family expansion and contraction under the maximum likelihood framework. The gene family results from the TreeFam pipeline and the estimated divergence time between species were used as inputs. We used the parameters “-p 0.01, -r 10000, -s” to search for the birth and death parameter (λ) of gene families, calculated the probability of each gene family with observed sizes using 10,000 Monte Carlo random samplings, and reported birth and death parameters in gene families with probabilities less than 0.01.

Figure legends

Figure 1. Genome characteristics of *C. squamiferum* and *G. aegis*. a) Photos of two species. Left: *C. squamiferum*; right: *G. aegis.* Scale bar = 1cm. **b**) Heat map of chromatin interaction relationships at a 125 kb resolution of 16 chromosomes. **c**) Genome sizes and transposable elements in *C. squamiferum, G. aegis,* and two representative freshwater snail genomes. **d**) Distribution of repeat sub-types of four species.

Figure 2. Phylogenetic tree, estimated N_e, and evolution of single copy orthologous genes of deep-sea snails. **a)** Phylogenetic tree of ten representative molluscs. Expanded and contracted gene families were identified using CAFE. Divergence time was estimated using MCMCtree. Species names in red represent two deep-sea snails. Red dots represent calibration time from TimeTree database. The timescale also refers to the TimeTree database. **b)** Estimated demographic histories of two deep-sea snails. The generation time set to "3" refers to the land snail [78]. The μ values are calculated in **Table S15**. **c)**. Box plot of *K*a/*K*s values for five species.

Figure 3. Expansion of nervous system-related genes a) Phylogentic tree of *BTBD6* genes in the examined species. The grey ellipses mark different clusters of genes. **b**) Expansion pattern of *BTBD6* genes in two deep-sea snails. Grey lines represent scaffold sequences. Coloured rectangles represent *BTBD6* genes. Symbols "//" represent other genes along the scaffolds. The blue numbers: "1" represent only one gene between the tandem duplicated genes. **c**) Expansion of *HTR4* genes. The species legend in the middle was used for **a** and **c**. Gene trees of **a** and **c** were constructed using MUSCLE (v3.8.31)[70] and FastTree (v2.1.10)[79].

Figure 4. **Expansion of immune, metabolism, DNA stability, and antioxidation genes**. **a**) Gene numbers of four defence-related genes (*DMBT1*, *GAFT*, *Hsp90,* and *Txn1*), three metabolism-related genes (*OGDHE1*, *OGDHE2,* and *IDH*), and the *SSB* gene. **b**) TCA cycle signal pathway. The brown ellipses represent important enzymes and the expansion of these genes (*OGDHE1*, *OGDHE2,* and *IDH*). **c**) Expansion of the catalase (*CAT*) gene family in selected species.

Table 1. Genome assembly and annotation of *Chrysomallon squamiferum* and *Gigantopelta aegis*.

Species	*Chrysomallon squamiferum*	*Gigantopelta aegis*
Genome size	455.36 Mb	1.29 GB
Scaffold N50	20.7 Mb	120.96 kb
Contig N50	541.32 kb	6.96 kb
Number of genes	28,781	25,601
Repeat content	30.56%	64.17%
GC content	34.48%	37.45%
Complete BUSCO	94.80%	92.40%

Data and code availability

The genome assemblies of these two genomes have been deposited in GenBank under the accession number CNP0000854. The raw sequencing reads were also uploaded to the SRA database under accession number CNP0000854. All supporting data are available in the *GigaScience* GigaDB database [80].

Additional Files

Additional File 1: Supplementary Figures and Tables.docx

Abbreviations

ONT: Oxford Nanopore Technology; Hi-C: chromosome conformation capture; DNA: Deoxyribonucleic acid; LTR: long terminal repeat; LINEs: Long interspersed nuclear elements; MHC: major histocompatibility complex; IUCN: International Union for Conservation of Nature; Gb: gigabase pairs; Mb: megabase pairs; kb: kilobase pairs; bp: base pairs; BUSCO: Benchmarking Universal Single-Copy Orthologs; KEGG: Kyoto Encyclopedia of Genes and Genomes; CR1: chicken repeat 1; ML: Maximum Likelihood; MYA: million years ago; PSMC: pairwise sequential Markovian coalescent;

SNP: Single Nucleotide Polymorphism; K/Pg: Cretaceous/Paleogene; PAML: Phylogenetic Analysis by Maximum Likelihood; CAFE: Computational Analysis of gene Family Evolution; BTBD6: BTB/POZ domain-containing protein 6; HTR4: 5-hydroxytryptamine receptor 4; IDH: isocitrate dehydrogenase; TCA: tricarboxylic acid; OGDC: α-ketoglutarate dehydrogenase complex; DLD: dihydrolipoyl dehydrogenase; OGDH: oxoglutarate dehydrogenase; DLST: dihydrolipoyl succinyltransferase; CO2: carbon dioxide; TLR13: Toll-like receptor 13; Txn1: Thioredoxin 1; HIF-1α: hypoxia-inducible factor-1α; GAFT: glutamine-fructose-6-phosphate transaminase; CAT: catalase; DMBT1: deleted in malignant brain tumours 1; GP340; glycoprotein-340; SAG: salivary agglutinin; SRCR: scavenger receptor cysteine-rich; HIV: human immunodeficiency virus; TNF: Tumour necrosis factor; LCAM: last common ancestor of all molluscs; TEs: The transposable elements; Hsp90: heat shock protein 90; PCR: Polymerase chain reaction; DNBs: DNA nanoballs; WGS: whole genome sequence; BWA: Burrows-Wheeler-Alignment; GATK: Genome Analysis Toolkit; NCBI: National Center for Biotechnology Information; SRA: Sequence Read Archive.

Author contributions

Z.S., S.L., G.F., and X.L. conceived and managed this project and amended the manuscript. X.Z., Y.Z., L.M., and I.S. performed the evolutionary analysis and wrote the manuscript. L.M., J.C., and Y.S. performed genome assembly and annotation. J.B., S.L., X.F., C.W., Z.S., H.L., N.L., and L.W. were responsible for sample collection, DNA extraction, and library construction.

Acknowledgments

We thank Dr. Yadong Zhou for providing the animal photographs. We are very grateful to the RV "Xiangyanghong 9" crew and HOV "Jiaolong" operation teams for helping us

collect samples from the deep-sea hydrothermal vent field. This work was supported by the National Key R&D Programme of China (No. 2018YFC0310702).

Supplemental information

Supplemental Information can be found online.

Declaration of interests

The authors declare that they have no competing interests.

References

1. Corliss JB, Dymond J, Gordon LI, Edmond JM, Von Herzen RP, Ballard RD, et al. Submarine Thermal Springs on the Galápagos Rift. Science. 1979;203 4385:1073-83.
2. VAN DOVER CL. The ecology of deep-sea hydrothermal vents. Princeton: Princeton University Press; 2000.
3. Sun J, Zhang Y, Xu T, Zhang Y, Mu H, Zhang Y, et al. Adaptation to deep-sea chemosynthetic environments as revealed by mussel genomes. Nature ecology & evolution. 2017;1 5:0121.
4. Parkhaev PY. The Cambrian 'basement'of gastropod evolution. Geological Society, London, Special Publications. 2007;286 1:415-21.
5. Sasaki T, Warén A, Kano Y, Okutani T and Fujikura K. Gastropods from Recent Hot Vents and Cold Seeps: Systematics, Diversity and Life Strategies. 2010.
6. Chong C, Linse K, Copley TJ and Rogers DA. The 'scaly-foot gastropod': a new genus and species of hydrothermal vent-endemic gastropod (Neomphalina: Peltospiridae) from the Indian Ocean. 2015;81 3:322-34.
7. Chen C, Copley JT, Linse K and Rogers AD. Low connectivity between 'scaly-foot gastropod' (Mollusca: Peltospiridae) populations at hydrothermal vents on the Southwest Indian Ridge and the Central Indian Ridge. Organisms Diversity & Evolution. 2015;15 4:663-70.
8. Sigwart JD, Chen C, Thomas EA, Allcock AL, Bohm M and Seddon M. Red Listing can protect deep-sea biodiversity. Nature Ecology and Evolution. 2019;3 8:1134-.
9. Sun J, Chen C, Miyamoto N, Li R, Sigwart JD, Xu T, et al. The Scaly-foot Snail genome and implications for the origins of biomineralised armour. Nature communications. 2020;11 1:1-12.
10. Chen C, Uematsu K, Linse K and Sigwart JD. By more ways than one: Rapid convergence at hydrothermal vents shown by 3D anatomical reconstruction of Gigantopelta (Mollusca: Neomphalina). BMC Evolutionary Biology. 2017;17 1:62.
11. Koren S, Walenz BP, Berlin K, Miller JR, Bergman NH and Phillippy AM. Canu: scalable and accurate long-read assembly via adaptive k-mer weighting and repeat

separation. Genome research. 2017;27 5:722-36.

12. Ruan J and Li H. Fast and accurate long-read assembly with wtdbg2. Nature Methods. 2019:1-4.

13. Kajitani R, Toshimoto K, Noguchi H, Toyoda A, Ogura Y, Okuno M, et al. Efficient de novo assembly of highly heterozygous genomes from whole-genome shotgun short reads. Genome Res. 2014;24 8:1384-95. doi:10.1101/gr.170720.113.

14. Adema CM, Hillier LW, Jones CS, Loker ES, Knight M, Minx P, et al. Whole genome analysis of a schistosomiasis-transmitting freshwater snail. Nature communications. 2017;8:15451.

15. Liu C, Zhang Y, Ren Y, Wang H, Li S, Jiang F, et al. The genome of the golden apple snail Pomacea canaliculata provides insight into stress tolerance and invasive adaptation. GigaScience. 2018;7 9:giy101.

16. Biemont C. Genome size evolution: within-species variation in genome size. Nature Publishing Group, 2008.

17. Dehal P and Boore JL. Two rounds of whole genome duplication in the ancestral vertebrate. PLoS biology. 2005;3 10.

18. Bourque G, Burns KH, Gehring M, Gorbunova V, Seluanov A, Hammell M, et al. Ten things you should know about transposable elements. Genome biology. 2018;19 1:1-12.

19. Barth NK, Li L and Taher L. Independent transposon exaptation is a widespread mechanism of redundant enhancer evolution in the mammalian genome. Genome biology and evolution. 2020;12 3:1-17.

20. Li H and Durbin R. Inference of human population history from individual whole-genome sequences. Nature. 2011;475 7357:493-6.

21. Van Dover CL, Arnaud-Haond S, Gianni M, Helmreich S, Huber JA, Jaeckel A, et al. Scientific rationale and international obligations for protection of active hydrothermal vent ecosystems from deep-sea mining. Marine Policy. 2018;90:20-8.

22. Yang Z. PAML: a program package for phylogenetic analysis by maximum likelihood. CABIOS 13, 555–556. Supplementary material The following supplementary material is available for this article online: Fig S. 1997;1.

23. De Bie T, Cristianini N, Demuth JP and Hahn MW. CAFE: a computational tool for the study of gene family evolution. Bioinformatics. 2006;22 10:1269-71.

24. Sobieszczuk DF, Poliakov A, Xu Q and Wilkinson DG. A feedback loop mediated by degradation of an inhibitor is required to initiate neuronal differentiation. Genes & development. 2010;24 2:206-18.

25. Conductier G, Dusticier N, Lucas G, Côté F, Debonnel G, Daszuta A, et al. Adaptive changes in serotonin neurons of the raphe nuclei in 5-HT4 receptor knock-out mouse. European Journal of Neuroscience. 2006;24 4:1053-62.

26. Goffredi SK, Warén A, Orphan VJ, Van Dover CL and Vrijenhoek RC. Novel forms of structural integration between microbes and a hydrothermal vent gastropod from the Indian Ocean. Appl Environ Microbiol. 2004;70 5:3082-90.

27. Wolff T. Composition and endemism of the deep-sea hydrothermal vent fauna. CBM-Cahiers de Biologie Marine. 2005;46 2:97-104.

28. Farina AR, Tacconelli A, Cappabianca L, Masciulli MP, Holmgren A, Beckett GJ, et al. Thioredoxin alters the matrix metalloproteinase/tissue inhibitors of

metalloproteinase balance and stimulates human SK-N-SH neuroblastoma cell invasion. European Journal of Biochemistry. 2001;268 2:405-13.
29. Mukherjee A and Martin S. The thioredoxin system: a key target in tumour and endothelial cells. The British journal of radiology. 2008;81 special_issue_1:S57-S68.
30. Muri J, Heer S, Matsushita M, Pohlmeier L, Tortola L, Fuhrer T, et al. The thioredoxin-1 system is essential for fueling DNA synthesis during T-cell metabolic reprogramming and proliferation. Nature communications. 2018;9 1:1851.
31. Kato N, Dasgupta R, Smartt C and Christensen B. Glucosamine: fructose-6-phosphate aminotransferase: gene characterization, chitin biosynthesis and peritrophic matrix formation in Aedes aegypti. Insect molecular biology. 2002;11 3:207-16.
32. Lagorce A, Le Berre-Anton V, Aguilar-Uscanga B, Martin-Yken H, Dagkessamanskaia A and François J. Involvement of GFA1, which encodes glutamine–fructose-6-phosphate amidotransferase, in the activation of the chitin synthesis pathway in response to cell-wall defects in Saccharomyces cerevisiae. European journal of biochemistry. 2002;269 6:1697-707.
33. Csermely P, Schnaider T, So C, Prohászka Z and Nardai G. The 90-kDa molecular chaperone family: structure, function, and clinical applications. A comprehensive review. Pharmacology & therapeutics. 1998;79 2:129-68.
34. Marceau AH. Functions of single-strand DNA-binding proteins in DNA replication, recombination, and repair. Single-Stranded DNA Binding Proteins. Springer; 2012. p. 1-21.
35. Nazıroğlu M. Molecular role of catalase on oxidative stress-induced Ca2+ signaling and TRP cation channel activation in nervous system. Journal of Receptors and Signal Transduction. 2012;32 3:134-41.
36. Hohenester E, Sasaki T and Timpl R. Crystal structure of a scavenger receptor cysteine-rich domain sheds light on an ancient superfamily. Nature Structural & Molecular Biology. 1999;6 3:228.
37. Ligtenberg AJ, Karlsson NG and Veerman EC. Deleted in malignant brain tumors-1 protein (DMBT1): a pattern recognition receptor with multiple binding sites. International journal of molecular sciences. 2010;11 12:5212-33.
38. Aguilera F, McDougall C and Degnan BM. Co-option and de novo gene evolution underlie molluscan shell diversity. Molecular biology and evolution. 2017;34 4:779-92.
39. Mann K, Edsinger-Gonzales E and Mann M. In-depth proteomic analysis of a mollusc shell: acid-soluble and acid-insoluble matrix of the limpet Lottia gigantea. Proteome science. 2012;10 1:28.
40. Benoist CO, Mathis DJ, Kanter MR, Williams II VE and McDevitt HO. Regions of allelic hypervariability in the murine Aα immune response gene. Cell. 1983;34 1:169-77.
41. Lindberg DR PW, Haszprunar G. The Mollusca: Relationships and Patterns fromTheir First Half-Billion Years. Oxford: Oxford University Press; 2004.
42. Wanninger A and Wollesen T. The evolution of molluscs. Biological Reviews.

2019;94 1:102-15.
43. Vinther J. A molecular palaeobiological perspective on aculiferan evolution. Journal of natural history. 2014;48 45-48:2805-23.
44. Vinther J. The origins of molluscs. Palaeontology. 2015;58 1:19-34.
45. Lee H, Chen W, Puillandre N, Aznarcormano L, Tsai M and Samadi S. Incorporation of deep-sea and small-sized species provides new insights into gastropods phylogeny. Molecular Phylogenetics and Evolution. 2019;135:136-47.
46. Kazazian HH. Mobile elements: drivers of genome evolution. science. 2004;303 5664:1626-32.
47. Sundaram V, Cheng Y, Ma Z, Li D, Xing X, Edge P, et al. Widespread contribution of transposable elements to the innovation of gene regulatory networks. Genome research. 2014;24 12:1963-76.
48. Jacques P-E, Jeyakani J and Bourque G. The majority of primate-specific regulatory sequences are derived from transposable elements. PLoS genetics. 2013;9 5.
49. Janoušek V, Laukaitis CM, Yanchukov A and Karn RC. The role of retrotransposons in gene family expansions in the human and mouse genomes. Genome biology and evolution. 2016;8 9:2632-50.
50. Drmanac R, Sparks AB, Callow MJ, Halpern AL, Burns NL, Kermani BG, et al. Human genome sequencing using unchained base reads on self-assembling DNA nanoarrays. Science. 2010;327 5961:78-81. doi:10.1126/science.1181498.
51. Huang J, Liang X, Xuan Y, Geng C, Li Y, Lu H, Qu S, Mei X, Chen H, Yu T, Sun N, Rao J, Wang J, Zhang W, Chen Y, Liao S, Jiang H, Liu X, Yang Z, Mu F, Gao S. 2018. BGISEQ-500 Sequencing. protocols.io https://dx.doi.org/10.17504/protocols.io.pq7dmzn
52. Rao SS, Huntley MH, Durand NC, Stamenova EK, Bochkov ID, Robinson JT, et al. A 3D map of the human genome at kilobase resolution reveals principles of chromatin looping. Cell. 2014;159 7:1665-80. doi:10.1016/j.cell.2014.11.021.
53. Servant N, Varoquaux N, Lajoie BR, Viara E, Chen CJ, Vert JP, et al. HiC-Pro: an optimized and flexible pipeline for Hi-C data processing. Genome Biol. 2015;16:259. doi:10.1186/s13059-015-0831-x.
54. Dudchenko O, Batra SS, Omer AD, Nyquist SK, Hoeger M, Durand NC, et al. De novo assembly of the Aedes aegypti genome using Hi-C yields chromosome-length scaffolds. Science. 2017;356 6333:92-5. doi:10.1126/science.aal3327.
55. Durand NC, Shamim MS, Machol I, Rao SS, Huntley MH, Lander ES, et al. Juicer Provides a One-Click System for Analyzing Loop-Resolution Hi-C Experiments. Cell Syst. 2016;3 1:95-8. doi:10.1016/j.cels.2016.07.002.
56. Durand NC, Robinson JT, Shamim MS, Machol I, Mesirov JP, Lander ES, et al. Juicebox Provides a Visualization System for Hi-C Contact Maps with Unlimited Zoom. Cell Syst. 2016;3 1:99-101. doi:10.1016/j.cels.2015.07.012.
57. Tarailo-Graovac M and Chen N. Using RepeatMasker to identify repetitive elements in genomic sequences. Current Protocols in Bioinformatics. 2009:4.10. 1-4.. 4.
58. Jurka J, Kapitonov VV, Pavlicek A, Klonowski P, Kohany O and Walichiewicz J. Repbase Update, a database of eukaryotic repetitive elements. Cytogenetic and genome research. 2005;110 1-4:462-7.

59. Xu Z and Wang H. LTR_FINDER: an efficient tool for the prediction of full-length LTR retrotransposons. Nucleic acids research. 2007;35 suppl 2:W265-W8.
60. Benson G. Tandem repeats finder: a program to analyze DNA sequences. Nucleic acids research. 1999;27 2:573.
61. She R, Chu JS, Wang K, Pei J and Chen N. GenBlastA: enabling BLAST to identify homologous gene sequences. Genome Res. 2009;19 1:143-9. doi:10.1101/gr.082081.108.
62. Birney E, Clamp M and Durbin R. GeneWise and Genomewise. Genome Res. 2004;14 5:988-95. doi:10.1101/gr.1865504.
63. Keller O, Kollmar M, Stanke M and Waack S. A novel hybrid gene prediction method employing protein multiple sequence alignments. Bioinformatics. 2011;27 6:757-63. doi:10.1093/bioinformatics/btr010.
64. UniProt C. UniProt: a worldwide hub of protein knowledge. Nucleic Acids Res. 2019;47 D1:D506-D15. doi:10.1093/nar/gky1049.
65. Elsik CG, Mackey AJ, Reese JT, Milshina NV, Roos DS and Weinstock GM. Creating a honey bee consensus gene set. Genome biology. 2007;8 1:1.
66. Boeckmann B, Bairoch A, Apweiler R, Blatter M-C, Estreicher A, Gasteiger E, et al. The SWISS-PROT protein knowledgebase and its supplement TrEMBL in 2003. Nucleic acids research. 2003;31 1:365-70.
67. Kanehisa M and Goto S. KEGG: kyoto encyclopedia of genes and genomes. Nucleic acids research. 2000;28 1:27-30.
68. Apweiler R, Attwood TK, Bairoch A, Bateman A, Birney E, Biswas M, et al. The InterPro database, an integrated documentation resource for protein families, domains and functional sites. Nucleic acids research. 2001;29 1:37-40.
69. Li H, Coghlan A, Ruan J, Coin LJ, Heriche JK, Osmotherly L, et al. TreeFam: a curated database of phylogenetic trees of animal gene families. Nucleic Acids Res. 2006;34 Database issue:D572-80. doi:10.1093/nar/gkj118.
70. Edgar RC. MUSCLE: multiple sequence alignment with high accuracy and high throughput. Nucleic Acids Res. 2004;32 5:1792-7. doi:10.1093/nar/gkh340.
71. Yang Z. PAML 4: phylogenetic analysis by maximum likelihood. Mol Biol Evol. 2007;24 8:1586-91. doi:10.1093/molbev/msm088.
72. Yang Z and Rannala B. Bayesian estimation of species divergence times under a molecular clock using multiple fossil calibrations with soft bounds. Mol Biol Evol. 2006;23 1:212-26. doi:10.1093/molbev/msj024.
73. Li H and Durbin R. Fast and accurate long-read alignment with Burrows-Wheeler transform. Bioinformatics. 2010;26 5:589-95. doi:10.1093/bioinformatics/btp698.
74. Guindon S, Delsuc F, Dufayard J-F and Gascuel O. Estimating maximum likelihood phylogenies with PhyML. Bioinformatics for DNA sequence analysis. 2009:113-37.
75. Van der Auwera GA, Carneiro MO, Hartl C, Poplin R, Del Angel G, Levy-Moonshine A, et al. From FastQ data to high confidence variant calls: the Genome Analysis Toolkit best practices pipeline. Curr Protoc Bioinformatics. 2013;43:11 0 1-33. doi:10.1002/0471250953.bi1110s43.
76. Li H and Durbin R. Inference of human population history from individual whole-genome sequences. Nature. 2011;475 7357:493-6. doi:10.1038/nature10231.
77. Warren WC, Kuderna L, Alexander A, Catchen J, Perez-Silva JG, Lopez-Otin C, et

al. The Novel Evolution of the Sperm Whale Genome. Genome Biol Evol. 2017;9 12:3260-4. doi:10.1093/gbe/evx187.

78. Schilthuizen M. Rapid, habitat-related evolution of land snail colour morphs on reclaimed land. Heredity. 2013;110 3:247-52.

79. Price MN, Dehal PS and Arkin AP. FastTree 2–approximately maximum-likelihood trees for large alignments. PloS one. 2010;5 3:e9490.

80. Zeng X; Zhang Y; Meng L; Fan G; Bai J; Chen J; Song Y; Seim I; Wang C; Shao Z; Liu N; Lu H; Fu X; Wang L; Liu X; Liu S; Shao Z. Supporting data for "Genome sequencing of deep-sea hydrothermal vent snails reveals adaptions to extreme environments" GigaScience Database 2020. http://dx.doi.org/10.5524/100817.

Fig 1

Click here to access/download;Figure;Figure 1.pdf

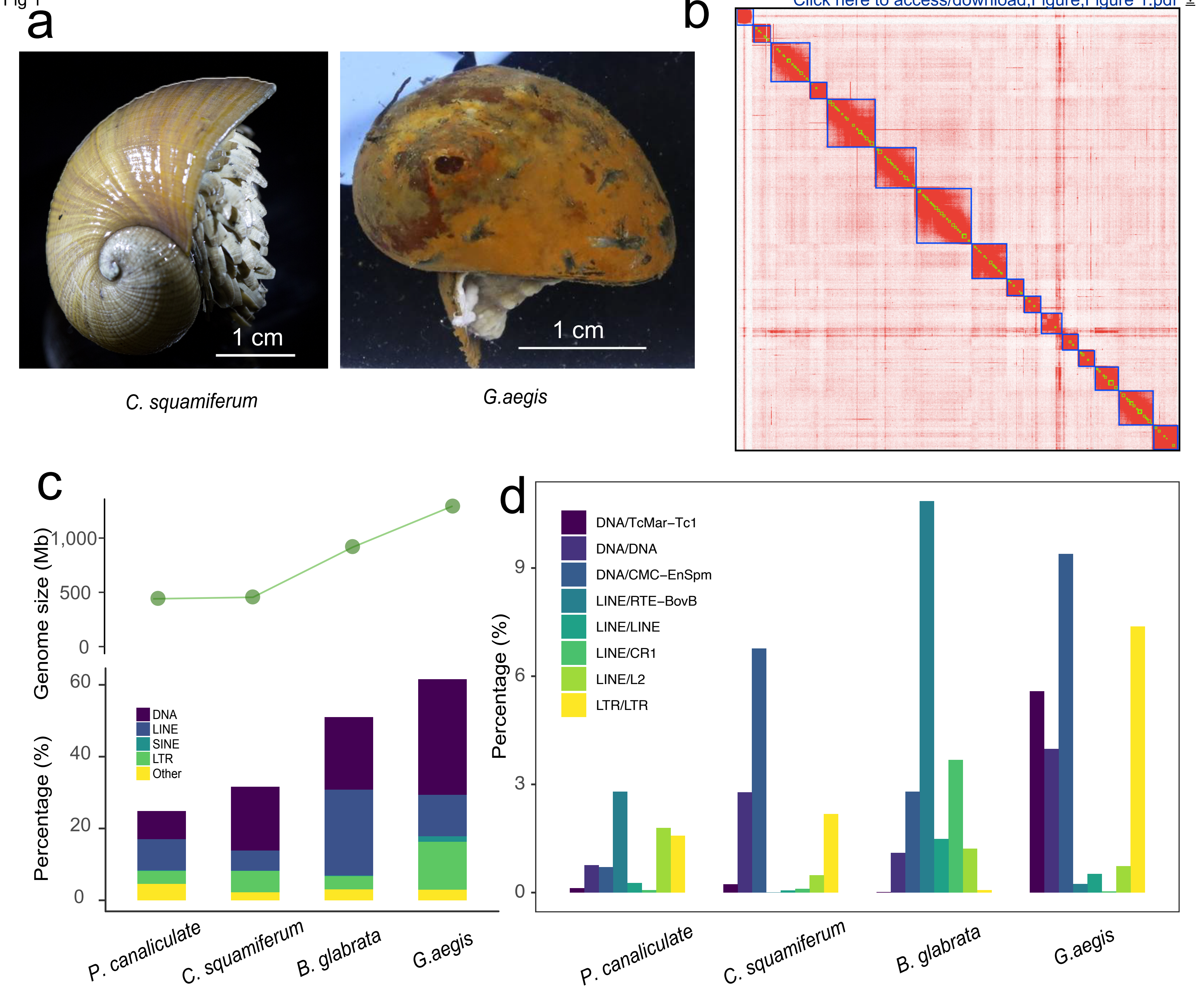

Fig 2

Click here to access/download;Figure;Figure 2.pdf

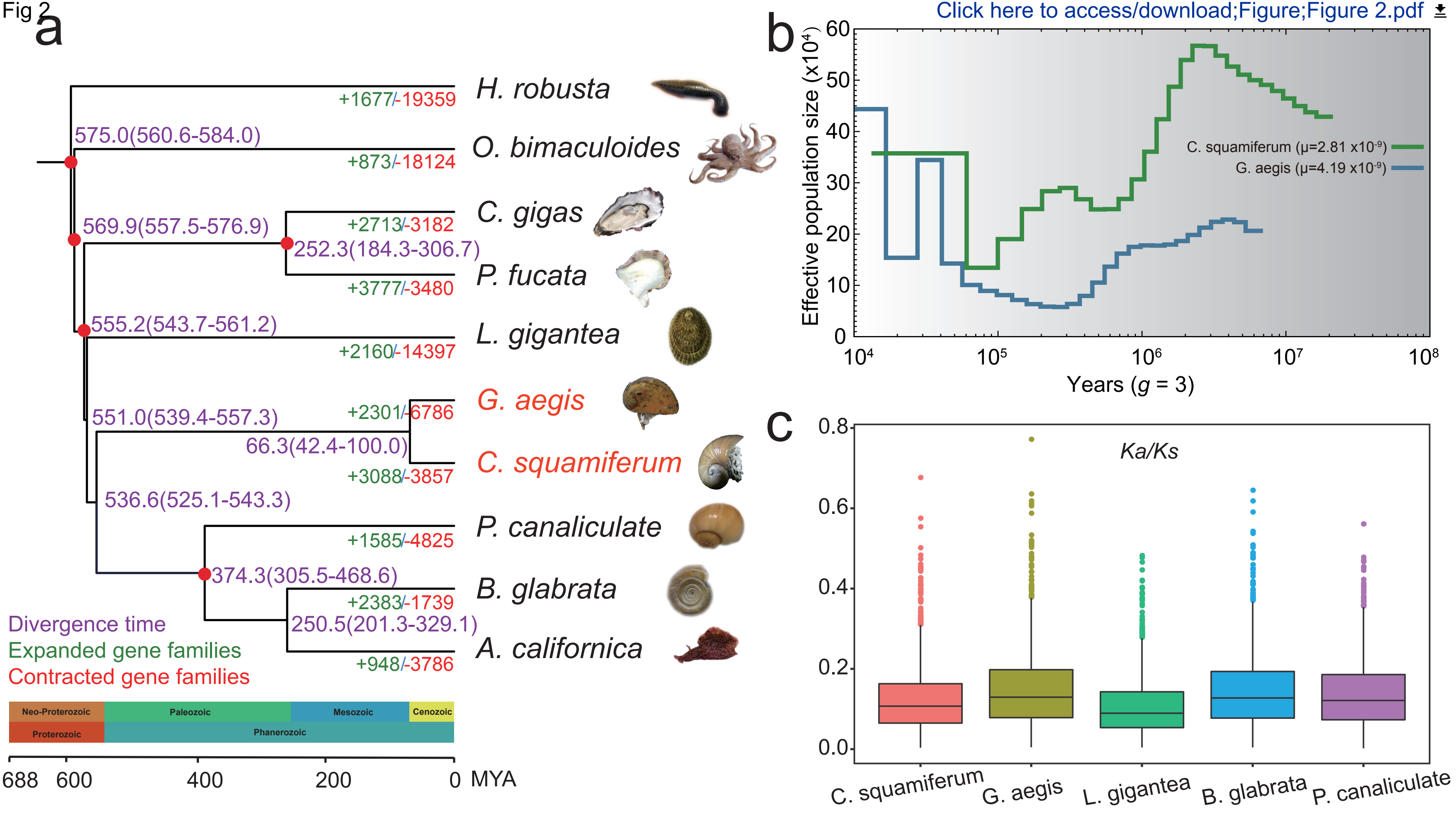

Fig 3

Click here to access/download;Figure;Figure 3.pdf

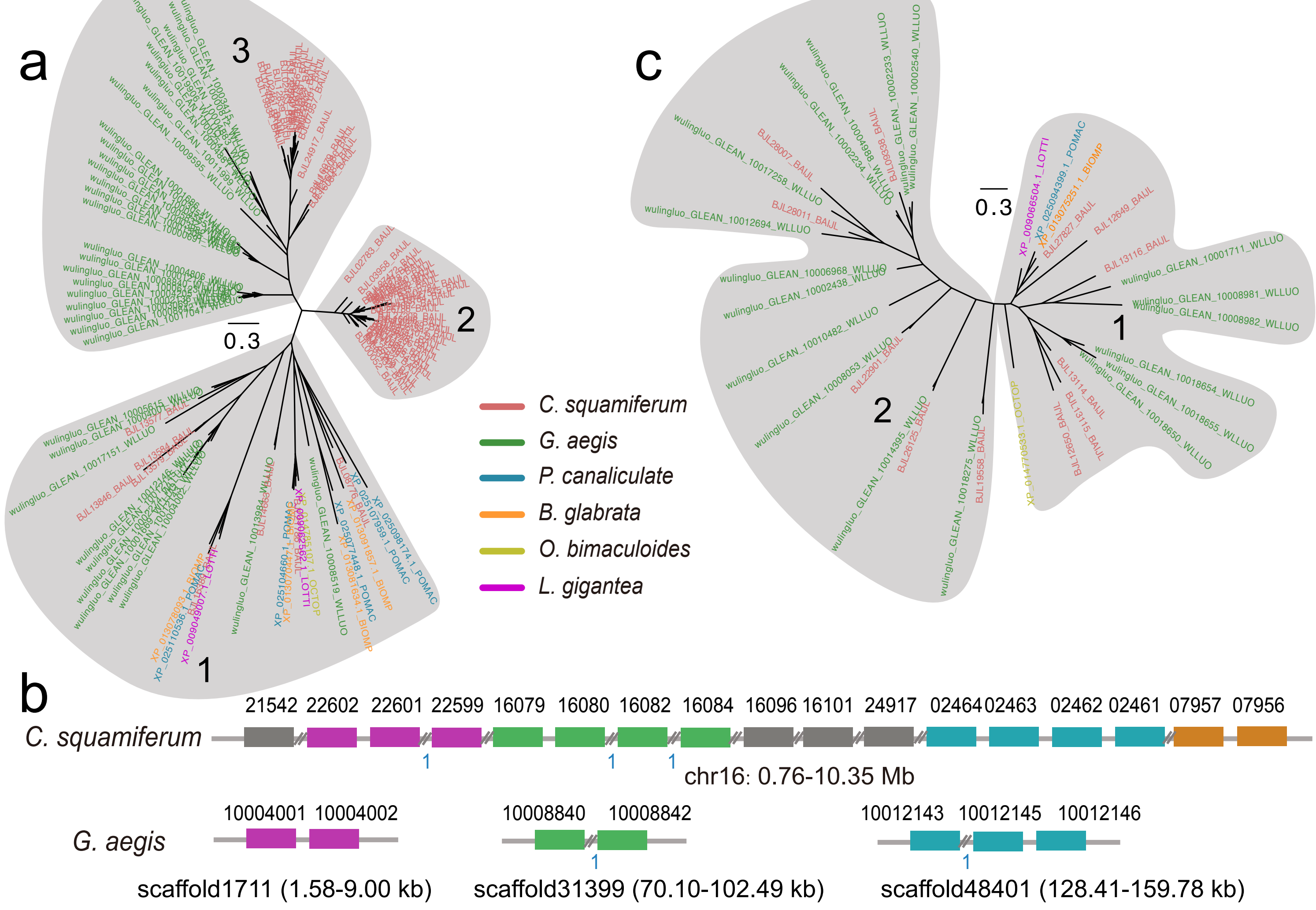

Fig 4

Click here to access/download;Figure;Figure 4.pdf

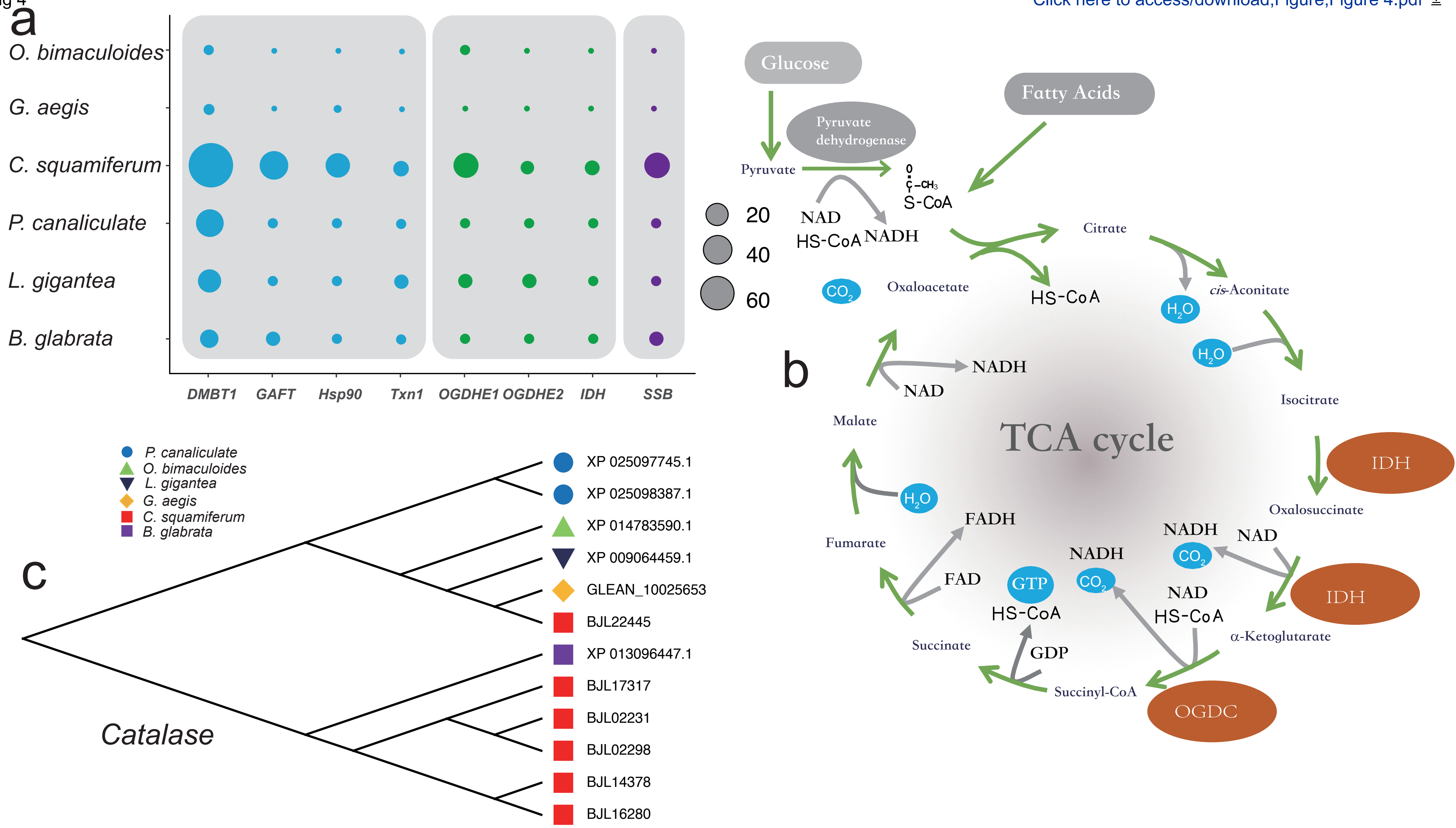

Click here to access/download
Supplementary Material
Supplementary Figures and Tables-revision_2.docx

Point to point response letter
Click here to access/download;Personal Cover;PointToPointResponseLetter.docx

Response to editors and reviewers:

Dear editor and reviewers:
On behalf of all the coauthors, we would like to thank you very much and all the reviewers for the time spent to assess our manuscript (GIGA-D-20-00187) and for your relevant remarks and suggestions that allow to improve the quality of this manuscript.
We have checked the manuscript carefully and revised it according to the comments.

Sincerely,
Xiang Zeng, Yaolei Zhang

Reviewer reports:
Reviewer #1: The Data Note by Zeng et al. reported two genome assemblies of deep sea gastropods, Chrysomallon squamiferum and Gigantopelta aegis.
I would ask the authors for additional informations about assembling process and references of data sources in order to guarantee the quality of the data and analyses. I also found there are many ambiguous expressions in the present manuscript, making it unclear how the genome resources can contribute to understand biology of these animals.
Please find my specific comments and concerns below, which need to be addressed.
Response: Thank you very much for your thoughtful and helpful suggestions. We have revised our manuscript according to your comments. Please see that.

Background
The authors should mention the fact that the Chrysomallon squamiferum genome has been published by Sun et al. (Nat Commun 11, 1657, 2020) somewhere in the Background section.
I would suggest the authors to explain that they analyzed the genome of "white scaly foot individual" while Sun et al. sequenced "black" one, to emphasize the uniqueness of this study.
Response: Thank you very much for this helpful suggestion. We have added descriptions as you suggested. Please see **lines 91-93** “And the whole genome of black scaly-foot snail was reported recently, which highlighted its evolutionary mechanisms of biomineralised armour [9]” and 109-110: “In this study, we sequenced and assembled genomes of the white scaly-foot snail (Figure 1a), which is different from the published black individual”.

Line 93
Remove "sp. nov.". This abbreviation is used when new species is named.
Response: Thank you. We removed this abbreviation. See **lines 94-96**: “The genus includes two species, *Gigantopelta chessoia* from East Scotia Ridge and *Gigantopelta aegis* from the Southwest Indian Ridge [6]. Both *Chrysomallon* and *Gigantopelta* are members of the family Peltospiridae.”

Data description

Line 110

Was the insert size 350bp (main text) or 300bp (Table S2)?

Response: Thank you very much. We modified it as “350 bp” in Table S2.

Line 121-122

As mentioned above, the *C. squamiferum* genome has been published. Therefore this sentence needs to be removed.

Response: Thank you. We delete this sentence.

Line 156-160

These sentences do not make sense to me. Why despite "precise functions of these repeats have not been studied," the authors can infer the composition of repeat elements "may be closely associated with adaptation to extreme environment"? Please describe more specifically by mentioning some references that support this idea.

Response: Thank you and sorry for confusing you. We added more description and relevant references about the importance of transposons/repeats. Please see **lines: 156-161** “Although most of the precise functions of these repeats have not been studied in depth, repeats have been thought to have a regulatory function in related genes that play an important role in the life cycle and can introduce great genome flexibility [18]. And in the mammalian genome, transposons were described to be redundant enhancers that regulate their target genes which are higher or tissue specially expressed, indicating the importance of transposons”

Lines 173-175

It seems the authors assumed the split of C. squamiferum and G. aegis was related to the mass extinction event around 66 MYA. However, estimated divergence time is considerably ambiguous (42.4-100 MYA, Fig 1a), making the idea less reliable.

Response: Thank you. We agree with you and delete this description.

Deleted sentences: “This time is consistent with the most recent ‘mass extinction’, at the end of the Cretaceous geological period ~66 MYA, where ~76% of species became extinct” for your reference.

Lines 178-180

This sentence is difficult to understand. Speciation and demographic histories of each species are different topics.

Response: Thank you. We also delete this sentence.

Deleted sentences: “As the speciation of the two deep-sea snails may be related to geological events (see above)” for your reference.

Lines 190-191
This sentence is not clear. Please describe what "major geological events" affected the population sizes, with references describing the geological events.

An: Thank you. We added one example of geological events and relevant reference. Please see **lines 194-197**: “For example, the Cretaceous/Paleogene (K/Pg) extinction event caused extinction of three-quarters of species on earth and affected population dynamics approximately 66 million years ago, when an asteroid impact caused global environmental devastation [21, 22]”

Lines 191-193
The recent decreased population size was reported by ref[8] and was not related to this study (Fig2b). Then, this sentence may be put in the Background section.
An: Thank you. We deleted this sentence.

Deleted sentences: ” Unfortunately, the C. squamiferum population size has dramatically decreased recently due to deep-sea mining [8], which has made this species endangered” just for your reference

Lines 196-210
Comparisons of Ks and Ka values among snail species should be tested statistically. In the figures 2C and S3 it is not clear whether these values are significantly different.
Response: Thank you. We have added statistical test (Mann-Whitney U test) for Ka/Ks comparisons. Please see **lines 204-213**: “We found that the Ka values of the two deep-sea snails (average: 0.37 and 0.41) were higher (Mann-Whitney U test, p-value<0.001) than that of the shallow-water limpet (0.35) but similar to those of two freshwater snails (0.39 and 0.41), which suggests that the genes of deep-sea and freshwater snails both evolved faster after their divergence from shallow-water limpet . The Ks values of the deep-sea (3.34 and 3.09) and freshwater (3.19 and 3.24) snails were also similar and lower (Mann-Whitney U test, p-value<0.001) than those of the shallow-water limpet (3.72). Additionally, the Ka/Ks values of the deep-sea snails (average: 0.13 and 0.15) were approximately ~20% and ~40% higher (Mann-Whitney U test, p-value<0.001) than those of the shallow-water limpet (0.11)”.

Discussion
Lines 309-310
I have no idea what the "infamous Cambrian Explosion" means. Please explain the authors' idea more in detail.
Response: Thank you. It was modified to “Cambrian Explosion”. It was a clerical error. See **line 319**.

Lines 328-329
Please describe what are "adaptive needs" and "region-specific features" specifically.

Response: Thank you. We deleted this sentence.

Materials and Methods
Line 370
350bp or 300bp?
Response: It is “350 bp” actually for BGISEQ.

Lines 403-405
Based on the description, the 10X Chromium reads were used only for polishing, not for scaffolding. On the other hand, there are stats of scaffolds before Hi-C scaffolding in Table S3.
Response: Yes, the 10X Chromium reads were used only for polishing, not for scaffolding.

My questions are;
i) Were the scaffolds in Table S3 generated using 10X Chromium reads?
ii) If so, the scaffolds were improved very little (sequences are reduced from 6449 to 6444), indicating there was problem in 10X Chromium sequencing. How the authors interpreted the results?
Response: Thank you. The 6,444 scaffolds (also 6,444 contigs) were generated using Oxford Nanopore reads. We then used 10X Chromium reads to polish (error correction) 6,444 scaffolds with software Pilon. Pilon introduced 5 bp gaps so the contig number became 6,449 while the scaffold number was still 6,444.

Line 436
"Lottia"
Response: Thank you. We corrected this word.

Line 454
Describe a reference for GLEAN.
Response: Thank you. We added a reference for GLEAN. Please see **line 465**.

Lines 464-467
Describe sources or references for these genomic data.
Response: Thank you. We added sources for these genomic data. Please see **lines 474-479**: “ all the protein sequences from selected 10 representative species (8 species including Aplysia californica (GCF_000002075.1), Octopus bimaculoides (GCF_001194135.1), Biomphalaria glabrata (GCF_000457365.1), Crassostrea gigas (GCF_000297895.1), Lottia gigantea (GCF_000327385.1), Pomacea canaliculate (GCF_003073045.1), Pinctada fucata (GCA_002216045.1), Helobdella robusta (GCF_000326865.1) from NCBI database, C. squamiferum and G. aegis from this research) were compared using blastp with the E-value threshold set as 1e-7.”

Lines 484-487

Describe references of these fossil records rather than summary database (Timetree.org) so that readers can refer the original data.

Response: Thank you very much and we can't agree with you more. However, each of the time point between two species refers a lot of references and TimeTree database summarized all of these references to estimate one divergence time with a confidence interval. For example, the divergence time between *Aplysia californica* and *Crassostrea gigas* was estimated to be 537 MYA with a confidence interval of 516.3-558.3 MYA based on 11 references. We used this confidence interval time to calibrate our estimation. This is a common method used frequently in nowadays genome research. So here we cite TimeTree database (Timetree.org) for reference which includes many references. However, if you think list all the references is a must, we are pleased to do this.

Methods of SNP identification and PSMC (lines 177-193) were not described.

Response: Thank you. We have added methods of SNP identification and PSMC. Please see **lines 503-516**: "SNP calling and estimation of history population sizes About 50X clean WGS reads were mapped to genomes of C. squamiferum and G. aegis using BWA mem (v0.7.12-r1039) [73] with default parameters respecitvely. Then SAMtools (v0.1.19-44428cd) [74] and "SortSam.jar" in the picard package (v1.54) was used to convert and sort BAM files. Local realignment was again carried out using RealignerTargetCreator and IndelRealigner in GATK (v3.6) [75] with default parameters. SNPs were identified using HaplotypeCaller and filtered using VariantFiltration with parameter "-filter-expression "QD < 2.0 || MQ < 40.0 || ReadPosRankSum < -8.0 || FS > 60.0" --filter-name LowQualFilter --genotype-filter-expression "DP < 5.0" --genotype-filter-name lt_5". Estimatation of history population sizes were carried out using PSMC (v0.6.5-r67) [76]. Firstly, diploid genome references were constructed using samtools and bcftools call with "samtools mpileup -C50" and "vcfutils.pl vcf2fq -d 20 -D 100". Secondly, the demographic history was inferred using PSMC with parameters '-N25 -t15 -r5 -p 4+25*2+4+6' [77]."

Fig 1a

Add the size of the scale for C. squamiferum. No scale is indicated for G.aegis.

Response: Thank you. Scale was added in **Fig 1a**. Also, we added "Scale bar = 1cm" in **Fig 1** legend.

Fig 2a

This cartoon is too ambiguous and not suitable for scientific paper. The molecular phylogeny should be clearly shown by solid lines.

Response: Thank you. We have updated **Fig. 2a**. Please see that.

Fig.3d

This figure is not very informative for readers. The authors may want to draw molecular phylogeny trees for BTBD6 and HTR4.

Response: Thank you. We also deleted **Fig.3d**.

Reviewer #2: The manuscript entitled "Genome sequencing of deep-sea hydrothermal vent snails reveals adaptations to extreme environments" presents a nice description of a good genome assembly (16 chromosomes representing ~80% of the genome) of the scaly foot snail (Chrysomallon squamiferum) and compare it to genomes of other molluscan species. Overall the paper is well written and presents a nice view of some unique adaptations by this deep-sea mollusc. One concern that I had is throughout the manuscript (starting at line 164 and onward) the authors describe comparing two mussels, two freshwater snails and two shallow-water snails to their genomes. However, these other molluscan species include C. gigas and P. fucata…which are both oysters and not mussels, and while two of the other molluscs included in the tree are in Gastropoda and considered snails, Lottia gigantea is a limpet and Aplysia californica is a sea slug. I would encourage the authors to describe all of these species more accurately, i.e., as limpet and sea slug, because these are very different from what people commonly think of when they hear "snail", represented by the more traditional Pomacea and Biomphalaria. Referring to all the "snails" as gastropods would be a more suitable term that captures the true diversity of this large group. But when discussing individual species, I would prefer to see the more accurate descriptions because limpets and sea slugs are different from traditional snails, and will have unique adaptations of their own related to their unique characteristics. Overall, the authors give a good general description of the results and present a reasonable discussion about some of the potential adaptations that they observed in the genome. One minor point - thioredoxins are much more likely play a role in repairing proteins that have been altered by oxidation (Lines 255-256), so to limit this expansion to innate immunity leaves out a lot of other possibilities. My other question was regarding the source of the genomic DNA. The authors describe using muscle samples for isolating DNA, but it is not clear if DNA from one individual was used for all sequencing or if pooling occurred?
Response: Thank you very much for your approval and your thoughtful advices. We have updated our descriptions based on your suggestions. Please see below response.

1) About the scientific name (line 164 and onward), we modified this part as "we compared them with two shallow-water bivalves (P.fucata and C.gigas) and four shallow-water gastropods, including two fresh-water snails (B. glabrata and P. canaliculate), one limpet (B. glabrata) and one sea slug (A. californica). The California two-spot octopus (O. bimaculoides) and the freshwater leech (H.robusta) were used as the outgroup (**Figure 2a**) (**lines 167-171**)."
2) About thioredoxins, we added the description of thioredoxin as redox proteins and references. Please **lines 260-265**: "For example, increased expression of thioredoxin 1 (Txn1; 22 copies in C. squamiferum) was identified. Thioredoxin 1 (Txn1), a redox protein, is important in regulation of cellular redox homeostasis and anti-apoptotic functions. Txn1 stimulates cell proliferation and cell cycle progression, induces

hypoxia-inducible factor-1α (HIF-1α) and angiogenesis, and alters the balance between the matrix metalloproteinases and their tissue inhibitors [29, 30]"
3) About isolating DNA, all DNA was isolated from one individual and we add clear description about this in **lines 374-375**: "DNA was extracted from muscle sample of one individual using the cetyl trimethylammonium bromide (CTAB) method and a DNeasy blood & tissue kit (QIAGEN)."

Reviewer #3: The manuscript of Zeng et al seems to describe a well-put together genome for one species of deep-sea snail, with an additional 'draft' genome for another species. It is clear and well-written, with most of the methods described sufficiently. My main criticism is that I found some of the discussions regarding the adaptative significance and/or putative "function" of various TE content and gene-family expansion results quite speculative, given that the comparative results are often observational with no hypothesis testing or statistical framework. That may well be beyond the remit of the paper, but the language could be more careful in places to reflect the putative nature of any hypothesised effects. Nonetheless I have no doubt that the genomes themselves will be useful additions to the community for future work on mollusc and animal evolution.
Response: Thank you very much for your approval of our manuscript and your helpful criticism. Yes, you are quite right. Here we did not show any experiments results to valid our hypothesis or speculation because this is a data description paper, mainly focusing on observational data. More investigations including both in-depth analysis and experiments based on these two genomes and these findings will be carried out in the future to verify function of important genes or TEs or conserved no-coding regions. These are important and interesting issues and must be done then. Thank you again.

Minor comments:
- Typo line 38: "impedes"
Response: Thank you. This word was modified

- Line 158: is there a reference or two for this? I would assume that most TEs are simply selfish genetic elements that do not serve a "function" per se but exist only for their own purpose, i.e. to copy themselves independently of the host genome
- Line 158-160: but most TE content differences are probably driven by stochastic forces (i.e. drift) rather than deterministic forces such as adaptation, and here we have only 2 data points. The language used for this statement is careful, but I wonder if it is too far to extrapolate that some differences in TE content may be adaptive
Response: Thank you very much for your helpful thoughts. We added more descriptions and references to support "TEs are functional" to make it clear. Please see **lines 156-161**: "Although most of the precise functions of these repeats have not

been studied in depth, repeats have been thought to have a regulatory function in related genes that play an important role in the life cycle and can introduce great genome flexibility[18]. And in the mammalian genome, transposons were described to be redundant enhancers that regulate their target genes which are higher or tissue specially expressed, indicating the importance of transposon".

- Line 196: I don't know what the authors mean by this statement

Response: Thank you. We deleted the sentence.

Deleted sentence "The evolution and expression of single-copy orthologous genes are unique features of organisms." for your reference.

- Section on Ka/Ks values: there is no impression given about the statistical significance of the differences observed between Ka/Ks in any given lineage, or what the distribution of error looks like for these point estimates. Perhaps a more refined PAML analysis could resolve this? It is also not written how Ka/Ks values were calculated

Response: Thank you. We have added statistical test (Mann-Whitney U test) for Ka/Ks comparisons. Please see **lines 204-213**: "We found that the Ka values of the two deep-sea snails (average: 0.37 and 0.41) were higher (Mann-Whitney U test, p-value<0.001) than that of the shallow-water limpet (0.35) but similar to those of two freshwater snails (0.39 and 0.41), which suggests that the genes of deep-sea and freshwater snails both evolved faster after their divergence from shallow-water limpet . The Ks values of the deep-sea (3.34 and 3.09) and fresh-water (3.19 and 3.24) snails were also similar and lower (Mann-Whitney U test, p-value<0.001) than those of the shallow-water limpet (3.72). Additionally, the Ka/Ks values of the deep-sea snails (average: 0.13 and 0.15) were approximately ~20% and ~40% higher (Mann-Whitney U test, p-value<0.001) than those of the shallow-water limpet (0.11).

And the Ka/Ks values were calculated actually using codeml in PAML package. We have added this information to **lines 200-204**: "To explore the evolutionary rate of single-copy orthologous genes, we calculated the synonymous substitution rate (Ka) and nonsynonymous substitution rate (Ks) values of 1,324 single-copy orthologous genes shared by the two deep-sea snails, one shallow-water limpet (L. gigantea), and two freshwater snails (B. glabrata and P. canaliculate) using Codeml in PAML package[23]."

- Typo line 214: CAFE not CAFÉ

Response: Thank you. It was modified.

- Line 329: "region-specific feature shared between lineages" - not sure what is meant by this?

Response: Thank you. We deleted this confusing sentence.

- Line 350: it seems speculative - surely both immune response and biomineralization are "vital" for all snails, not particularly deep-sea ones?

Response: Thank you. We modified it and specified it in *C. squamiferum* which is a chemosynthetic snail species depending on endosymbionts. See **lines 360-362** “In particular, we found that DMBT1 gene families that encode multiple SRCR domains expanded significantly in C. squamiferum. These genes play important roles in immune response and biomineralization, both of which are vital for deep sea chemosynthetic snail”.

- Line 454: reference for GLEAN is missing

Response: Thank you. We added this reference (65). See **line 465**

- Line 469: references for Solar and Hcluster are missing, and what is a H-score?

Response: Thank you. Solar, Hcluster_sg and H-score are tools and concept of TreeFam tools. We added reference for Solar and Hcluster_sg. H-score means hcluster score. Details can be found from TreeFam tools.

- Figure 2a: it's a weird looking tree that, in fact, looks a bit like a snail itself! Are the widths of the blobs representative of the error around the divergence times or topological support?

Response: Thank you and sorry for confusing you. We updated **Fig.2a**. Please see that.
