# Supplementary material for: Genome sequencing of deep-sea hydrothermal vent snails reveals adaptions to extreme environments
Source: Gigascience. 2020 Dec 15;9(12):giaa139. doi: 10.1093/gigascience/giaa139 (PMC7736800; doi:10.1093/gigascience/giaa139)
Supplement: giaa139_Supplemental_Tables_and_Figures [file giaa139_supplemental_tables_and_figures.docx]

**Supplemental information for:**

***Genome sequencing of deep-sea hydrothermal vent snails reveals adaptions to extreme environments***

**Supplementary Figures:**

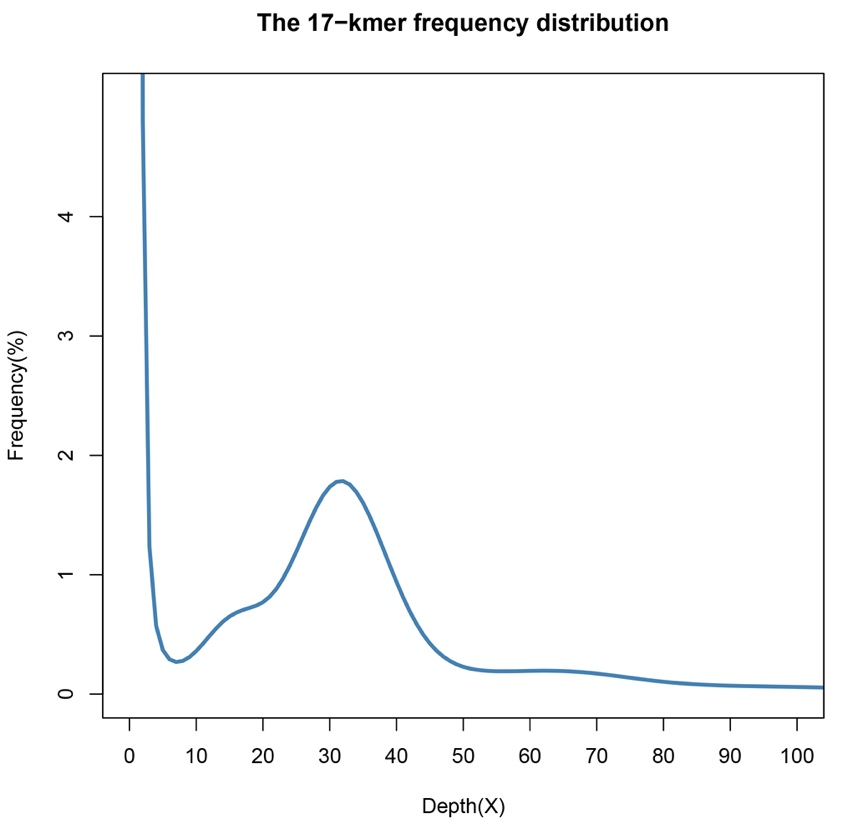


**Figure S1.** 17-mer frequency distribution for *C. squamiferum and* *G. aegis* genomes. Estimated genome sizes were 495 Mb and 1.498 Gb, respectively.

**Figure S2**. Construction of Phylogenetic trees for ten representative molluscs using coding sequences of 407 single-copy orthologs. **a)** ML method with GTR+gamma model. **b**) Bayesian method with the GTR+gamma model.

**Figure S3**. Box plot of *K*a and *K*s values of 1,324 single copy orthologous genes from two deep-sea snails, one shallow-water snail, and two fresh-water snails.

**Figure S4**. Expansion pattern of *HTR4* genes in two deep-sea snails. Grey lines represent scaffolds sequences. Colour rectangles represent BTBD6 genes. The symbols, “//”, indicate that there are other genes along the scaffolds. The blue numbers, e.g. “1”, indicate that there is only one gene between the tandem duplicated genes.

**Figure S5**. KEGG enrichment analysis of unique gene families of *G. aegis.*

**Supplementary Tables:**

**Table S1.** Statistics of raw sequencing data of *Chrysomallon squamiferum*

| **Sequencing platforms** | **Libraries** | **Average Reads** | **Total** |
| --- | --- | --- | --- |
|  |  | **Length (bp)** | **Data (Gb)** |
| BGISEQ-500 | 10x Genomics | 150 | 173.27 |
| GridION X5 | ONT | 2,516 | 39.62 |
| BGISEQ-500 | Hi-C | 100 | 156.14 |
| Total | \ | \ | 369.03 |

**Table S2.** Statistics of raw sequencing data of ***Gigantopelta aegis***

| **Sequencing platforms** | **Insert**  **Sizes of WGS Libraries** | **Paired-end Reads**  **Length (bp)** | **Total**  **Data (Gb)** |
| --- | --- | --- | --- |
| BGISEQ-500 | 350bp | 100_100 | 499.04 |
|  | 2kb | 50_50 | 133.18 |
|  | 5kb | 50-50 | 137.47 |
|  | 10kb | 50-50 | 140.39 |
| Total | \ | \ | 910.08 |

**Table S3.** Summary from the genome assembly of *Chrysomallon squamiferum* without using Hi-C data

|  | Scaffold | Contig |
| --- | --- | --- |
| Sequence number (>): | 6,444 | 6,449 |
| Total length of (bp): | 454,575,842 | 454,575,837 |
| Gap number (bp): | 5 | 0 |
| Average length (bp): | 70,542 | 70,488 |
| N50 Length (bp): | 545,970 | 541,320 |
| N90 Length (bp): | 21,261 | 21,258 |
| Maximum length (bp): | 3,781,429 | 3,781,429 |
| GC content is (%): | 34.48 | 34.48 |

**Table S4.** Lengths of the 16 chromosomes assembled for *Chrysomallon squamiferum.*

| Chromosome ID | Length (bp) |
| --- | --- |
| chr1 | 46,776,756 |
| chr2 | 40,299,015 |
| chr3 | 33,910,444 |
| chr4 | 33,112,422 |
| chr5 | 29,179,595 |
| chr6 | 28,850,124 |
| chr7 | 20,709,717 |
| chr8 | 20,213,658 |
| chr9 | 14,637,985 |
| chr10 | 14,513,379 |
| chr11 | 14,354,400 |
| chr12 | 14,190,952 |
| chr13 | 14,411,729 |
| chr14 | 13,760,285 |
| chr15 | 13,138,974 |
| chr16 | 10,640,320 |

**Table S5.** BUSCO assessment of the assembled genome of *Chrysomallon squamiferum* using metazoa_odb9 database

| \ | Gene number | Percentage (%) |
| --- | --- | --- |
| Complete BUSCOs (C) | 927 | 94.8 |
| Complete and single-copy BUSCOs (S) | 888 | 90.8 |
| Complete and duplicated BUSCOs (D) | 39 | 4.0 |
| Fragmented BUSCOs (F) | 16 | 1.6 |
| Missing BUSCOs (M) | 35 | 3.6 |
| Total BUSCO groups searched | 978 | 100.0 |

**Table S6.** Summary of the genome assembly for ***Gigantopelta aegis***

|  | Scaffold | Contig |
| --- | --- | --- |
| Sequence number (>): | 1,970,755 | 2,111,450 |
| Total length of (bp): | 1,294,768,391 | 1,217,510,389 |
| Gap number (bp): | 77,258,002 | 0 |
| Average length (bp): | 656.99 | 576.62 |
| N50 Length (bp): | 120,955 | 6,964 |
| N90 Length (bp): | 129 | 125 |
| Maximum length (bp): | 1,890,902 | 115,520 |
| GC content is (%): | 37.45 | 37.45 |

**Table S7.** BUSCO assessment of the assembled genome for *Gigantopelta aegis* using the metazoa_odb9 database

| \ | Gene number | Percentage (%) |
| --- | --- | --- |
| Complete BUSCOs (C) | 904 | 92.4 |
| Complete and single-copy BUSCOs (S) | 883 | 90.3 |
| Complete and duplicated BUSCOs (D) | 21 | 2.1 |
| Fragmented BUSCOs (F) | 28 | 2.9 |
| Missing BUSCOs (M) | 46 | 4.7 |
| Total BUSCO groups searched | 978 | 100.0 |

**Table S8.** General statistics of predicted protein-coding genes of *Chrysomallon squamiferum*

| Gene set |  | Number | Average transcript length (bp) | Average CDS length (bp) | Average exon per gene | Average exon length (bp) | Average intron length (bp) |
| --- | --- | --- | --- | --- | --- | --- | --- |
| De novo | AUGUSTUS | 42,187 | 5067.34 | 1377.50 | 4.30 | 320.47 | 1118.68 |
| Homolog | *Aplysia_californica* | 28,063 | 3031.07 | 904.76 | 3.91 | 231.48 | 731.05 |
|  | *Biomphalaria_glabrata* | 35,494 | 2322.09 | 768.87 | 3.21 | 239.23 | 701.59 |
|  | *Lottiu_gigantea* | 35,987 | 2498.01 | 772.27 | 3.38 | 228.77 | 726.40 |
|  | *Pomacea_canaliculata* | 21,011 | 3025.20 | 850.89 | 3.72 | 228.79 | 799.65 |
|  | *Bathymodiolus_platifrons* | 32,138 | 2370.37 | 762.14 | 3.22 | 236.64 | 724.20 |
|  | *Modiolus_philippinarum* | 30,171 | 2217.80 | 737.59 | 3.07 | 240.48 | 716.04 |
| Final_set |  | 28,781 | 6651.90 | 1448.10 | 5.36 | 270.16 | 1193.49 |

**Table S9.** General Statistics of Predicted Protein-coding Genes of *Gigantopelta aegis*

| Gene set |  | Number | Average transcript length (bp) | Average CDS length (bp) | Average exon per gene | Average exon length (bp) | Average intron length (bp) |
| --- | --- | --- | --- | --- | --- | --- | --- |
| De novo | AUGUSTUS | 68,712 | 6,779.50 | 952.68 | 3.96 | 240.44 | 1,966.99 |
| Homolog | *Aplysia_californica* | 27,578 | 19,471.12 | 1708.58 | 8.69 | 196.71 | 2,311.14 |
|  | *Biomphalaria_glabrata* | 36,662 | 12,161.90 | 1,484.20 | 8.09 | 183.31 | 1,504.44 |
|  | *Lottiu_gigantea* | 23,822 | 4,502.48 | 1,136.72 | 5.93 | 191.82 | 777.50 |
|  | *Pomacea_canaliculata* | 21,383 | 8,458.72 | 1,493.31 | 8.29 | 180.14 | 966.34 |
|  | *Bathymodiolus_platifrons* | 33,584 | 9783.48 | 1,114.81 | 5.24 | 212.81 | 2,045.16 |
|  | *Modiolus_philippinarum* | 36,549 | 10,869.40 | 1,060.02 | 4.54 | 233.42 | 2,769.99 |
| GLEAN |  | 25,898 | 8,586.64 | 1,098.61 | 4.77 | 230.56 | 1,988.83 |
| Final_set |  | 25,601 | 8,617.09 | 1,101.84 | 4.77 | 230.84 | 1,991.72 |

**Table S10.** Summary of predicted gene functions of the *Chrysomallon squamiferum* gene set

| \ | Total | Swissprot | KEGG | TrEMBL | Interpro | Overall |
| --- | --- | --- | --- | --- | --- | --- |
| Gene Number | 28,781 | 21,967 | 25,624 | 27,830 | 21,087 | 28,438 |
| Percentage | 100% | 76.32% | 89.03% | 96.70% | 73.27% | 98.81% |

**Table S11.** Summary of predicted gene functions of the *Gigantopelta aegis* gene set

| \ | Total | Swissprot | KEGG | TrEMBL | Interpro | Overall |
| --- | --- | --- | --- | --- | --- | --- |
| Gene Number | 25,601 | 12,060 | 12,940 | 17,510 | 11,829 | 18,174 |
| Percentage | 100% | 47.11% | 50.54% | 68.40% | 46.21% | 70.99% |

**Table S12.** Summary of repeat contents in four selected species

| Type | *C. squamiferum* | | *P. canaliculate* | | *B. glabrata* | | *G.aegis* | |
| --- | --- | --- | --- | --- | --- | --- | --- | --- |
|  | Repeat Size (bp) | % of genome | Repeat Size (bp) | % of genome | Repeat Size (bp) | % of genome | Repeat Size (bp) | % of genome |
| Trf | 46,272,751 | 10.17933 | 12,733,523 | 2.89293 | 86,433,403 | 9.431965 | 122,167,938 | 9.435505 |
| Repeatmasker | 49,484,309 | 10.88582 | 16,818,853 | 3.82108 | 65,496,712 | 7.147268 | 73,533,494 | 5.679278 |
| Proteinmask | 3,473,397 | 0.764096 | 11,964,275 | 2.71817 | 58,450,475 | 6.378354 | 60,377,019 | 4.663152 |
| De novo | 113,792,349 | 25.03264 | 98,141,600 | 22.2968 | 397,147,606 | 43.338364 | 762,782,542 | 58.912663 |

**Table S13.** Gene family clusters in selected species

| Species | Total genes | Unclustered genes | Families | Unique families | Ave. genes per family |
| --- | --- | --- | --- | --- | --- |
| *Aplysia californica* | 19,786 | 4,822 | 10,029 | 287 | 1.49 |
| *Biomphalaria glabrata* | 25,424 | 5,040 | 11,853 | 766 | 1.72 |
| *Crassostrea gigas* | 28,355 | 3,459 | 12,071 | 970 | 2.06 |
| *Lottia gigantea* | 23,736 | 3,918 | 11,273 | 742 | 1.76 |
| *Octopus bimaculoides* | 15,823 | 2,562 | 8,813 | 238 | 1.5 |
| *Pomacea canaliculate* | 20,956 | 2,588 | 10,381 | 534 | 1.77 |
| *Pinctada fucata* | 32,937 | 4,346 | 11,792 | 1,316 | 2.42 |
| *Chrysomallon squamiferum* | 28,781 | 4,429 | 10,266 | 1,411 | 2.37 |
| *Gigantopelta aegis* | 25,601 | 6,585 | 7,894 | 1,479 | 2.41 |
| *Helobdella robusta* | 23,352 | 8,558 | 8,347 | 1,127 | 1.77 |

**Table S14.** Estimation of mutation rates of two deep-sea snails

| Species | Branch length (ML Tree Distance) | Branch length (Million year, Split Time Tree) | Mutation rate (substitution per site per year) |
| --- | --- | --- | --- |
| *C. squamiferum* | 0.186310436 | 66.3 | 2.81E-09 |
| *G. aegis* | 0.278017487 | 66.3 | 4.19E-09 |

**Table S15**. *K*a and *K*s values of 1,324 single copy orthologous genes from five snails

|  | *C. squamiferum* | *G. aegis* | *L. gigantea* | *P. canaliculate* | *B. glabrata* |
| --- | --- | --- | --- | --- | --- |
| *K*a | 0.373047 | 0.410202 | 0.351636 | 0.394091 | 0.416210 |
| *K*s | 3.349510 | 3.096858 | 3.717503 | 3.193140 | 3.242290 |
| *K*a/*K*s | 0.126270 | 0.151199 | 0.107906 | 0.139124 | 0.146528 |

**Table S16.** KEGG enrichment of expanded gene families of *C. squamiferum*

| MapID | MapTitle | Adjusted P-value | Enriched gene number |
| --- | --- | --- | --- |
| map01100 | Metabolic pathways | 4.58E-144 | 1303 |
| map00970 | Aminoacyl-tRNA biosynthesis | 1.23E-122 | 315 |
| map01200 | Carbon metabolism | 1.20E-76 | 381 |
| map01230 | Biosynthesis of amino acids | 2.16E-54 | 355 |
| map00010 | Glycolysis / Gluconeogenesis | 4.72E-40 | 161 |
| map00670 | One carbon pool by folate | 6.48E-40 | 122 |
| map00640 | Propanoate metabolism | 2.44E-30 | 100 |
| map00785 | Lipoic acid metabolism | 1.58E-25 | 28 |
| map01523 | Antifolate resistance | 2.78E-25 | 81 |
| map00020 | Citrate cycle (TCA cycle) | 1.76E-23 | 90 |
| map03018 | RNA degradation | 5.66E-23 | 92 |
| map00860 | Porphyrin and chlorophyll metabolism | 4.65E-22 | 78 |
| map00030 | Pentose phosphate pathway | 3.22E-21 | 94 |
| map00250 | Alanine, aspartate and glutamate metabolism | 6.49E-19 | 106 |
| map00220 | Arginine biosynthesis | 8.23E-14 | 68 |
| map00260 | Glycine, serine and threonine metabolism | 1.44E-13 | 98 |
| map00270 | Cysteine and methionine metabolism | 1.47E-13 | 84 |
| map00240 | Pyrimidine metabolism | 4.85E-13 | 105 |
| map03030 | DNA replication | 1.44E-11 | 38 |
| map00750 | Vitamin B6 metabolism | 4.51E-11 | 27 |
| map00730 | Thiamine metabolism | 6.45E-11 | 36 |
| map00630 | Glyoxylate and dicarboxylate metabolism | 8.93E-11 | 91 |
| map00780 | Biotin metabolism | 8.93E-11 | 49 |
| map00061 | Fatty acid biosynthesis | 4.32E-10 | 71 |
| map03010 | Ribosome | 2.79E-09 | 104 |
| map00983 | Drug metabolism - other enzymes | 1.42E-08 | 47 |
| map00230 | Purine metabolism | 1.10E-07 | 197 |
| map04066 | HIF-1 signaling pathway | 1.24E-07 | 43 |
| map00450 | Selenocompound metabolism | 4.53E-07 | 42 |
| map05418 | Fluid shear stress and atherosclerosis | 4.53E-07 | 60 |
| map04122 | Sulfur relay system | 4.56E-07 | 37 |
| map00040 | Pentose and glucuronate interconversions | 5.97E-07 | 36 |
| map04940 | Type I diabetes mellitus | 1.07E-06 | 18 |
| map00053 | Ascorbate and aldarate metabolism | 8.15E-06 | 25 |
| map00280 | Valine, leucine and isoleucine degradation | 1.73E-05 | 59 |
| map00620 | Pyruvate metabolism | 1.88E-05 | 90 |
| map00330 | Arginine and proline metabolism | 4.96E-05 | 57 |
| map00520 | Amino sugar and nucleotide sugar metabolism | 8.33E-05 | 83 |
| map01212 | Fatty acid metabolism | 0.000134128 | 74 |
| map00562 | Inositol phosphate metabolism | 0.00020273 | 35 |
| map03430 | Mismatch repair | 0.000423827 | 19 |
| map00480 | Glutathione metabolism | 0.000436014 | 56 |
| map00310 | Lysine degradation | 0.00529227 | 35 |
| map04212 | Longevity regulating pathway - worm | 0.016737238 | 32 |
| map04970 | Salivary secretion | 0.033521431 | 47 |

**Table S17.** KEGG enrichment of unique gene families in *G. aegis* genome

| MapID | MapTitle | Adjusted P-value | Enriched gene number |
| --- | --- | --- | --- |
| map05145 | Toxoplasmosis | 3.56E-202 | 329 |
| map05332 | Graft-versus-host disease | 4.93E-192 | 251 |
| map04672 | Intestinal immune network for IgA production | 1.43E-191 | 251 |
| map05310 | Asthma | 1.43E-191 | 251 |
| map05330 | Allograft rejection | 1.43E-191 | 251 |
| map05320 | Autoimmune thyroid disease | 6.09E-190 | 251 |
| map05321 | Inflammatory bowel disease (IBD) | 6.52E-187 | 252 |
| map04940 | Type I diabetes mellitus | 1.03E-186 | 251 |
| map04659 | Th17 cell differentiation | 1.76E-183 | 276 |
| map05140 | Leishmaniasis | 4.02E-180 | 253 |
| map05323 | Rheumatoid arthritis | 4.22E-179 | 252 |
| map04612 | Antigen processing and presentation | 1.55E-176 | 251 |
| map05322 | Systemic lupus erythematosus | 1.54E-173 | 251 |
| map04640 | Hematopoietic cell lineage | 4.87E-168 | 252 |
| map04658 | Th1 and Th2 cell differentiation | 7.72E-166 | 276 |
| map05150 | Staphylococcus aureus infection | 6.01E-165 | 251 |
| map05416 | Viral myocarditis | 6.54E-156 | 252 |
| map04514 | Cell adhesion molecules (CAMs) | 5.10E-150 | 253 |
| map05166 | HTLV-I infection | 7.21E-146 | 277 |
| map05168 | Herpes simplex infection | 2.64E-135 | 279 |
| map05164 | Influenza A | 9.79E-128 | 256 |
| map05152 | Tuberculosis | 2.88E-125 | 257 |
| map04145 | Phagosome | 1.19E-122 | 264 |
| map00051 | Fructose and mannose metabolism | 2.22E-86 | 339 |
| map04623 | Cytosolic DNA-sensing pathway | 6.67E-29 | 68 |
| map05222 | Small cell lung cancer | 7.02E-24 | 75 |
| map04215 | Apoptosis - multiple species | 4.92E-22 | 54 |
| map04621 | NOD-like receptor signaling pathway | 1.01E-19 | 102 |
| map04064 | NF-kappa B signaling pathway | 9.52E-16 | 53 |
| map03020 | RNA polymerase | 5.08E-14 | 46 |
| map04668 | TNF signaling pathway | 3.76E-13 | 52 |
| map05016 | Huntington's disease | 1.85E-12 | 82 |
| map04120 | Ubiquitin mediated proteolysis | 2.66E-09 | 60 |
| map01100 | Metabolic pathways | 3.98E-09 | 424 |
| map04210 | Apoptosis | 6.49E-08 | 68 |
| map05169 | Epstein-Barr virus infection | 1.45E-07 | 72 |
| map00240 | Pyrimidine metabolism | 1.43E-06 | 51 |
| map04950 | Maturity onset diabetes of the young | 2.08E-06 | 24 |
| map01524 | Platinum drug resistance | 8.72E-06 | 34 |
| map05221 | Acute myeloid leukemia | 9.09E-06 | 24 |
| map04624 | Toll and Imd signaling pathway | 4.62E-05 | 29 |
| map05220 | Chronic myeloid leukemia | 8.46E-05 | 25 |
| map04380 | Osteoclast differentiation | 0.00023214 | 28 |
| map04662 | B cell receptor signaling pathway | 0.00041004 | 28 |
| map04622 | RIG-I-like receptor signaling pathway | 0.00060659 | 24 |
| map04920 | Adipocytokine signaling pathway | 0.00060659 | 22 |
| map05131 | Shigellosis | 0.00142037 | 23 |
| map05142 | Chagas disease (American trypanosomiasis) | 0.00195435 | 26 |
| map04360 | Axon guidance | 0.0021697 | 34 |
| map05120 | Epithelial cell signaling in Helicobacter pylori infection | 0.00283449 | 24 |
| map04660 | T cell receptor signaling pathway | 0.0032753 | 25 |
| map05212 | Pancreatic cancer | 0.00439408 | 24 |
| map04657 | IL-17 signaling pathway | 0.00537642 | 24 |
| map05215 | Prostate cancer | 0.0059852 | 25 |
| map04620 | Toll-like receptor signaling pathway | 0.0064519 | 24 |
| map05160 | Hepatitis C | 0.00860911 | 28 |
| map04217 | Necroptosis | 0.01042632 | 36 |
| map04062 | Chemokine signaling pathway | 0.02226259 | 25 |
