# Supplementary material for: Genome sequencing of deep-sea hydrothermal vent snails reveals adaptions to extreme environments
Source: Gigascience. 2020 Dec 15;9(12):giaa139. doi: 10.1093/gigascience/giaa139 (PMC7736800; doi:10.1093/gigascience/giaa139)
Supplement: giaa139_Response_to_Reviewer_Comments_Original_Submission [file giaa139_response_to_reviewer_comments_original_submission.pdf]

Author's Response To Reviewer Comments

Close

Response to editors and reviewers:

Dear editor and reviewers:
On behalf of all the coauthors, we would like to thank you very much and all the reviewers for the time spent to assess our manuscript (GIGA-D-20-00187) and for your relevant remarks and suggestions that allow to improve the quality of this manuscript.
We have checked the manuscript carefully and revised it according to the comments.

Sincerely,
Xiang Zeng, Yaolei Zhang

Reviewer reports:
Reviewer #1: The Data Note by Zeng et al. reported two genome assemblies of deep sea gastropods, Chrysomallon squamiferum and Gigantopelta aegis.
I would ask the authors for additional informations about assembling process and references of data sources in order to guarantee the quality of the data and analyses. I also found there are many ambiguous expressions in the present manuscript, making it unclear how the genome resources can contribute to understand biology of these animals.
Please find my specific comments and concerns below, which need to be addressed.
Response: Thank you very much for your thoughtful and helpful suggestions. We have revised our manuscript according to your comments. Please see that.

Background
The authors should mention the fact that the Chrysomallon squamiferum genome has been published by Sun et al. (Nat Commun 11, 1657, 2020) somewhere in the Background section.
I would suggest the authors to explain that they analyzed the genome of "white scaly foot individual" while Sun et al. sequenced "black" one, to emphasize the uniqueness of this study.
Response: Thank you very much for this helpful suggestion. We have added descriptions as you suggested. Please see lines 91-93 "And the whole genome of black scaly-foot snail was reported recently, which highlighted its evolutionary mechanisms of biomineralised armour [9]" and 109-110: "In this study, we sequenced and assembled genomes of the white scaly-foot snail (Figure 1a), which is different from the published black individual".

Line 93
Remove "sp. nov.". This abbreviation is used when new species is named.
Response: Thank you. We removed this abbreviation. See lines 94-96: "The genus includes two species, Gigantopelta chessoia from East Scotia Ridge and Gigantopelta aegis from the Southwest Indian Ridge [6]. Both Chrysomallon and Gigantopelta are members of the family Peltospiridae."

Data description
Line 110
Was the insert size 350bp (main text) or 300bp (Table S2)?
Response: Thank you very much. We modified it as "350 bp" in Table S2.

Line 121-122
As mentioned above, the C. squamiferum genome has been published. Therefore this sentence needs to be removed.
Response: Thank you. We delete this sentence.

Line 156-160
These sentences do not make sense to me. Why despite "precise functions of these repeats have not been studied," the authors can infer the composition of repeat elements "may be closely associated with adaptation to extreme environment"? Please describe more specifically by mentioning some references

that support this idea.
Response: Thank you and sorry for confusing you. We added more description and relevant references about the importance of transposons/repeats. Please see lines: 156-161 “Although most of the precise functions of these repeats have not been studied in depth, repeats have been thought to have a regulatory function in related genes that play an important role in the life cycle and can introduce great genome flexibility [18]. And in the mammalian genome, transposons were described to be redundant enhancers that regulate their target genes which are higher or tissue specially expressed, indicating the importance of transposons”

Lines 173-175
It seems the authors assumed the split of C. squamiferum and G. aegis was related to the mass extinction event around 66 MYA. However, estimated divergence time is considerably ambiguous (42.4-100 MYA, Fig 1a), making the idea less reliable.
Response: Thank you. We agree with you and delete this description.

Deleted sentences: “This time is consistent with the most recent ‘mass extinction’, at the end of the Cretaceous geological period ~66 MYA, where ~76% of species became extinct” for your reference.

Lines 178-180
This sentence is difficult to understand. Speciation and demographic histories of each species are different topics.
Response: Thank you. We also delete this sentence.

Deleted sentences: “As the speciation of the two deep-sea snails may be related to geological events (see above)” for your reference.

Lines 190-191
This sentence is not clear. Please describe what "major geological events" affected the population sizes, with references describing the geological events.

An: Thank you. We added one example of geological events and relevant reference. Please see lines 194-197: “For example, the Cretaceous/Paleogene (K/Pg) extinction event caused extinction of three-quarters of species on earth and affected population dynamics approximately 66 million years ago, when an asteroid impact caused global environmental devastation [21, 22]”

Lines 191-193
The recent decreased population size was reported by ref[8] and was not related to this study (Fig2b). Then, this sentence may be put in the Background section.
An: Thank you. We deleted this sentence.

Deleted sentences: ” Unfortunately, the C. squamiferum population size has dramatically decreased recently due to deep-sea mining [8], which has made this species endangered” just for your reference

Lines 196-210
Comparisons of Ks and Ka values among snail species should be tested statistically. In the figures 2C and S3 it is not clear whether these values are significantly different.
Response: Thank you. We have added statistical test (Mann-Whitney U test) for Ka/Ks comparisons. Please see lines 204-213: “We found that the Ka values of the two deep-sea snails (average: 0.37 and 0.41) were higher (Mann-Whitney U test, p-value<0.001) than that of the shallow-water limpet (0.35) but similar to those of two freshwater snails (0.39 and 0.41), which suggests that the genes of deep-sea and freshwater snails both evolved faster after their divergence from shallow-water limpet . The Ks values of the deep-sea (3.34 and 3.09) and freshwater (3.19 and 3.24) snails were also similar and lower (Mann-Whitney U test, p-value<0.001) than those of the shallow-water limpet (3.72). Additionally, the Ka/Ks values of the deep-sea snails (average: 0.13 and 0.15) were approximately ~20% and ~40% higher (Mann-Whitney U test, p-value<0.001) than those of the shallow-water limpet (0.11)”.

Discussion
Lines 309-310
I have no idea what the "infamous Cambrian Explosion" means. Please explain the authors' idea more in detail.
Response: Thank you. It was modified to “Cambrian Explosion”. It was a clerical error. See line 319.

Lines 328-329
Please describe what are "adaptive needs" and "region-specific features" specifically.
Response: Thank you. We deleted this sentence.

Materials and Methods
Line 370
350bp or 300bp?
Response: It is “350 bp” actually for BGISEQ.

Lines 403-405
Based on the description, the 10X Chromium reads were used only for polishing, not for scaffolding. On the other hand, there are stats of scaffolds before Hi-C scaffolding in Table S3.
Response: Yes, the 10X Chromium reads were used only for polishing, not for scaffolding.

My questions are;
i) Were the scaffolds in Table S3 generated using 10X Chromium reads?
ii) If so, the scaffolds were improved very little (sequences are reduced from 6449 to 6444), indicating there was problem in 10X Chromium sequencing. How the authors interpreted the results?
Response: Thank you. The 6,444 scaffolds (also 6,444 contigs) were generated using Oxford Nanopore reads. We then used 10X Chromium reads to polish (error correction) 6,444 scaffolds with software Pilon. Pilon introduced 5 bp gaps so the contig number became 6,449 while the scaffold number was still 6,444.

Line 436
"Lottia"
Response: Thank you. We corrected this word.

Line 454
Describe a reference for GLEAN.
Response: Thank you. We added a reference for GLEAN. Please see line 465.

Lines 464-467
Describe sources or references for these genomic data.
Response: Thank you. We added sources for these genomic data. Please see lines 474-479: “ all the protein sequences from selected 10 representative species (8 species including Aplysia californica (GCF_000002075.1), Octopus bimaculoides (GCF_001194135.1), Biomphalaria glabrata (GCF_000457365.1), Crassostrea gigas (GCF_000297895.1), Lottia gigantea (GCF_000327385.1), Pomacea canaliculate (GCF_003073045.1), Pinctada fucata (GCA_002216045.1), Helobdella robusta (GCF_000326865.1) from NCBI database, C. squamiferum and G. aegis from this research) were compared using blastp with the E-value threshold set as 1e-7.”

Lines 484-487
Describe references of these fossil records rather than summary database (Timetree.org) so that readers can refer the original data.
Response: Thank you very much and we can’t agree with you more. However, each of the time point between two species refers a lot of references and TimeTree database summarized all of these references to estimate one divergence time with a confidence interval. For example, the divergence time between Aplysia californica and Crassostrea gigas was estimated to be 537 MYA with a confidence interval of 516.3-558.3 MYA based on 11 references. We used this confidence interval time to calibrate our estimation. This is a common method used frequently in nowadays genome research. So here we cite TimeTree database (Timetree.org) for reference which includes many references. However, if you think list all the references is a must, we are pleased to do this.

Methods of SNP identification and PSMC (lines 177-193) were not described.
Response: Thank you. We have added methods of SNP identification and PSMC. Please see lines 503-516: “SNP calling and estimation of history population sizes
About 50X clean WGS reads were mapped to genomes of C. squamiferum and G. aegis using BWA mem (v0.7.12-r1039) [73] with default parameters respecitvely. Then SAMtools (v0.1.19-44428cd) [74] and “SortSam.jar” in the picard package (v1.54) was used to convert and sort BAM files. Local realignment was again carried out using RealignerTargetCreator and IndelRealigner in GATK (v3.6) [75] with default parameters. SNPs were identified using HaplotypeCaller and filtered using VariantFiltration with

parameter "-filter-expression "QD < 2.0 || MQ < 40.0 || ReadPosRankSum < -8.0 || FS > 60.0" --filter-name LowQualFilter --genotype-filter-expression "DP < 5.0" --genotype-filter-name lt_5". Estimatation of history population sizes were carried out using PSMC (v0.6.5-r67) [76]. Firstly, diploid genome references were constructed using samtools and bcftools call with "samtools mpileup -C50" and "vcfutils.pl vcf2fq -d 20 -D 100". Secondly, the demographic history was inferred using PSMC with parameters '-N25 -t15 -r5 -p 4+25*2+4+6' [77]."

Fig 1a
Add the size of the scale for C. squamiferum. No scale is indicated for G.aegis.
Response: Thank you. Scale was added in Fig 1a. Also, we added "Scale bar = 1cm" in Fig 1 legend.
Fig 2a
This cartoon is too ambiguous and not suitable for scientific paper. The molecular phylogeny should be clearly shown by solid lines.
Response: Thank you. We have updated Fig. 2a. Please see that.

Fig.3d
This figure is not very informative for readers. The authors may want to draw molecular phylogeny trees for BTBD6 and HTR4.
Response: Thank you. We also deleted Fig.3d.

Reviewer #2: The manuscript entitled "Genome sequencing of deep-sea hydrothermal vent snails reveals adaptations to extreme environments" presents a nice description of a good genome assembly (16 chromosomes representing ~80% of the genome) of the scaly foot snail (Chrysomallon squamiferum) and compare it to genomes of other molluscan species. Overall the paper is well written and presents a nice view of some unique adaptations by this deep-sea mollusc. One concern that I had is throughout the manuscript (starting at line 164 and onward) the authors describe comparing two mussels, two freshwater snails and two shallow-water snails to their genomes. However, these other molluscan species include C. gigas and P. fucata…which are both oysters and not mussels, and while two of the other molluscs included in the tree are in Gastropoda and considered snails, Lottia gigantea is a limpet and Aplysia californica is a sea slug. I would encourage the authors to describe all of these species more accurately, i.e., as limpet and sea slug, because these are very different from what people commonly think of when they hear "snail", represented by the more traditional Pomacea and Biomphalaria. Referring to all the "snails" as gastropods would be a more suitable term that captures the true diversity of this large group. But when discussing individual species, I would prefer to see the more accurate descriptions because limpets and sea slugs are different from traditional snails, and will have unique adaptations of their own related to their unique characteristics. Overall, the authors give a good general description of the results and present a reasonable discussion about some of the potential adaptations that they observed in the genome. One minor point - thioredoxins are much more likely play a role in repairing proteins that have been altered by oxidation (Lines 255-256), so to limit this expansion to innate immunity leaves out a lot of other possibilities. My other question was regarding the source of the genomic DNA. The authors describe using muscle samples for isolating DNA, but it is not clear if DNA from one individual was used for all sequencing or if pooling occurred?
Response: Thank you very much for your approval and your thoughtful advices. We have updated our descriptions based on your suggestions. Please see below response.

1) About the scientific name (line 164 and onward), we modified this part as "we compared them with two shallow-water bivalves (P.fucata and C.gigas) and four shallow-water gastropods, including two fresh-water snails (B. glabrata and P. canaliculate), one limpet (B. glabrata) and one sea slug (A. californica). The California two-spot octopus (O. bimaculoides) and the freshwater leech (H.robusta) were used as the outgroup (Figure 2a) (lines 167-171)."
2) About thioredoxins, we added the description of thioredoxin as redox proteins and references. Please lines 260-265: "For example, increased expression of thioredoxin 1 (Txn1; 22 copies in C. squamiferum) was identified. Thioredoxin 1 (Txn1), a redox protein, is important in regulation of cellular redox homeostasis and anti-apoptotic functions. Txn1 stimulates cell proliferation and cell cycle progression, induces hypoxia-inducible factor-1α (HIF-1α) and angiogenesis, and alters the balance between the matrix metalloproteinases and their tissue inhibitors [29, 30]"
3) About isolating DNA, all DNA was isolated from one individual and we add clear description about this in lines 374-375: "DNA was extracted from muscle sample of one individual using the cetyl trimethylammonium bromide (CTAB) method and a DNeasy blood & tissue kit (QIAGEN)."

Reviewer #3: The manuscript of Zeng et al seems to describe a well-put together genome for one species of deep-sea snail, with an additional 'draft' genome for another species. It is clear and well-written, with most of the methods described sufficiently. My main criticism is that I found some of the discussions regarding the adaptative significance and/or putative "function" of various TE content and gene-family expansion results quite speculative, given that the comparative results are often observational with no hypothesis testing or statistical framework. That may well be beyond the remit of the paper, but the language could be more careful in places to reflect the putative nature of any hypothesised effects. Nonetheless I have no doubt that the genomes themselves will be useful additions to the community for future work on mollusc and animal evolution.
Response: Thank you very much for your approval of our manuscript and your helpful criticism. Yes, you are quite right. Here we did not show any experiments results to valid our hypothesis or speculation because this is a data description paper, mainly focusing on observational data. More investigations including both in-depth analysis and experiments based on these two genomes and these findings will be carried out in the future to verify function of important genes or TEs or conserved no-coding regions. These are important and interesting issues and must be done then. Thank you again.

Minor comments:
- Typo line 38: "impedes"
Response: Thank you. This word was modified

- Line 158: is there a reference or two for this? I would assume that most TEs are simply selfish genetic elements that do not serve a "function" per se but exist only for their own purpose, i.e. to copy themselves independently of the host genome
- Line 158-160: but most TE content differences are probably driven by stochastic forces (i.e. drift) rather than deterministic forces such as adaptation, and here we have only 2 data points. The language used for this statement is careful, but I wonder if it is too far to extrapolate that some differences in TE content may be adaptive
Response: Thank you very much for your helpful thoughts. We added more descriptions and references to support “TEs are functional” to make it clear. Please see lines 156-161: “Although most of the precise functions of these repeats have not been studied in depth, repeats have been thought to have a regulatory function in related genes that play an important role in the life cycle and can introduce great genome flexibility[18]. And in the mammalian genome, transposons were described to be redundant enhancers that regulate their target genes which are higher or tissue specially expressed, indicating the importance of transposon”.

- Line 196: I don't know what the authors mean by this statement
Response: Thank you. We deleted the sentence.

Deleted sentence “The evolution and expression of single-copy orthologous genes are unique features of organisms.” for your reference.

- Section on Ka/Ks values: there is no impression given about the statistical significance of the differences observed between Ka/Ks in any given lineage, or what the distribution of error looks like for these point estimates. Perhaps a more refined PAML analysis could resolve this? It is also not written how Ka/Ks values were calculated
Response: Thank you. We have added statistical test (Mann-Whitney U test) for Ka/Ks comparisons. Please see lines 204-213: “We found that the Ka values of the two deep-sea snails (average: 0.37 and 0.41) were higher (Mann-Whitney U test, p-value<0.001) than that of the shallow-water limpet (0.35) but similar to those of two freshwater snails (0.39 and 0.41), which suggests that the genes of deep-sea and freshwater snails both evolved faster after their divergence from shallow-water limpet . The Ks values of the deep-sea (3.34 and 3.09) and fresh-water (3.19 and 3.24) snails were also similar and lower (Mann-Whitney U test, p-value<0.001) than those of the shallow-water limpet (3.72). Additionally, the Ka/Ks values of the deep-sea snails (average: 0.13 and 0.15) were approximately ~20% and ~40% higher (Mann-Whitney U test, p-value<0.001) than those of the shallow-water limpet (0.11).

And the Ka/Ks values were calculated actually using codeml in PAML package. We have added this information to lines 200-204: “To explore the evolutionary rate of single-copy orthologous genes, we calculated the synonymous substitution rate (Ka) and nonsynonymous substitution rate (Ks) values of 1,324 single-copy orthologous genes shared by the two deep-sea snails, one shallow-water limpet (L. gigantea), and two freshwater snails (B. glabrata and P. canaliculate) using Codeml in PAML package[23].”

- Typo line 214: CAFE not CAFÉ
Response: Thank you. It was modified.
- Line 329: "region-specific feature shared between lineages" - not sure what is meant by this?
Response: Thank you. We deleted this confusing sentence.
- Line 350: it seems speculative - surely both immune response and biomineralization are "vital" for all snails, not particularly deep-sea ones?
Response: Thank you. We modified it and specified it in C. squamiferum which is a chemosynthetic snail species depending on endosymbionts. See lines 360-362 "In particular, we found that DMBT1 gene families that encode multiple SRCR domains expanded significantly in C. squamiferum. These genes play important roles in immune response and biomineralization, both of which are vital for deep sea chemosynthetic snail".

- Line 454: reference for GLEAN is missing
Response: Thank you. We added this reference (65). See line 465

- Line 469: references for Solar and Hcluster are missing, and what is a H-score?
Response: Thank you. Solar, Hcluster_sg and H-score are tools and concept of TreeFam tools. We added reference for Solar and Hcluster_sg. H-score means hcluster score. Details can be found from TreeFam tools.

- Figure 2a: it's a weird looking tree that, in fact, looks a bit like a snail itself! Are the widths of the blobs representative of the error around the divergence times or topological support?
Response: Thank you and sorry for confusing you. We updated Fig.2a. Please see that.

Close
