# Supplementary material for: Genome sequencing of deep-sea hydrothermal vent snails reveals adaptions to extreme environments
Source: Gigascience. 2020 Dec 15;9(12):giaa139. doi: 10.1093/gigascience/giaa139 (PMC7736800; doi:10.1093/gigascience/giaa139)
Supplement: giaa139_Reviewer_1_Report_Original_Submission — Takeshi Takeuchi, Ph.D -- 7/31/2020 Reviewed [file giaa139_reviewer_1_report_original_submission.pdf]

Reviewer Report

Title: Genome sequencing of deep-sea hydrothermal vent snails reveals adaptations to extreme environments

Version: Original Submission **Date:** 7/31/2020

Reviewer name: Takeshi Takeuchi, Ph.D

Reviewer Comments to Author:

The Data Note by Zeng et al. reported two genome assemblies of deep sea gastropods, Chrysomallon squamiferum and Gigantopelta aegi.
I would ask the authors for additional informations about assembling process and references of data sources in order to guarantee the quality of the data and analyses. I also found there are many ambiguous expressions in the present manuscript, making it unclear how the genome resources can contribute to understand biology of these animals.
Please find my specific comments and concerns below, which need to be addressed.
Background
The authors should mention the fact that the Chrysomallon squamiferum genome has been published by Sun et al. (Nat Commun 11, 1657, 2020) somewhere in the Background section.
I would suggest the authors to explain that they analyzed the genome of "white scaly foot individual" while Sun et al. sequenced "black" one, to emphasize the uniqueness of this study.
Line 93
Remove "sp. nov.". Thisã€€abbreviation is used when new species is named.
Data description
Line 110
Was the insert size 350bp (main text) or 300bp (Table S2)?
Line 121-122
As mentioned above, the C. squamiferum genome has been published. Therefore this sentence needs to be removed.
Line 156-160
These sentences do not make sense to me. Why despite "precise functions of these repeats have not been studied," the authors can infer the composition of repeat elements "may be closely associated with adaptation to extreme environment"? Please describe more specifically by mentioning some references that support this idea.
Lines 173-175
It seems the authors assumed the split of C. squamiferum and G. aegis was related to the mass extinction event around 66 MYA. However, estimated divergence time is considerably ambiguous (42.4-100 MYA, Fig 1a), making the idea less reliable.
Lines 178-180
This sentence is difficult to understand. Speciation and demographic histories of each species are different topics.

Lines 190-191
This sentence is not clear. Please describe what "major geological events" affected the population sizes, with references describing the geological events.
Lines 191-193
The recent decreased population size was reported by ref[8] and was not related to this study (Fig2b). Then, this sentence may be put in the Background section.
Lines 196-210
Comparisons of Ks and Ka values among snail species should be tested statistically. In the figures 2C and S3 it is not clear whether these values are significantly different.
Discussion
Lines 309-310
I have no idea what the "infamous Cambrian Explosion" means. Please explain the authors' idea more in detail.
Lines 328-329
Please describe what are "adaptive needs" and "region-specific features" specifically.
Materials and Methods
Line 370
350bp or 300bp?
Lines 403-405
Based on the description, the 10X Chromium reads were used only for polishing, not for scaffolding. On the other hand, there are stats of scaffolds before Hi-C scaffolding in Table S3.
My questions are;
i) Were the scaffolds in Table S3 generated using 10X Chromium reads?
ii) If so, the scaffolds were improved very little(sequences are reduced from 6449 to 6444), indicating there was problem in 10X Chromium sequencing. How the authors interpreted the results?
Line 436
"Lottia"
Line 454
Describe a reference for GLEAN.
Lines 464-467
Describe sources or references for these genomic data.
Lines 484-487
Describe references of these fossil records rather than summary database (Timetree.org) so that readers can refer the original data.
Methods of SNP identification and PSMC (lines 177-193) were not described.
Fig 1a
Add the size of the scale for C. squamiferum. No scale is indicated for G.aegis.
Fig 2a
This cartoon is too ambiguous and not suitable for scientific paper. The molecular phylogeny should be clearly shown by solid lines.
Fig.3d

This figure is not very informative for readers. The authors may want to draw molecular phylogeny trees for BTBD6 and HTR4.

Level of Interest

Please indicate how interesting you found the manuscript: Choose an item.

Quality of Written English

Please indicate the quality of language in the manuscript: Choose an item.

Declaration of Competing Interests

Please complete a declaration of competing interests, considering the following questions:

- Have you in the past five years received reimbursements, fees, funding, or salary from an organisation that may in any way gain or lose financially from the publication of this manuscript, either now or in the future?
- Do you hold any stocks or shares in an organisation that may in any way gain or lose financially from the publication of this manuscript, either now or in the future?
- Do you hold or are you currently applying for any patents relating to the content of the manuscript?
- Have you received reimbursements, fees, funding, or salary from an organization that holds or has applied for patents relating to the content of the manuscript?
- Do you have any other financial competing interests?
- Do you have any non-financial competing interests in relation to this paper?

If you can answer no to all of the above, write 'I declare that I have no competing interests' below. If your reply is yes to any, please give details below.

I declare that I have no competing interests.

I agree to the open peer review policy of the journal. I understand that my name will be included on my report to the authors and, if the manuscript is accepted for publication, my named report including any attachments I upload will be posted on the website along with the authors' responses. I agree for my report to be made available under an Open Access Creative Commons CC-BY license (http://creativecommons.org/licenses/by/4.0/). I understand that any comments which I do not wish to be included in my named report can be included as confidential comments to the editors, which will not be published.

Choose an item.

To further support our reviewers, we have joined with Publons, where you can gain additional credit to further highlight your hard work (see: https://publons.com/journal/530/gigascience). On publication of this paper, your review will be automatically added to Publons, you can then choose whether or not to claim your Publons credit. I understand this statement.

Yes Choose an item.
