# Supplementary material for: Genome sequencing of deep-sea hydrothermal vent snails reveals adaptions to extreme environments
Source: Gigascience. 2020 Dec 15;9(12):giaa139. doi: 10.1093/gigascience/giaa139 (PMC7736800; doi:10.1093/gigascience/giaa139)
Supplement: giaa139_Reviewer_2_Report_Original_Submission — Mathew J. Jenny -- 8/5/2020 Reviewed [file giaa139_reviewer_2_report_original_submission.pdf]

Reviewer Report

Title: Genome sequencing of deep-sea hydrothermal vent snails reveals adaptations to extreme environments

Version: Original Submission **Date:** 8/5/2020

Reviewer name: Mathew J. Jenny

Reviewer Comments to Author:

The manuscript entitled "Genome sequencing of deep-sea hydrothermal vent snails reveals adaptations to extreme environments" presents a nice description of a good genome assembly (16 chromosomes representing ~80% of the genome) of the scaly foot snail (Chrysomallon squamiferum) and compare it to genomes of other molluscan species. Overall the paper is well written and presents a nice view of some unique adaptations by this deep-sea mollusc. One concern that I had is throughout the manuscript (starting at line 164 and onward) the authors describe comparing two mussels, two freshwater snails and two shallow-water snails to their genomes. However, these other molluscan species include C. gigas and P. fucata…which are both oysters and not mussels, and while two of the other molluscs included in the tree are in Gastropoda and considered snails, Lottia gigantea is a limpet and Aplysia californica is a sea slug. I would encourage the authors to describe all of these species more accurately, i.e., as limpet and sea slug, because these are very different from what people commonly think of when they hear "snail", represented by the more traditional Pomacea and Biomphalaria. Referring to all the "snails" as gastropods would be a more suitable term that captures the true diversity of this large group. But when discussing individual species, I would prefer to see the more accurate descriptions because limpets and sea slugs are different from traditional snails, and will have unique adaptations of their own related to their unique characteristics. Overall, the authors give a good general description of the results and present a reasonable discussion about some of the potential adaptations that they observed in the genome. One minor point - thioredoxins are much more likely play a role in repairing proteins that have been altered by oxidation (Lines 255-256), so to limit this expansion to innate immunity leaves out a lot of other possibilities. My other question was regarding the source of the genomic DNA. The authors describe using muscle samples for isolating DNA, but it is not clear if DNA from one individual was used for all sequencing or if pooling occurred?

Level of Interest

Please indicate how interesting you found the manuscript: Choose an item.

Quality of Written English

Please indicate the quality of language in the manuscript: Choose an item.

Declaration of Competing Interests

Please complete a declaration of competing interests, considering the following questions:

- Have you in the past five years received reimbursements, fees, funding, or salary from an organisation that may in any way gain or lose financially from the publication of this manuscript, either now or in the future?
- Do you hold any stocks or shares in an organisation that may in any way gain or lose financially from the publication of this manuscript, either now or in the future?
- Do you hold or are you currently applying for any patents relating to the content of the manuscript?
- Have you received reimbursements, fees, funding, or salary from an organization that holds or has applied for patents relating to the content of the manuscript?
- Do you have any other financial competing interests?
- Do you have any non-financial competing interests in relation to this paper?

If you can answer no to all of the above, write 'I declare that I have no competing interests' below. If your reply is yes to any, please give details below.

I declare that I have no competing interests.

I agree to the open peer review policy of the journal. I understand that my name will be included on my report to the authors and, if the manuscript is accepted for publication, my named report including any attachments I upload will be posted on the website along with the authors' responses. I agree for my report to be made available under an Open Access Creative Commons CC-BY license (http://creativecommons.org/licenses/by/4.0/). I understand that any comments which I do not wish to be included in my named report can be included as confidential comments to the editors, which will not be published.

Choose an item.

To further support our reviewers, we have joined with Publons, where you can gain additional credit to further highlight your hard work (see: https://publons.com/journal/530/gigascience). On publication of this paper, your review will be automatically added to Publons, you can then choose whether or not to claim your Publons credit. I understand this statement.

Yes Choose an item.
