# Supplementary material for: Genome sequencing of deep-sea hydrothermal vent snails reveals adaptions to extreme environments
Source: Gigascience. 2020 Dec 15;9(12):giaa139. doi: 10.1093/gigascience/giaa139 (PMC7736800; doi:10.1093/gigascience/giaa139)
Supplement: giaa139_Reviewer_3_Report_Original_Submission — Reuben William Nowell, Ph.D. -- 8/10/2020 Reviewed [file giaa139_reviewer_3_report_original_submission.pdf]

Reviewer Report

Title: Genome sequencing of deep-sea hydrothermal vent snails reveals adaptations to extreme environments

Version: Original Submission **Date:** 8/10/2020

Reviewer name: Reuben William Nowell, Ph.D.

Reviewer Comments to Author:

The manuscript of Zeng et al seems to describe a well-put together genome for one species of deep-sea snail, with an additional 'draft' genome for another species. It is clear and well-written, with most of the methods described sufficiently. My main criticism is that I found some of the discussions regarding the adaptative significance and/or putative "function" of various TE content and gene-family expansion results quite speculative, given that the comparative results are often observational with no hypothesis testing or statistical framework. That may well be beyond the remit of the paper, but the language could be more careful in places to reflect the putative nature of any hypothesised effects. Nonetheless I have no doubt that the genomes themselves will be useful additions to the community for future work on mollusc and animal evolution.

Minor comments:

- Typo line 38: "impedes"
- Line 158: is there a reference or two for this? I would assume that most TEs are simply selfish genetic elements that do not serve a "function" per se but exist only for their own purpose, i.e. to copy themselves independently of the host genome
- Line 158-160: but most TE content differences are probably driven by stochastic forces (i.e. drift) rather than deterministic forces such as adaptation, and here we have only 2 data points. The language used for this statement is careful, but I wonder if it is too far to extrapolate that some differences in TE content may be adaptive
- Line 196: I don't know what the authors mean by this statement
- Section on Ka/Ks values: there is no impression given about the statistical significance of the differences observed between Ka/Ks in any given lineage, or what the distribution of error looks like for these point estimates. Perhaps a more refined PAML analysis could resolve this? It is also not written how Ka/Ks values were calculated
- Typo line 214: CAFE not CAFÃ‰
- Line 329: "region-specific feature shared between lineages" - not sure what is meant by this?
- Line 350: it seems speculative - surely both immune response and biomineralization are "vital" for all snails, not particularly deep-sea ones?
- Line 454: reference for GLEAN is missing
- Line 469: references for Solar and Hcluster are missing, and what is a H-score?
- Figure 2a: it's a weird looking tree that, in fact, looks a bit like a snail itself! Are the widths of the blobs representative of the error around the divergence times or topological support?

Level of Interest

Please indicate how interesting you found the manuscript: Choose an item.

Quality of Written English

Please indicate the quality of language in the manuscript: Choose an item.

Declaration of Competing Interests

Please complete a declaration of competing interests, considering the following questions:

- Have you in the past five years received reimbursements, fees, funding, or salary from an organisation that may in any way gain or lose financially from the publication of this manuscript, either now or in the future?
- Do you hold any stocks or shares in an organisation that may in any way gain or lose financially from the publication of this manuscript, either now or in the future?
- Do you hold or are you currently applying for any patents relating to the content of the manuscript?
- Have you received reimbursements, fees, funding, or salary from an organization that holds or has applied for patents relating to the content of the manuscript?
- Do you have any other financial competing interests?
- Do you have any non-financial competing interests in relation to this paper?

If you can answer no to all of the above, write 'I declare that I have no competing interests' below. If your reply is yes to any, please give details below.

I declare that I have no competing interests

I agree to the open peer review policy of the journal. I understand that my name will be included on my report to the authors and, if the manuscript is accepted for publication, my named report including any attachments I upload will be posted on the website along with the authors' responses. I agree for my report to be made available under an Open Access Creative Commons CC-BY license (http://creativecommons.org/licenses/by/4.0/). I understand that any comments which I do not wish to be included in my named report can be included as confidential comments to the editors, which will not be published.

Choose an item.

To further support our reviewers, we have joined with Publons, where you can gain additional credit to further highlight your hard work (see: https://publons.com/journal/530/gigascience). On publication of

this paper, your review will be automatically added to Publons, you can then choose whether or not to claim your Publons credit. I understand this statement.

Yes Choose an item.
